# Supplementary material for: Compensatory Modulation of Seed Storage Protein Synthesis and Alteration of Starch Accumulation by Selective Editing of 13 kDa Prolamin Genes by CRISPR-Cas9 in Rice
Source: Int J Mol Sci. 2024 Jun 14;25(12):6579. doi: 10.3390/ijms25126579 (PMC11204006; doi:10.3390/ijms25126579)
Supplement: Supplementary file 1 [file ijms-25-06579-s001.zip › Supplementary Figures.zip!/Figure S8.pdf]

Figure S8. Sequence alignment of all the 13 kDa prolamins genes.

```
pro13b1         ------------------------------------------------------------      0
pro13b2         ------------------------------------------------------------      0
pro13b3         ------------------------------------------------------------      0
pro13b4         ------------------------------------------------------------      0
pro13b22        ------------------------------------------------------------      0
pro13b21        ------------------------------------------------------------      0
pro13b19        ------------------------------------------------------------      0
pro13b20        ------------------------------------------------------------      0
pro13b6         ------------------------------------------------------------      0
pro13b5         ------------------------------------------------------------      0
pro13b7         ------------------------------------------------------------      0
pro13b18        ------------------------------------------------------------      0
pro13b14        ------------------------------------------------------------      0
pro13b8         ------------------------------------------------------------      0
pro13b17        ------------------------------------------------------------      0
pro13b16        ------------------------------------------------------------      0
pro13b15        ------------------------------------------------------------      0
pro13b13        ------------------------------------------------------------      0
pro13b9         ------------------------------------------------------------      0
pro13b10        ------------------------------------------------------------      0
pro13b11        ------------------------------------------------------------      0
pro13b12        ------------------------------------------------------------      0
pro13a1         ------------------------------------------------------------      0
pro13a2         ------------------------------------------------------------      0
pro13a5         ATGGCGGGAGGCGGCGGGATTCGCGGCGGCGGCGGCGCTAGGGCACGGGCGAGGCGGCGG      60
pro13a3         ------------------------------------------------------------      0
pro13a4         ------------------------------------------------------------      0
pro13a6         ------------------------------------------------------------      0

pro13b1         ------------------------------------------------------------      0
pro13b2         ------------------------------------------------------------      0
pro13b3         ------------------------------------------------------------      0
pro13b4         ------------------------------------------------------------      0
pro13b22        ------------------------------------------------------------      0
pro13b21        ------------------------------------------------------------      0
pro13b19        ------------------------------------------------------------      0
pro13b20        ------------------------------------------------------------      0
pro13b6         ------------------------------------------------------------      0
pro13b5         ------------------------------------------------------------      0
pro13b7         ------------------------------------------------------------      0
pro13b18        ------------------------------------------------------------      0
pro13b14        ------------------------------------------------------------      0
pro13b8         ------------------------------------------------------------      0
pro13b17        ------------------------------------------------------------      0
pro13b16        ------------------------------------------------------------      0
pro13b15        ------------------------------------------------------------      0
pro13b13        ------------------------------------------------------------      0
pro13b9         ------------------------------------------------------------      0
pro13b10        ------------------------------------------------------------      0
pro13b11        ------------------------------------------------------------      0
pro13b12        ------------------------------------------------------------      0
pro13a1         ------------------------------------------------------------      0
pro13a2         ------------------------------------------------------------      0
pro13a5         GAGCGGCGGGAGGAAGGGGATGACGGGTGGGCCCCACCCGTCAGCGAGGGTGGCGGGCGG      120
pro13a3         ------------------------------------------------------------      0
pro13a4         ------------------------------------------------------------      0
pro13a6         ------------------------------------------------------------      0
```

```
pro13b1       ------------------------------------------------------------      0
pro13b2       ------------------------------------------------------------      0
pro13b3       ------------------------------------------------------------      0
pro13b4       ------------------------------------------------------------      0
pro13b22      ------------------------------------------------------------      0
pro13b21      ------------------------------------------------------------      0
pro13b19      ------------------------------------------------------------      0
pro13b20      ------------------------------------------------------------      0
pro13b6       ------------------------------------------------------------      0
pro13b5       ------------------------------------------------------------      0
pro13b7       ------------------------------------------------------------      0
pro13b18      ------------------------------------------------------------      0
pro13b14      ------------------------------------------------------------      0
pro13b8       ------------------------------------------------------------      0
pro13b17      ------------------------------------------------------------      0
pro13b16      ------------------------------------------------------------      0
pro13b15      ------------------------------------------------------------      0
pro13b13      ------------------------------------------------------------      0
pro13b9       ------------------------------------------------------------      0
pro13b10      ------------------------------------------------------------      0
pro13b11      ------------------------------------------------------------      0
pro13b12      ------------------------------------------------------------      0
pro13a1       ------------------------------------------------------------      0
pro13a2       ------------------------------------------------------------      0
pro13a5       GCCCGCCCGTCAGCGGCACGCGCGCGGGGAGGAGGCCGAATGGGCCGCGGAGGAGAGGAG      180
pro13a3       ------------------------------------------------------------      0
pro13a4       ------------------------------------------------------------      0
pro13a6       ------------------------------------------------------------      0

pro13b1       ------------------------------------------------------------      0
pro13b2       ------------------------------------------------------------      0
pro13b3       ------------------------------------------------------------      0
pro13b4       ------------------------------------------------------------      0
pro13b22      ------------------------------------------------------------      0
pro13b21      ------------------------------------------------------------      0
pro13b19      ------------------------------------------------------------      0
pro13b20      ------------------------------------------------------------      0
pro13b6       ------------------------------------------------------------      0
pro13b5       ------------------------------------------------------------      0
pro13b7       ------------------------------------------------------------      0
pro13b18      ------------------------------------------------------------      0
pro13b14      ------------------------------------------------------------      0
pro13b8       ------------------------------------------------------------      0
pro13b17      ------------------------------------------------------------      0
pro13b16      ------------------------------------------------------------      0
pro13b15      ------------------------------------------------------------      0
pro13b13      ------------------------------------------------------------      0
pro13b9       ------------------------------------------------------------      0
pro13b10      ------------------------------------------------------------      0
pro13b11      ------------------------------------------------------------      0
pro13b12      ------------------------------------------------------------      0
pro13a1       ------------------------------------------------------------      0
pro13a2       ------------------------------------------------------------      0
pro13a5       AGAGAGGGAGAGAGATGGGCCGAGCCGGCCCAAGAGGGGGAGGAGGGGGAAAAGGACTTT      240
pro13a3       ------------------------------------------------------------      0
pro13a4       ------------------------------------------------------------      0
pro13a6       ------------------------------------------------------------      0
```

```
pro13b1     ------------------------------------------------------------     0
pro13b2     ------------------------------------------------------------     0
pro13b3     ------------------------------------------------------------     0
pro13b4     ------------------------------------------------------------     0
pro13b22    ------------------------------------------------------------     0
pro13b21    ------------------------------------------------------------     0
pro13b19    ------------------------------------------------------------     0
pro13b20    ------------------------------------------------------------     0
pro13b6     ------------------------------------------------------------     0
pro13b5     ------------------------------------------------------------     0
pro13b7     ------------------------------------------------------------     0
pro13b18    ------------------------------------------------------------     0
pro13b14    ------------------------------------------------------------     0
pro13b8     ------------------------------------------------------------     0
pro13b17    ------------------------------------------------------------     0
pro13b16    ------------------------------------------------------------     0
pro13b15    ------------------------------------------------------------     0
pro13b13    ------------------------------------------------------------     0
pro13b9     ------------------------------------------------------------     0
pro13b10    ------------------------------------------------------------     0
pro13b11    ------------------------------------------------------------     0
pro13b12    ------------------------------------------------------------     0
pro13a1     ------------------------------------------------------------     0
pro13a2     ------------------------------------------------------------     0
pro13a5     TTTTAGGGTTTTTCTTTTTATAAAACCTTTTTAACTTTGTTTATTTCTTCTTAATTATTA     300
pro13a3     -----------------------------------------------------ATCATTC     7
pro13a4     --------------------------------------------------TTCATTCTTC     10
pro13a6     ------------------------------------------------------------     0


pro13b1     ------------------------------------------------------------     0
pro13b2     ---------------TCATCGCTCGCACAATTCAAGCATTATACAACAAAAATTTAAAAG     45
pro13b3     -----------------ATCGCTCGCACAATTCAAGCATTATACAACAAAAATTTAAAAG     43
pro13b4     -----------------ATCGCTCGCACAATTCAAGCATTATACAACAAAAATTTAAAAG     43
pro13b22    --------------------GTTCACACAGTTCAAGCATTATACAG-TAA-AAAAGAAAG     38
pro13b21    ----------------CATCGTTCACACAGTTCAAGCATTATACAG-CAA-AAAAGAAAG     42
pro13b19    -----------------ATCGTTCACACAGTTCAAGCATTATACAG-CAA-GAAAGAAAG     41
pro13b20    -----------------ATCGTTCACACAGTTCAAGCATTATACAACAAA-AAAAGAAAG     42
pro13b6     ------------------------------------------------------------     0
pro13b5     ---------------------------CAGTTCAAGCATTATACAG-CAA-AAAAGAAAG     31
pro13b7     ------------------TCGTTCACACAGTTCAAGCATTATACAA-CAA-AATAGAAAG     40
pro13b18    -------------------------------------------------------GAAAG     5
pro13b14    ------------------TCGTTCACACAGTTCAAGCATTATACAG-CAA-AATAGAAAG     40
pro13b8     --------------CTCATCATTCACACAGTTCAAGCATTATTCAG-CAA-AATAGAAAG     44
pro13b17    ------------------TCGTTCACACAGTTCAAGCATTATACAG-CAA-AATAGAAAG     40
pro13b16    ------------------TCGTTCACACAGTTCAAGCATTATACAG-CAA-AATAGAAAG     40
pro13b15    --------------------------------------------AG-CAA-AATAGAAAG     14
pro13b13    --------------------------------------TTATACAG-CAA-AATAGAAAG     20
pro13b9     ------------------TCGTTCACACAGTTCAAGCATTATACAG-CAA-AATAGAAAG     40
pro13b10    ---------------------------CAGTTCAAGCATTATACAG-CAA-AATAGAAAG     31
pro13b11    ------------------TCGTTCACACAGTTCAAGCATTATACAG-CAA-AATAGAAAG     40
pro13b12    ------------------TCGTTCACACAGTTCAAGCATTATACAG-CAA-AATAGAAAG     40
pro13a1     -----------TCATCATCCTTCACCACAATTCAAATAT--T---ATAGTTGAAGCATAG     44
pro13a2     ---------------TCATCCTTCACACAATTCAAACAT--T---ATAGTTGAAGCATAG     40
pro13a5     TTTGTGCTCTGAAAATTCCACTAAAATTTATTTACGCATTTTA--GGCTTTTAGGAAAT-     357
pro13a3     TCACAACTCAAACATTACA-----------------------------GCAAAAGCATAG     39
pro13a4     TCACAACTCAAACATTACA-----------------------------GCAAAAGCATAG     42
pro13a6     ------------------------------------------------------------     0
```

```
pro13b1         ------------------ATGAAGATCAT-TTTCGTCTTTGCTCTCCTTGCTATTGCTGC      41
pro13b2         AACTAGTGTCCTGCAACAATGAAGATCAT-TTTCGTCTTTGCTCTCCTTGCTATTGCTGC      104
pro13b3         AACTAGTGTCCTGCAACAATGAAGATCAT-TTTCGTCTTTGCTCTCCTTGCTATTGCTGC      102
pro13b4         AACTAGTGTCCTGCAACAATGAAGATCAT-TTTCGTCTTTGCTCTCCTTGCTATTGCTGC      102
pro13b22        ATCTAGTGTCCTGTCGCAATGAAGATCAT-TTTCATCTTTGCTCTTCTTGCTATTGCTGC      97
pro13b21        ATCTAGTGTCCCGTAGCAATGAAGATCAT-TTTCGCCTTTGCTCTTCTTACTATTGCTGC      101
pro13b19        ATCTAGTGTCCCGTAGCAATGAAGATCAT-TTTCGTCTTTGCTCTTCTTGCTATTGCTGC      100
pro13b20        ATCTAGTGTCCCGTAGCAATGAAGATCAT-TTTCGTCTTTGCTCTTCTTGCTATTGCTGC      101
pro13b6         ----------------------------AGATCATTTTCGTCTTTGCTTGCTATTGCTGC      32
pro13b5         ATCTAGTGTCCCGCAGCAATGAAGATCATTTTTCGTCTTTGCTCTCCTTGCTATTGCTGC      91
pro13b7         ATCTAGTGTCCCGTAGCAATGAAGATCCT-TTTCGTCTTTGCGCTCCTTGCTATTGCTGC      99
pro13b18        ATCTAGTGTTCCGCAGCAATGAAGATCAT-TTTCGTCTTTGCTCTCCTTGCTATTGCTGC      64
pro13b14        ATCTAGTGTCCCGCAGCAATGAAGATCAT-TTTCGTCTTTGCTCTCCTTGCTATTGCTGC      99
pro13b8         ATCTAGTGTCCCGCAGCAATGAAGATCAT-TTTCGTCTTTGCTCTCCTTGCTATTGCTGC      103
pro13b17        ATCTAGTGTCCCGCAGCAATGAAGATCAT-TTTCGTCTTTGCTCTCCTTGCTATTGCTGC      99
pro13b16        ATCTAGTGTCCCGCAGCAATGAAGATCAT-TTTCGTCTTTGCTCTCCTTGCTATTGCTGC      99
pro13b15        ATCTAGTGTCCCGCAGCAATGAAGATCAT-TTTCGTCTTTGCTCTCCTTGCTATTGCTGC      73
pro13b13        ATCTAGTGTCCCGCAGCAATGAAGATCAT-TTTCGTCTTTGCTCTCCTTGCTATTGCTGC      79
pro13b9         ATCTAGTGTCCCGCAGCAATGAAGATCAT-TTTCGTCTTTGCTCTCCTTGCTATTGCTGC      99
pro13b10        ATCTAGTGTCCCGCAGCAATGAAGATCAT-TTTCGTCTTTGCTCTCCTTGCTATTGCTGC      90
pro13b11        ATCTAGTGTCCCGCAGCAATGAAGATCAT-TTTCGTCTTTGCTCTCCTTGCTATTGCTGC      99
pro13b12        ATCTAGTGTCCCGCAGCAATGAAGATCAT-TTTCGTCTTTGCTCTCCTTGCTATTGCTGC      99
pro13a1         TAGTAGAATCCAACAACAATGAAGATCAT-TTTCGTATTTGCTCTCCTTGCTATTGTTGC      103
pro13a2         TAGTAGAATCCTACAAAAATGAAGATCAT-TTTCGTATTTGCTCTCCTTGCTATTGTTGC      99
pro13a5         ATACAAAAATCCTCCAAGCCTTATTTGAC-TTTTAACTTTGCA--CATTTTAATTGTTGG      414
pro13a3         AACTAGAAACCCACCACAATGAAGATCAT-TTTCTTCTTTGCTCTCCTTGCTATTGCTGC      98
pro13a4         AACTAGAAACCCACCACAATGAAGATCAT-TTTCTTCTTTGCTCTCCTTGCTATTGCTGC      101
pro13a6         ------------------ATGAAGATCAT-TTTCTTCTTTGCTCTCCTTGCTATTGCTGC      41
                               *     *   *     **   **** **

pro13b1         ATGCAGCGCCACTGCGCAGTTTGATGTTTTAG---------GTCAAAATATTAGGCAATA      92
pro13b2         ATGCAGCGCCACTGCGCAGTTTGATGTTTTAG---------GTCAAAATATTAGGCAATA      155
pro13b3         ATGCAGCGCCACAGCGCAGTTTGATGTTTTAG---------GTCAAAATATTAGGCAATA      153
pro13b4         ATGCAGCGCCACTGCGCAGTTTGATGTTTTAG---------GTCAAAATATTAGGCAATA      153
pro13b22        ATGCAGCGCCTCTGCGCAGTTTGATGTTTTAG---------GTCAAAGTTATAGGCAATA      148
pro13b21        ATGCAGCGCCTCTGCGCAGTTTGATGTTTTAG---------GTCAAAGTTATAGGCAATA      152
pro13b19        ATGCAGCGCCTCTGCGCAGTTTGATGTTTTAG---------GTCAAAGTTATAGGCAATA      151
pro13b20        AAACAGCGCCTCTGCGCAGTTTGATGTTTTAG---------GTCAAAGTTATAGGCAATA      152
pro13b6         ATGCAGCGACTCTGCGCAGTTTGATGTTTTAG---------GTCAAAGTTATAGGCAATA      83
pro13b5         ATGCAACACCTCTGCGTAGTTTGATGTTTTAG---------GTCAAAGTTATAGGCAATA      142
pro13b7         ATGCAGTGCCTCTGCACAGTTTGATGTTTTAG---------GTCAAAGTTATGGGCAATA      150
pro13b18        ATGCAGCGCCTCTGCGCAGTTTGATGTTTTGG---------GTCAAAGTTATAGGCAATA      115
pro13b14        ATGCAGCGCCTCTGCGCAGTTTGATGTTTTAT---------GTCAAAGTTATAGGCAATA      150
pro13b8         ATGCAGCGCCTCTGCGCAGTTTGATGTTTTAG---------GTCAAAGTTATAGGCAATA      154
pro13b17        ATGCAGCGCCTCTGCGCAGTTTGATGTTTTAG---------GTCTAAGTTATAGGCAATA      150
pro13b16        ATGCAGCGCCTCTGCACAGTTTCATGTTTTAG---------GTCAAAGTTATAGGCAATA      150
pro13b15        ATGCAGCGCCTCTGCACAGTTTGATGTTTTAG---------GTCAAAGTTATAGGCAATA      124
pro13b13        ATGCAGCGCCTCTGCGCAGTTTGATGTTTTAG---------GTCAAAGTTATAGGCAATA      130
pro13b9         ATGCAGCGCCTCTGCGCAGTTTGATGTTTTAG---------GTCAAAGTTATAGGCAATA      150
pro13b10        ATGCAGCGCCTCTGCGCAGTTTGATGTTTTAG---------GTCAAAGTTATAGGCAATA      141
pro13b11        ATGCAGCGCCTCTGCGCAGTTTGATGTTTTAG---------GTCAAAGTTATAGGCAATA      150
pro13b12        ATGCAGCGCCTCTGCGCAGTTTGATGTTTTAG---------GTCAAAGTTATAGGCAATA      150
pro13a1         ATGCAATGCCTCTGCGCGGTTTGATCCTCTTA---------GTCAAAGTTATAGGCAATA      154
pro13a2         ATGCAACGCTTCTGCACGGTTTGATGCTCTTA---------GTCAAAGTTATAGACAATA      150
pro13a5         AGGCTT---TCACACGGATTTTAATTATTCTAGGCATAATTTCGGGATGTATTTTAGAGT      471
pro13a3         ATGCAGCGCCTCTACGCAGTTTGATGCTGTT---------ACTCATGTTTACAGGCAATA      149
pro13a4         ATGCAGTGCCTCTGCGCAGTTTGATGCTGTT---------ACTCAAGTTTACAGGCAATA      152
pro13a6         ATGCAGCGCCTCTGCGCAGTTTGATGCTGTT---------ACTCAAGTTTATAGGCAATA      92
                *  *          *    *** **  *                             *
```

```
pro13b1         TCAGGTGCAGTCGCCTCTCCTGCTACAGCAACAGGTGCTTAGCCCATATAATGAGTTCGT      152
pro13b2         TCAGGTGCAGTCGCCTCTCCTGCTACAGCAACAGGTGCTTAGCCCATATAATGAGTTCGT      215
pro13b3         TCAGGTGCAGTCGCCTCTCCTGCTACAGCAACAGGTGCTTAGCCTATATAATGAGTTCGT      213
pro13b4         TCAGGTGCAGTCGCCTCTCCTGCTACAGCAACAGGTGCTTAGCCTATATAATGAGTTCGT      213
pro13b22        TCAGCTGCAGTCACCTCTCCTGCTACAGCAACATGTGCTTAGCCCATATAATGAGTTCGT      208
pro13b21        TCAGCTGCAGTCGCCTCTCCTGCTACAGCAACAGGTGCTTAGCCCATATAATGAGTTCGT      212
pro13b19        TTAGCTGCAGTCGCCTCTCCTGCTACAGCAACAGGTGCTTAGCCCATATAATGAGTTCGT      211
pro13b20        TCAGCTGCAGTCGCCTCTCCTACTACAGCAACATGTGCTTAGCCCATATAATGAGTTCGT      212
pro13b6         TCAGCTGCAGTCGCCTCTCCTGCTACAGCAACAGGTGTTTAGCCCATATAATGAGTTCGT      143
pro13b5         TCAGCTACAGTCGCCTCTCCTACAACAACAACAGGTGCTTAGCCCATATAATGACTTCGT      202
pro13b7         TCTGCTACAGTCGCCTCTCCTGATACAGCAACAGGTGCTTAGCCCATATAATGAGTTCGT      210
pro13b18        TCAGCTGCAGTCGCCTGTCCTACTACAGCAACAGGTGCTTAGCCCATATAATGAGTTCGT      175
pro13b14        TCAGCTGCAGTCGCCTGTCCTGCTACAGCAACAGGTGCTTAGCCCATATAATGAGTTCGT      210
pro13b8         TCAGCTGCAGTCGCCTGTCCTGCTACAGCAACATGTGCTTAGCCCATATAATGAGTTCGT      214
pro13b17        TCAGCTGCAGTCGCCTGTCCTGCTACAGCAACAGGTGCTTAGCCCATATAATGAGTTCGT      210
pro13b16        TCAGCTGCAGTCGCCTGTCCTGCTATAGCAACAGGTGCTTAGCCCATATAATGAGTTCGT      210
pro13b15        TCAGCTGCAGTCGCCTGTCCTGCTACAGCAACAGGTGCTTAGCCCATATAATGAGTTCGT      184
pro13b13        TCAGCTGCAGTCGCCTGTCCTGCTACAGCAACAGGTGCTTAGCCCATATAATGAGTTCGT      190
pro13b9         TCAGCTGCAGTCGCCTGTCCTGCTACAGCAACAGGTGCTTAGCCCATATAATGAGTTCGT      210
pro13b10        TCAGCTGCAGTCGCCTGTCCTGCTACAGCAACAGGTGCTTAGCCCATATAATGAGTTCGT      201
pro13b11        TCAGCTGCAGTCGCCTGTCCTGCTACAGCAACAGGTGCTTAGCCCATATAATGAGTTCGT      210
pro13b12        TCAGCTGCAGTCGCCTGTCCTGCTACAGCAACAGGTGCTTAGCCCATATAATGAGTTCGT      210
pro13a1         TCAACTACAGTCGCATCTCCTACTACAGCAACAAGTGCTCAGCCCATGCAGTGAGTTCGT      214
pro13a2         TCAACTACAATCGCATCTCCTGCTACAGCAACAAGTGCTCAGCCCATGCAGTGAGTTCGT      210
pro13a5         CGTTTTGGGACGCGACATCATGCTGCAGCAACAGATGCTTAGCCCAAGCGGTGAGTTCGT      531
pro13a3         TCAGCTGCAGCCGCATCTCATGCTGCAGCAACAGATGCTTAGCCCATGCGGTGAGTTCGT      209
pro13a4         TCAGCTGCAGCCGCATCTCATGCTGCAGCAACAGATGCTTAGCCCATGCGGTGAGTTCGT      212
pro13a6         TCAGCTGCAGCCGCATCTCATGGTGCAGCAACAGATGCTTAGCCCATGCGGTGAGTTCAT      152
                     *           ** *     * *****  ** * **** *     *** *** *


pro13b1         AAGGCAGCAGTATAGCATTGCGGCAAGCACCTTCTTGCAATCAGCTGCGTTTCAACTGAG      212
pro13b2         AAGGCAGCAGTATAGCATTGCGGCAAGCACCTTCTTGCAATCAGCTGCGTTTCAACTGAG      275
pro13b3         AAGGCAGCAGTATAGCATTGCGGCAAGCCCCTTCTTGCAATCAGCTGTGTTTCAACTGAG      273
pro13b4         AAGGCAGCAGTATAGCATTGCGGCAAGCCCCTTCTTGCAATCAGCTGTGTTTCAAGTGAG      273
pro13b22        AAGGCAGCAGTATGGCATAGCAGCAAGCCCCTTATTGCAATCAGCTGCGTTTCAGCTGAA      268
pro13b21        AAGGCAGCAGTATGGCATAACGGCAAGCCCCTTATTGCAATCAGTTGCGTTTCAACTGAG      272
pro13b19        AAGGCAGCAGTATGGCATAGCGGCAAGCCCCTTATTGCAATCAGCTGCGTTTCAACTGAG      271
pro13b20        AAGGCAGCAGTATGGCATAGCGGCAAGCCCCTTATTGCAATCAGCTGCATTTCAACTGAG      272
pro13b6         AATACAACAGTATGGCACAGCGGCAAGCCCCTTCTTGCGATCAGCTGCGTTTCAACTAAG      203
pro13b5         AAGGCAGCGATATGGCATAGCGGCAAGCCCCTTCTTGCAATCAGCTGCGTTTAAACTGAG      262
pro13b7         AAGGCAGCAGTATGGCATTGCGGCAAGCCCCTTCTTGCAATCAGCTGCGTTTCAACTGAG      270
pro13b18        AAGGCAGCAGTATGGCATAGCGGCAAGCCCCTTCTTGCAATCAGCTGCGTTTCAACTGAG      235
pro13b14        AAGGCAGCAGTATGGCATAGCGGCAAGCCCCTTCTTGCAATCAGCTGCGTTTCAACTGAG      270
pro13b8         AAGGCAGCAGTATGGCATAGCGGCAAGCCCCTTCTTGCAATCAGCTGCGTTTCAACTGAG      274
pro13b17        AAGGCAGCAGTATGGCATAGCGGCAAGCCCCTTCTTGCAATCAGCTGCGTTTCAACTGAG      270
pro13b16        AAGGCAGCAGTATGGCATAGCGGCAAGCCCCTTCTTGCAATCAGCTGTGTTTCAACTGAG      270
pro13b15        AAGGCAGCAGTATGGCATAGCGGCAAGCCCCTTCTTGCAATCAGCTGTGTTTCAACTGAG      244
pro13b13        AAGGCAGCAGTATGGCATAGCGGCAAGCCCCTTCTTGCAATCAGCTGCGTTTCAACTGAG      250
pro13b9         AAGGCAGCAGTATGGCATAGCGGCAAGCCCCTTCTTGCAATCAGCTGCGTTTCAACTGAG      270
pro13b10        AAGGCAGCAGTATGGCATAGCGGCAAGCCCCTTCTTGCAATCAGCTGCGTTTCAACTGAG      261
pro13b11        AAGGCAGCAGTATGGCATAGCGGCAAGCCCCTTCTTGCAATCAGCTGCGTTTCAACTGAG      270
pro13b12        AAGGCAGCAGTATGGCATAGCGGCAAGCCCCTTCTTGCAATCAGCTGCGTTTCAACTGAG      270
pro13a1         AAGGCAACAGTATAGCATAGTGGCAACCCCCTTCTGGCAACCAGCTACGTTTCAATTGAT      274
pro13a2         AAGGCAACAGCATAGCATAGTGGCAACCCCCTTCTGGCAACCAGCTACGTTTCAATTGAT      270
pro13a5         AAGGCAGCAGTGCAGCACAGTGGCAACCCCCTTCTTCCAATCACCCGTGTTTCAACTGAG      591
pro13a3         AAGGCAGCAGTGCAGCACAGTGGAAATCCCCTTCTTCCAATCACCCGTGTTTCAGCTGAG      269
pro13a4         AAGGCAGCAGTGCAGCACAGTGGCAACCCCCTTCTTCCAATCACCCGTGTTTCAACTGAG      272
pro13a6         AAGGCAGTAGTGCAGCACAGTGGCAACCCCCTTCTTCCAATCACCCGTGTTTCAACTGAG      212
                **  **        ***     * ** * **** *  * * **      *** *  * *
```

```
pro13b1         AAACAACCAAGTCTTGC---------------AACAGCTCAGGCTGGTGGCGCAACAATC      257
pro13b2         AAACAACCAAGTCTTGC---------------AACAGCTCAGGCTGGTGGCGCAACAATC      320
pro13b3         AAACAACCAAGTCTTGC---------------AACAGCTCAGGCTGGTGGCGCAACAATC      318
pro13b4         AAACAACCAAGTCTTGC---------------AACAGCTCAGGCTGGTGGCGCAACAATC      318
pro13b22        AAACAACCAAGTTTGGC---------------AACAGCTCAGGCTGGTGGCGCAACAGTC      313
pro13b21        AAACAACCAAGTCTGGC---------------AACAGCTTTGGCTGGTGGCGCAACAGTC      317
pro13b19        AAACAACCAAGTCTGGC---------------AACAGCTCAGGGTGGTGGCGCAGCAGTC      316
pro13b20        AAACAACCAAGTCTGGC---------------AACAGCTCAGGCTGGTGGCGCAACAGTC      317
pro13b6         AAACAACCAAGTCTTTG---------------AACAGCTCGGGCTGGTG-CGCAACAATC      247
pro13b5         AAATAACCAAGTCTGGC---------------AACAGCTCGGGCTGGTGGCGCAACAATC      307
pro13b7         AAACAACCAAGTCTGGC---------------AATAACTTGGGCTGGTGGCGCAACAATC      315
pro13b18        AAACAACCAAGTCTGGC---------------AACAGCTCGGGCTGGTGGCGCAACAATC      280
pro13b14        AAACAACCAAGTCTGGC---------------AACAGCTCGCGCTGGTGGCGCAACAATC      315
pro13b8         AAACAACCAAGTCTGGC---------------AACAGCTCGCGCTGGTGGCGCAACAATC      319
pro13b17        AAACAACCAAGTCTGGC---------------AACAGCTCGCGCTGGTGGCGCAACAATC      315
pro13b16        AAACAACCAAGTCTGGC---------------AACAGCTCGCGCTGGTGGCGCAACAATC      315
pro13b15        AAACAACCAAGTCTGGC---------------AACAGCTCGCGCTGGTGGCGCAACAATC      289
pro13b13        AAACAACCAAGTCTGGC---------------AACAGCTCGCGCTGGTGGCGCAACAATC      295
pro13b9         AAACAACCAAGTCTGGC---------------AACAGCTCGCGCTGGTGGCGCAACAATC      315
pro13b10        AAACAACCAAGTCTGGC---------------AACAGCTCGCGCTGGTGGCGCAACAATC      306
pro13b11        AAACAACCAAGTCTGGC---------------AACAGCTCGCGCTGGTGGCGCAACAATC      315
pro13b12        AAACAACCAAGTCTGGC---------------AACAGCTCGCGCTGGTGGCGCAACAATC      315
pro13a1         AAACAACCAAGTCATGCAGCAGCAGTGTTGCCAACAGCTCAGGCTGGTAGCACAACAATC      334
pro13a2         AAACAACCAAGTCATGCAGCAACAGTGTTGCCAACAGCTCAGGCTGGTAGCGCAACAATC      330
pro13a5         AAACTGCCAAGTCATGCAGCAGCAGTGCTGCCAACAGCTCAGGA-GATCGCGCAACAGTC      650
pro13a3         AAACTGCCAAGTCATGCAGCAGCAGTGCTGCCAACAGCTCAGGATGATCGCGCAACAGTC      329
pro13a4         AAACTGCCAAGTCATGCAGCAGCAGTGCTGCCAACAGCTCAGGATGATCGCACAACAGTC      332
pro13a6         AAACTGCCAAGTCATGCAGTAGCAGTGCTGCCAACAGCTCAGGATGATCGCGCAACAGTC      272
                ***   ******                    ** * **   *  * *  * ** ** **

pro13b1         TCACTACCAGGACATTAACGTTGTCCAGGCCATAGCGCACCAGCTACACCTCCAGCAGTT      317
pro13b2         TCACTACCAGGACATTAACGTTGTCCAGGCCATAGCGCACCAGCTACACCTCCAGCAGTT      380
pro13b3         TCACTACCAGGACATTAACGTTGTCCAGGCCATAGCGCAGCAGCTACACCTCCAGCAGTT      378
pro13b4         TCACTACCAGGACATTAACGTTGTCCAGGCCATAGCGCAGCAGCTACACCTACAGCAGTT      378
pro13b22        TCACTATCAAGATATTAACATTGTTCAGGCCATAGCGCAGCAGCTACAACTCCAGCAGTT      373
pro13b21        TCACTATGAGGATATTAACATTTTTCAGGTCATAGCGCAGCAGCTACAACTCCAGCAGTT      377
pro13b19        TCACTATCAAGGTATTAACATTGTTCAGGCCATAGCGCAGCAGCTACAACTCCAGCAGTT      376
pro13b20        TCACTATCAGGATATTAACATTGTTCAGGCCATAGCGCAACAGCTACAACTCCAGCAGTT      377
pro13b6         TCACTATCAGGACATTAACATTGTTCAGGCCATAG-------------------------      282
pro13b5         TCACTATCAGGACATTAACATTGTTCAGGCCATAGCGCAGCAGCTATAACTCCAGCAGTT      367
pro13b7         TCTCTATCAGGACATTAACATTGTTCAGGCCATAGCACAGCAGCTACAACTCCAGCAGTT      375
pro13b18        TCACTATCAGGACATTAACATTGTTCAGGCCATAGTGCAGCAGCTACAACTCCAGCAGTT      340
pro13b14        TCACTATCAGGACATAAACATTGTTCAGGCCATAGCGCAGCAGCTACAACTCCAGCAGTT      375
pro13b8         TCACTATCAGGACATTAACATTGTTCAGGCCATAGCGCAGCAGCTACAACTCCAGCAGTT      379
pro13b17        TCACTATCAGGACATTAACATTGTTCAGGCCATAGCGCAGCAGCTACAACTCCAGCAGTT      375
pro13b16        TCACTATCAGGACATTAACATTGTTCAGGCCATAGCGCAGCAGCTACAACTCCAGCAGTT      375
pro13b15        TCACTATCAGGACATTAACATTGTTCAGGCCATAGCGCAGCAGCTACAACTCCAGCAGTT      349
pro13b13        TCACTATCAGGACATTAACATTGTTCAGGCCATAGCGCAGCAGCTACAACTCCAGCAGTT      355
pro13b9         TCACTATCAGGACATTAACATTGTTCAGGCCATAGCGCAGCAGCTACAACTCCAGTAGTT      375
pro13b10        TCACTATCAGGACATTAACATTGTTCAGGCCATAGCGCAGCAGCTACAACTCCAGTAGTT      366
pro13b11        TCACTATCAGGACATTAACATTGTTCAGGCCATAGCGCAGCAGCTACAACTCCAGCAGTT      375
pro13b12        TCACTATCAGGACATTAACATTGTTCAGGCCATAGCGCAGCAGCTACAACTCCAGCAGTT      375
pro13a1         TCACTACCAGGCCATTAGTATTGTTCAAGCGATTGTGCAACAGCTACAACTGCAGCAATT      394
pro13a2         TCACTACCAGGCCATTAGTAGCGTTCAGGCGATTGTGCAGCAACTACAGCTGCAGCAGGT      390
pro13a5         TCACTGCCAGGCCATTAGCAGTGTTCAGGCGATTGTGCAGCAGCTACAGCTACAACAGTT      710
pro13a3         TCACTGCCAGGCCGTTAGCAGTGTTCAGGTGATTGTGCAGCAGCTACAGCTACAACAGTT      389
pro13a4         TCACTGCCAGGCCATTAGCAGTGTTCAGGCTATTGTGCAGCAGCTACGGCTACAACAGTT      392
pro13a6         TCACTGCCAGGCCATTAGCAGTGTTCTGGCGATTGTGCAGCAGCTACAGCTACAACAGTT      332
                ** **   * *   * *      * *  *  ** *
```

```
pro13b1      TGGCAATCTCTACATTGACCGGAATCTGGCTCAAG------CTCAAGCACTGTTGGCTTT     371
pro13b2      TGGCAATCTCTACATTGACCGGAATCTGGCTCAAG------CTCAAGCACTGTTGGCTTT     434
pro13b3      TGGCGATCTCTACATTGACCGGAATCTGGCTCAAG------CGCAAGCACTGTTGGCTTT     432
pro13b4      TGGCGATCTCTACATTGACCGGAATCTGGCTCAAG------CTCAAGCACTGTTGGCTTT     432
pro13b22     TGATGATCTCTACTTTGATCGGAATCTGGGTCAAGCTCAAGCTCAAGCTCTGTTGGCTTT     433
pro13b21     TGATGATCTCTACTTTGATCGGAATCTGGCTCAAGCTCCAGCCCAAGCTCTGTTGGCTTT     437
pro13b19     TGATGATCTCTACTTTGATCGGAATCTGGCTCAAGCTCAAGCTCAAGCTCTGTTGGCTTT     436
pro13b20     TGATGATCTCTACTTTGGTCGGAATCTGGCTCAAGCTCAAGCTCAAGCTCTGTTGGCTTT     437
pro13b6      ------------------------------------------------------------     282
pro13b5      TGGTGATCTCTACTTTGATCGGAATCCGGCTCAAG------CTCAAGCTCTGTTGGCTTT     421
pro13b7      TGGTGATCTCTACTTTGATCGGAATCTGGCTCAAG------CTCAAGCTCTGTTGGCTTC     429
pro13b18     TGGTGATCTCTACTTTGATCGGAATCTGGCTCAAG------CTCAAGCTCTGTTGGCTTT     394
pro13b14     TGGTGATCTCTACTTTGATCGGAATCTGGCTCAAG------CTCAAGCTCTGTTGGCTTT     429
pro13b8      TGGTGATCTCTACTTTGATCGGAATCTGGCTCAAG------CTCAAGCTCTGTTGGCTTT     433
pro13b17     TGGTGATCTCTACTTTGATCGGAATCTGGCTCAAG------CTCAAGCTCCGTTGGCTTT     429
pro13b16     TGGTGATCTCTACTTTGATCGGAATCTGGCTCAAG------CTCAAGCTCTGTTGGCTTT     429
pro13b15     TGGTGATCTCTACTTTGATCGGAATCTGGCTCAAG------CTCAAGCTCTGTTGGCTTT     403
pro13b13     TGGTGATCTCTACTTTGATCGGAATCTGGCTCAAG------CTCAAGCTCTGTTGGCTTT     409
pro13b9      TGGTGATCTCTACTTTGATCGGAATCTGGCTCAAG------CTCAAGCTCTGTTGGCTTT     429
pro13b10     TGGTGATCTCTACTTTGATCGGAATCTGGCTCAAG------CTCAAGCTCTGTTGGCTTT     420
pro13b11     TGGTGATCTCTACTTTGATCGGAATCTGGCTCAAG------CTCAAGCTCTGTTGGCTTT     429
pro13b12     TGGTGATCTCTACTTTGATCGGAATCTGGCTCAAG------CTCAAGCTCTGTTGGCTTT     429
pro13a1      TAGTGGTGTCTACTTTGATCAGACTCAAGCTCAAG------CCCAAACTCTGTTGACCTT     448
pro13a2      CGGTGTTGTCTACTTTGATCAGACTCAAGCTCAAG------CTCAAGCTTTGCTGGCCTT     444
pro13a5      T-GTGGCGTCTACTTCGATCAGACTCAAGCTCAAG------CCCAAGCTATGTTGGCCCT     763
pro13a3      TACTGGCATCAACTTCGATCAGACTCAAGCTCTAG------CCCAAGCTATGTTGGCCCT     443
pro13a4      TGCTAGCGTCTACTTCGATCAGAGTCAAGCTCAAG------CCCAAGCTATGTTGGCCCT     446
pro13a6      TGCTGGCGTCTACTTCGATCAGACTCAAGCTCAAG------CCCAAGCTATGTTGGCCCT     386


pro13b1      TAACTTGCCATCTACATATGGTATCTACCCTTGGTCCTATAGTGCACCTGGTAGCATTAC     431
pro13b2      TAACTTGCCATCTACATATGGTATCTACCCTTGGTCCTATAGTGCACCCGATAGCATTAC     494
pro13b3      TAACTTGCCATCTACATATGGTATCTACCCTAGGTACTATAGTGCACCGGGTAGTATTAC     492
pro13b4      TAACTTGCCATCTACATATGGTATCTACCCTAGGTACTATAGTGCACCCGGTAGCATTAC     492
pro13b22     GAACTTGCCATCTAGATATGGTATCTACCCTAGGTACTATAGTGCACCTAGTAGCATTAC     493
pro13b21     GAACTTGCCATCTAGATATGGTATCTACCCTAGGTACTATAGTGCACCTAGTAGCATTAC     497
pro13b19     GAACTTGCCATCTAGATATGGTATCTACCCTAGGTACTATAGTGCACCTAGTAGCATTAC     496
pro13b20     GAACTTGCCATCTAGATATGGTATCTACCCTAGGTACTATAGTGCACCTAGTAGCATTAC     497
pro13b6      ------------------------------------------------------------     282
pro13b5      TAACGTGCCATCTAGATATGGTATCTACCCTAGGTACTATAGTACACCCAGTACCATTAC     481
pro13b7      TAACGTGCCATCTAAATATAGGATATACCCTAGGTACTATGGTGCACCCAGTACCATTAC     489
pro13b18     TAACGTGCCATCTAGATATGGTATCTACCCTAGGTACTATGGTGCACCCAGTACCATTAC     454
pro13b14     TAATGTGCCATCTAGATATGGTATCTACCCTAG---------------------------     462
pro13b8      TAACGTGCCATCTAGATATGGTATCTACCCTAGGTACTATGGTGCACCCAGTACCATTAC     493
pro13b17     TAACGTGccatctag---------------------------------------------     444
pro13b16     TAACGTGCCATCTAGATATGGTATCTACCCTAGGTACTATGGTGCACCCAGTACCATTAC     489
pro13b15     TAACGTGCCATCTAGATATGGTATCTACCCTAGGTACTATGGTGCACCCAGTACCATTAC     463
pro13b13     TAACGTGCCATCTAGATATGGTATCTACCCTAGGTACTATGGTGCACCCAGTACCATTAC     469
pro13b9      TAACGTGCCATCTAGATATGGTATCTACCCTAGGTACTATGGTGCACCCAGTACCATTAC     489
pro13b10     TAACGTGCCATCTAGATATGGTATCTACCCTAGGTACTATGGTGCACCCAGTACCATTAC     480
pro13b11     TAACGTGCCATCTAGATATGGTATCTACCCTAGGTACTATGGTGCACCCAGTACCATTAC     489
pro13b12     TAACGTGCCATCTAGATATGGTATCTACCCTAGGTACTATGGTGCACCCAGTACCATTAC     489
pro13a1      CAACTTGCCATCCATATGTGGTATCTACCCTAACTACTATAGTGCTCCCAGGAGCATTGC     508
pro13a2      AAACTTGCCATCCATATGTGGTATCTATCCTAACTACTACATTGCTCCGAGGAGCATTCC     504
pro13a5      AAACTTGCCGTCAATATGCGGTATCTCC-CAAGCTACAACACTGCTCCCTATAGCATTCC     822
pro13a3      AAACTTGCCGTTAATATGCAGTATCTACCCAAGCTACAACACTGCTCCCTGTAGCATTCG     503
pro13a4      AAACATGCCGTCAATATGCGGTATCTACCCAAGCTACAACACTGCTCCCTGTAGCATTCC     506
pro13a6      AAAGTTGCCGTCAATATGCGTTATCTACCCAAGCTACAACACTGCTCCCTGTAG------     440
```

```
pro13b1     CACCCTTGGCGGTGTCTTGTACTGAATTTTCACAATATTGTAGTTCGGAAGTGAAAATAT    491
pro13b2     CACCCTTGGCGGTGTCTTGTACTGAATTTTCACAATATTGTAGTTCGGAAGTGAAAATAT    554
pro13b3     CACCCTTGGCGGTGTCTTGTACTGAATTTTCACAATATTGTAGTTCGGAAGTGAAAATAT    552
pro13b4     CACCCTTGGCGGTGTCTTGTACTGAATTTTCACAATATTGTAGTTCGGAAGTGAAAATAT    552
pro13b22    CACCCTTGGCGGTGTATTGTACTGAGTTTTAACATTACAGTGGTTCGGAAGTTAAAAA-T    552
pro13b21    CACCCTTGGCGGTTTATTGTACTGAGTTTTAACAATATAGTGGTTTGGAAGTTAAAAA-T    556
pro13b19    CACCCTTGGCGGTGTATTGTACTGAGTTTTAACAATATAGTGGTTCGGAAGTTAAAAA-T    555
pro13b20    CACCCTTGGCGGTGTATTGTACTGAGTTTTAACAATATAGTGGTTTGGAAGTTAAAAA-T    556
pro13b6     ------------------------------------------------------------    282
pro13b5     CACCCTTGGCGGTGTCTTGTAATGAGTTTTAACAGTATAGTGGTTCGGAAGTTAAAAA-T    540
pro13b7     CACCCTTGGCAGTGTCTTGTAATGAGTTTTAACAGTATAGTGGTTCGGAAGTTAAAAA-T    548
pro13b18    CACCCTTGGCGGTGTCTTGTAATGAGTTTTAATAGTATAGTGGTTCGGAAGTTAAAAA-T    513
pro13b14    ------------------------------------------------------------    462
pro13b8     CACCCTTGGCGGTGTCTTGTAATGAGTTTTAACAGTATAGTGGTTCGGAAGATAAAAA-T    552
pro13b17    ------------------------------------------------------------    444
pro13b16    CACCCTTGGCGGTGTCTTGTAATGAGTTTTAACAGTATAGTGGTTCGGAAGTTAAAAA-T    548
pro13b15    CACCCTTGGCGGTGTCTTGTAATGAGTTTTAACAGTATAGTGGTTCGGAAGTTAAAAA-T    522
pro13b13    CACCCTTGGCGGTGTCTTGTAATGAGTTTTAACAGTATAGTGGTTCGGAAGTTAAAAA-T    528
pro13b9     CACCCTTGGCGGTGTCTTGTAATGAGTTTTAACAGTATAGTGGTTCGGAAGTTAAAAA-T    548
pro13b10    CACCCTTGGCGGTGTCTTGTAATGAGTTTTAACAGTATAGTGGTTCGGAAGTTAAAAA-T    539
pro13b11    CACCCTTGGCGGTGTCTTGTAATGAGTTTTAACAGTATAGTGGTTCGGAAGTTAAAAA-T    548
pro13b12    CACCCTTGGCGGTGTCTTGTAATGAGTTTTAACAGTATAGTGGTTCGGAAGTTAAAAA-T    548
pro13a1     CACTGTTGGTGGTGTCTGGTACTGAATTGTAACAATATAATAGTTCGTATGTTAAAAA-T    567
pro13a2     CACCGTTGGTGGTGTCTGGTACTGAATTGTAATAGTATAATGGTTCAAATGTTAAAAA-T    563
pro13a5     CACCGTCGGTAGTATCTGGTACTGAATTGTAGCAGTATAGTAGTACAGGAGAGAAAAA-T    881
pro13a3     CACGGTCGGTGGTATCTGGTACTGAATTGTAGCAGTATAGTAGTACAGGAGAGAGTAA-T    562
pro13a4     CACCGTCGGTGGTATCTGGTATTGAATTGTAGCAGTATAGTAGTACAGGAGAGAAAAA-T    565
pro13a6     ------------------------------------------------------------    440


pro13b1     AAGCTCAGGTATCATCGTATG--TGACATGTGAAACTTGAGGTGATATAAATAGAAATAA    549
pro13b2     AAGCTCAGGTATCATCGTATG--TGACATGTGAAACTTGAGGTGATATAAATAGAAATAA    612
pro13b3     AAGCTCAGGTATCATCGTATG--TGACATGTGAAACTTAAGGTGATATAAATAGAAATAA    610
pro13b4     AAGCTCAGGTATCATCGTATG--TGACATGTGAAACTTAAGGTGATATAAATAGAAATAA    610
pro13b22    AAACTCAGATATCATCATAT--TTGACATGTGAAACTCAGGGTGATATATCCTAGTACAT    610
pro13b21    AGGCTCAGATATCATCATAT--TTGACATGTGAAACTCGGGGTGATATATCCTAGTACAT    614
pro13b19    AAGCTCATATATCATCATATATTT------------------------------------    579
pro13b20    AAGCTCAGATATCATCATATATTTGACATGAGAAACTCGGTGTGATATATCCTTGTACAT    616
pro13b6     ------------------------------------------------------------    282
pro13b5     AAGCTCATATATTATCATATGTGACATG--TGAAATTTGGGGTGAAATAAATAGAAATAA    598
pro13b7     AAGCTCAGATATCATCATATGTGACATG--TGAAACTTTGGGTGATATAAATAGAAATAA    606
pro13b18    AAGCTCAGATATCATCATATGTGACATG--TGAAACTTTGGGTGATAT------------    559
pro13b14    ------------------------------------------------------------    462
pro13b8     AAGCTCAGATATCATCATATGTGACATG--TGAAACTTTGGGTGATATAAATAGAAAAAA    610
pro13b17    ------------------------------------------------------------    444
pro13b16    AAGCTCAGATATCATCATATGTGACATG--TGAAACTTTGGGTGATATAAATAGAAAAAA    606
pro13b15    AAGCTCAGATATCATCATATGTGACATG--TGAAACTTTGGGTGATATAAATAGAAAAAA    580
pro13b13    AAGCTCATATATCATCATATGTGACATG--TGAAACTTTGGGTGATATAAATAGAAAAAA    586
pro13b9     AAGCTCAGATATCATCATATGTGACATG--TGAAACTTTGGGTGATATAAATAGAAAAAA    606
pro13b10    AAGCTCAGATATCATCATATGTGACATG--TGAAACTTTGGGTGATATAAATAGAAAAAA    597
pro13b11    AAGCTCAGATATCATCATATGTGACATG--TGAAACTTTGGGTGATATAAATAGAAAAAA    606
pro13b12    AAGCTCAGATATCATCATATGTGACATG--TGAAACTTTGGGTGATATAAATAGAAAAAA    606
pro13a1     AAAGTCATACATCATCATGTGTGACTGTTGAAACTTA--GGGTCATATAAATCTAAATAA    625
pro13a2     AAAGTCATGCATCATCATGCGTGACAGTTGAAACTTG--ATGTCATATAAATCTAAATAA    621
pro13a5     AAAGTCATGCATCATCGTGTGTGACAAGTTGAAACATCGGGGCGATACAAATCTTAATAA    941
pro13a3     AAAGTCATACATCATCGTGTGTGACAAGTTGAAACATCGAGGCGATACAAATCTGAATGA    622
pro13a4     AAAGTCATGCATCATCGTGTGTGACAAGTTGAAACATCGGGGTGATACAAATCTGAATAA    625
pro13a6     ------------------------------------------------------------    440
```

```
pro13b1     AATTATCTTTCATATTT-------------------------------------------    566
pro13b2     AATTATCTTTCATATTTAAATA--------------------------------------    634
pro13b3     AATTATCTTTCATATTTAAATA--------------------------------------    632
pro13b4     AATTAT------------------------------------------------------    616
pro13b22    CATCGTAACTAATTA---CCATCATTGGTACTCTACA-----------------------    644
pro13b21    CGTCGTAACTAATTA---CCATCGTTGGTACTCTACAATTTTACTGTGTGCTTATATTCG    671
pro13b19    ------------------------------------------------------------    579
pro13b20    CGTCGTAACTAATTA---CTATCATTGGTACTCTACAA----------------------    651
pro13b6     ------------------------------------------------------------    282
pro13b5     AGTTGTCTTTCATATTTAAATA--------------------------------------    620
pro13b7     AGTTGTCTTTCATATTT-------------------------------------------    623
pro13b18    ------------------------------------------------------------    559
pro13b14    ------------------------------------------------------------    462
pro13b8     AGTTGTCTTTCATATTTAAATACCATGCCTCTATAA-------------GGATATATCCT    657
pro13b17    ------------------------------------------------------------    444
pro13b16    AGTTGTCTTTCATATTT-------------------------------------------    623
pro13b15    AGTTGTCTTTC-------------------------------------------------    591
pro13b13    AGTTGTCTTTCATATTT-------------------------------------------    603
pro13b9     AGTTGTCTTTCATATTT-------------------------------------------    623
pro13b10    AGTTGTCTTTC-------------------------------------------------    608
pro13b11    AGTTGTCTTTCATATTT-------------------------------------------    623
pro13b12    AGTTGTCTTTCATATTT-------------------------------------------    623
pro13a1     AATCATCTTACCTATTTAAATAGCATTCCTCTATGAGGATAAATCCTATTACATTATCGT    685
pro13a2     AATCACCTA-----TTTAAATAGCATTCATGTATGAGGTTGCATT----------ATCAT    666
pro13a5     AAATGTCATGCAAATTTAAACATAATGCCTCTGTAAGGATAAATCCTAGTACATTGTTGA    1001
pro13a3     AAATGTCATGCAAGTTTAAACATAATGCCTCTGTAAGGATAAATCCTAGTACATTGTTGA    682
pro13a4     AAATGTCATGCAAGTTTAAACATAATGCCTCTGTAAGGATAAATCCTAGTACATTGTTGA    685
pro13a6     ------------------------------------------------------------    440


pro13b1     ------------------------------------------------------------    566
pro13b2     ------------------------------------------------------------    634
pro13b3     ------------------------------------------------------------    632
pro13b4     ------------------------------------------------------------    616
pro13b22    ------------------------------------------------------------    644
pro13b21    ----------------------------------------------------ATCCGAAG    679
pro13b19    ------------------------------------------------------------    579
pro13b20    ------------------------------------------------------------    651
pro13b6     ------------------------------------------------------------    282
pro13b5     ------------------------------------------------------------    620
pro13b7     ------------------------------------------------------------    623
pro13b18    ------------------------------------------------------------    559
pro13b14    ------------------------------------------------------------    462
pro13b8     ----------------------------------------------------AGT--A--    661
pro13b17    ------------------------------------------------------------    444
pro13b16    ------------------------------------------------------------    623
pro13b15    ------------------------------------------------------------    591
pro13b13    ------------------------------------------------------------    603
pro13b9     ------------------------------------------------------------    623
pro13b10    ------------------------------------------------------------    608
pro13b11    ------------------------------------------------------------    623
pro13b12    ------------------------------------------------------------    623
pro13a1     AGCTAATTACCATCACAGGGACTTTACAATTTTACTATATGCATGCATTTG-ATCCTAAG    744
pro13a2     AGCTAATTACCATCACAAAGAACTTTAC--------------------------------    694
pro13a5     AATTAATTATCATCACCGCCTATGGT---TGCATTTACAACTTTACGATGTGTTCCTACA    1058
pro13a3     AATTAATTATCATC----------------------------------------------    696
pro13a4     AATTAATTATCGTCACCGTCTACTATGGTTGCATTTACAACTTTA---------------    730
pro13a6     ------------------------------------------------------------    440
```

```
pro13b1     ------------------------------------------------------------     566
pro13b2     ------------------------------------------------------------     634
pro13b3     ------------------------------------------------------------     632
pro13b4     ------------------------------------------------------------     616
pro13b22    ------------------------------------------------------------     644
pro13b21    CTACTTTGTCTTTAAGATATAAATGGAGCGTATAAAGGATGTCCATCCTTTAATTCCAAT     739
pro13b19    ------------------------------------------------------------     579
pro13b20    ------------------------------------------------------------     651
pro13b6     ------------------------------------------------------------     282
pro13b5     ------------------------------------------------------------     620
pro13b7     ------------------------------------------------------------     623
pro13b18    ------------------------------------------------------------     559
pro13b14    ------------------------------------------------------------     462
pro13b8     ---CGTTGTCGTGACT--------------------------------------------     674
pro13b17    ------------------------------------------------------------     444
pro13b16    ------------------------------------------------------------     623
pro13b15    ------------------------------------------------------------     591
pro13b13    ------------------------------------------------------------     603
pro13b9     ------------------------------------------------------------     623
pro13b10    ------------------------------------------------------------     608
pro13b11    ------------------------------------------------------------     623
pro13b12    ------------------------------------------------------------     623
pro13a1     CTGCTTTGGCTATTAGATATAAATGAAGTGCATTAAGCAAGTTCAACCTTTCATTCCCAA     804
pro13a2     ------------------------------------------------------------     694
pro13a5     ----TTTGGTTCTAAGCTA-----------------------------------------     1073
pro13a3     ------------------------------------------------------------     696
pro13a4     ------------------------------------------------------------     730
pro13a6     ------------------------------------------------------------     440
```

```
pro13b1     ------------------------------------------------------------     566
pro13b2     ------------------------------------------------------------     634
pro13b3     ------------------------------------------------------------     632
pro13b4     ------------------------------------------------------------     616
pro13b22    ------------------------------------------------------------     644
pro13b21    AAGAA-------------------------------------------------------     744
pro13b19    ------------------------------------------------------------     579
pro13b20    ------------------------------------------------------------     651
pro13b6     ------------------------------------------------------------     282
pro13b5     ------------------------------------------------------------     620
pro13b7     ------------------------------------------------------------     623
pro13b18    ------------------------------------------------------------     559
pro13b14    ------------------------------------------------------------     462
pro13b8     ------------------------------------------------------------     674
pro13b17    ------------------------------------------------------------     444
pro13b16    ------------------------------------------------------------     623
pro13b15    ------------------------------------------------------------     591
pro13b13    ------------------------------------------------------------     603
pro13b9     ------------------------------------------------------------     623
pro13b10    ------------------------------------------------------------     608
pro13b11    ------------------------------------------------------------     623
pro13b12    ------------------------------------------------------------     623
pro13a1     TAGGAACGAACTTGAAGCACAGATGTCAATTACTATACCCCTAACAATTGATACACTATA     864
pro13a2     ------------------------------------------------------------     694
pro13a5     ------------------------------------------------------------     1073
pro13a3     ------------------------------------------------------------     696
pro13a4     ------------------------------------------------------------     730
pro13a6     ------------------------------------------------------------     440
```

```
pro13b1         ------------------------------------------------------------      566
pro13b2         ------------------------------------------------------------      634
pro13b3         ------------------------------------------------------------      632
pro13b4         ------------------------------------------------------------      616
pro13b22        ------------------------------------------------------------      644
pro13b21        ------------------------------------------------------------      744
pro13b19        ------------------------------------------------------------      579
pro13b20        ------------------------------------------------------------      651
pro13b6         ------------------------------------------------------------      282
pro13b5         ------------------------------------------------------------      620
pro13b7         ------------------------------------------------------------      623
pro13b18        ------------------------------------------------------------      559
pro13b14        ------------------------------------------------------------      462
pro13b8         ------------------------------------------------------------      674
pro13b17        ------------------------------------------------------------      444
pro13b16        ------------------------------------------------------------      623
pro13b15        ------------------------------------------------------------      591
pro13b13        ------------------------------------------------------------      603
pro13b9         ------------------------------------------------------------      623
pro13b10        ------------------------------------------------------------      608
pro13b11        ------------------------------------------------------------      623
pro13b12        ------------------------------------------------------------      623
pro13a1         GCAGCGGTTGTAGCCGCAGCTGGAGGATTCGCTGTCGAGCAGACCATATACCTGAATTTA      924
pro13a2         ------------------------------------------------------------      694
pro13a5         ------------------------------------------------------------      1073
pro13a3         ------------------------------------------------------------      696
pro13a4         ------------------------------------------------------------      730
pro13a6         ------------------------------------------------------------      440

pro13b1         ------------------------------------------------  566
pro13b2         ------------------------------------------------  634
pro13b3         ------------------------------------------------  632
pro13b4         ------------------------------------------------  616
pro13b22        ------------------------------------------------  644
pro13b21        ------------------------------------------------  744
pro13b19        ------------------------------------------------  579
pro13b20        ------------------------------------------------  651
pro13b6         ------------------------------------------------  282
pro13b5         ------------------------------------------------  620
pro13b7         ------------------------------------------------  623
pro13b18        ------------------------------------------------  559
pro13b14        ------------------------------------------------  462
pro13b8         ------------------------------------------------  674
pro13b17        ------------------------------------------------  444
pro13b16        ------------------------------------------------  623
pro13b15        ------------------------------------------------  591
pro13b13        ------------------------------------------------  603
pro13b9         ------------------------------------------------  623
pro13b10        ------------------------------------------------  608
pro13b11        ------------------------------------------------  623
pro13b12        ------------------------------------------------  623
pro13a1         AACCATGTCAAATATCCGTAATGAATAACAAATAATTTTATATTGTGC  972
pro13a2         ------------------------------------------------  694
pro13a5         ------------------------------------------------  1073
pro13a3         ------------------------------------------------  696
pro13a4         ------------------------------------------------  730
pro13a6         ------------------------------------------------  440
```
