# Supplementary material for: Compensatory Modulation of Seed Storage Protein Synthesis and Alteration of Starch Accumulation by Selective Editing of 13 kDa Prolamin Genes by CRISPR-Cas9 in Rice
Source: Int J Mol Sci. 2024 Jun 14;25(12):6579. doi: 10.3390/ijms25126579 (PMC11204006; doi:10.3390/ijms25126579)
Supplement: Supplementary file 1 [file ijms-25-06579-s001.zip › Supplementary Figures.zip!/Supplementary Figgure S Legends - 20240605.pdf]

Figure S1. Schematic diagram for designing sgRNAs

Figure S2. Cas9 cleavage assay for checking sgRNA efficiency in vitro. Digesting templates were genomic DNA amplified using the specific primers for each prolamin gene subgroups (Pro13a-I and Pro13b-I/II) and the testing sgRNAs were transcribed *in vitro* (using the Guide-it Complete sgRNA Screening System). Symbol (+) and (-) indicate the presence or absence of sgRNA-Cas9 complex, respectively. S/M: size marker. Fragments of cleaved-Pro13a-I are 210bp and 190bp. Fragments of cleaved Pro13b-I/II are 250bp and 180bp.

Figure S3. Construction of a CRISPR-Cas9 binary vector for editing rice 13 kDa prolamin genes.

Figure S4. Genotype of *13 kDa prolamin*-knockout lines in T_0 generation. (A) Sequence analysis of target genes in T_0 plants showing various indel mutations. Sequences of sgRNAs were marked with underlines, PAM sequence was marked in boxes; dash lines and bold letters represent deletions and insertions of nucleotides, respectively; and '*' represents nucleotides identical to those in the original WT sequences. (B) Transgenicity assay. The gel image represents PCR amplification of 1839-bp *SpCas9* gene and a 615-bp *HygR* gene in the putative T_0 transgenic plants. S/M: size marker.

Figure S5. Transgenicity assay of T_1 plants. The gel image represents PCR amplification of 1839-bp *SpCas9* gene and a 615-bp *HygR* gene in the putative T_1 transgenic plants. S/M: size marker

Figure S6. Regression plot showing strong positive linear relationship between RNA seq data and qRT-PCR data with an R-square > 0.8. The qRT-PCR data is normalized using $2^{-\Delta\Delta CT}$ method and the RNA seq transcript is presented as log_2[foldchange] data. The plot was visualized using SRplot "Pearson spearman scatte" (https://www.bioinformatics.com.cn/plot_basic_pcc_scatter_plot_049_en).

Figure S7. Expression level of SSP genes in *13 kDa prolamin*-knockout lines. (A) Heatmap displays the expression profiles of the SSP genes from RNAseq analysis. Normalized expression of each gene is represented by Z-score. (B) qRT-PCR analysis of SSP genes in *13 kDa prolamin*-knockout lines. Transcripts encoding different SSPs were analyzed in the immature seeds of WT and mutant lines. Normalized expression of the target genes was calculated using the $2^{-\Delta\Delta CT}$ method and is represented as log_2 value. Error bars denote ± SD of three replicates. P values were calculated using the Student's t-test (* p < 0.1, ** p < 0.01 and *** p < 0.001).

Figure S8. Sequence alignment of all the 13 kDa prolamins genes.
