# Supplementary material for: Compensatory Modulation of Seed Storage Protein Synthesis and Alteration of Starch Accumulation by Selective Editing of 13 kDa Prolamin Genes by CRISPR-Cas9 in Rice
Source: Int J Mol Sci. 2024 Jun 14;25(12):6579. doi: 10.3390/ijms25126579 (PMC11204006; doi:10.3390/ijms25126579)
Supplement: Supplementary file 1 [file ijms-25-06579-s001.zip › Supplementary Tables.zip!/Supplementary Tables S Legends - 20240506.pdf]

Table S1. Design of sgRNAs

Table S2. Genotype of *13 kDa prolamin*-knockout lines (T_0 generation)

Table S3. Genotype of *13 kDa prolamin*-knockout lines (T_1 generation)

Table S4. Genotype of *13 kDa prolamin*-knockout lines (T_2 generation)

Table S5. Functional categories of the differentially expressed transcripts in *13 kDa prolamin*-knockout lines in comparison with WT

Table S6. Annotation of genes with the correlation coefficient with 13 kDa prolamin $\geq |0.7|$

Table S7. Correlation with 13 kDa prolamin genes

Table S8. Primer list

Table S9. Primer list used in deep-sequencing
