# Supplementary material for: Compensatory Modulation of Seed Storage Protein Synthesis and Alteration of Starch Accumulation by Selective Editing of 13 kDa Prolamin Genes by CRISPR-Cas9 in Rice
Source: Int J Mol Sci. 2024 Jun 14;25(12):6579. doi: 10.3390/ijms25126579 (PMC11204006; doi:10.3390/ijms25126579)
Supplement: Supplementary file 1 [file ijms-25-06579-s001.zip › Supplementary Tables.zip!/Table S1.pdf]

Table S1. Design of sgRNAs

sgRNAs		Target genes				Positions of sgRNAs on target genes
Name	Sequence (5'→ 3')	Gene name	Accession #	Gene sequence (5'→ 3')*	Target score (%)	
sgRNA_pro13a	AACGTAGCTGGTTGCCAGAA**GGG**	*Pro13a.1*	Os07g0206400	AACGTAGCTGGTTGCCAGAA**GGG**	100.0	
		Pro13a.2	Os07g0206500	AACGTAGCTGGTTGCCAGAA**GGG**	100.0	
sgRNA_pro13b	AAACGCAGCTGATTGCAAGA**AGG**	*Pro13b.1*	Os07g0219250	AAACGCAGCTGATTGCAAGA**AGG**	100.0	
		Pro13b.2	Os07g0219300	AAACGCAGCTGATTGCAAGA**AGG**	100.0	
		Pro13b.3	Os07g0219400	AAACACAGCTGATTGCAAGA**AGG**	100.0	
		Pro13b.4	Os07g0220000	AAACACAGCTGATTGCAAGA**AGG**	100.0	
		Pro13b.5	Os05g0328333	AAACGCAGCTGATTGCAAGA**AGG**	100.0	
		Pro13b.6	Os05g0328466	AAACGCAGCTGATCGCAAGA**AGG**	14.9	
		Pro13b.7	Os05g0328632	AAACGCAGCTGATTGCAAGA**AGG**	100.0	
		Pro13b.8	Os05g0328800	AAACGCAGCTGATTGCAAGA**AGG**	100.0	
		Pro13b.9	Os05g0328901	AAACGCAGCTGATTGCAAGA**AGG**	100.0	
		Pro13b.10	Os05g0329001	AAACGCAGCTGATTGCAAGA**AGG**	100.0	
		Pro13b.11	Os05g0329100	AAACGCAGCTGATTGCAAGA**AGG**	100.0	
		Pro13b.12	Os05g0329300	AAACGCAGCTGATTGCAAGA**AGG**	100.0	
		Pro13b.13	Os05g0329350	AAACGCAGCTGATTGCAAGA**AGG**	100.0	
		Pro13b.14	Os05g0329400	AAACGCAGCTGATTGCAAGA**AGG**	100.0	
		Pro13b.15	Os05g0329700	AAACACAGCTGATTGCAAGA**AGG**	100.0	
		Pro13b.16	Os05g0329200	AAACACAGCTGATTGCAAGA**AGG**	100.0	
		Pro13b.17	Os05g0330150	AAACGCAGCTGATTGCAAGA**AGG**	100.0	
		Pro13b.18	Os05g0330600	AAACGCAGCTGATTGCAAGA**AGG**	100.0	
		Pro13b.19	Os05g0331366	AAACGCAGCTGATTGCAATA**AGG**	31.5	
		Pro13b.20	Os05g0331532	AAATGCAGCTGATTGCAATA**AGG**	4.3	
		Pro13b.21	Os05g0331800	AAACGCAACTGATTGCAATA**AGG**	4.3	
		Pro13b.22	Os05g0332000	AAACGCAGCTGATTGCAATA**AGG**	31.5	

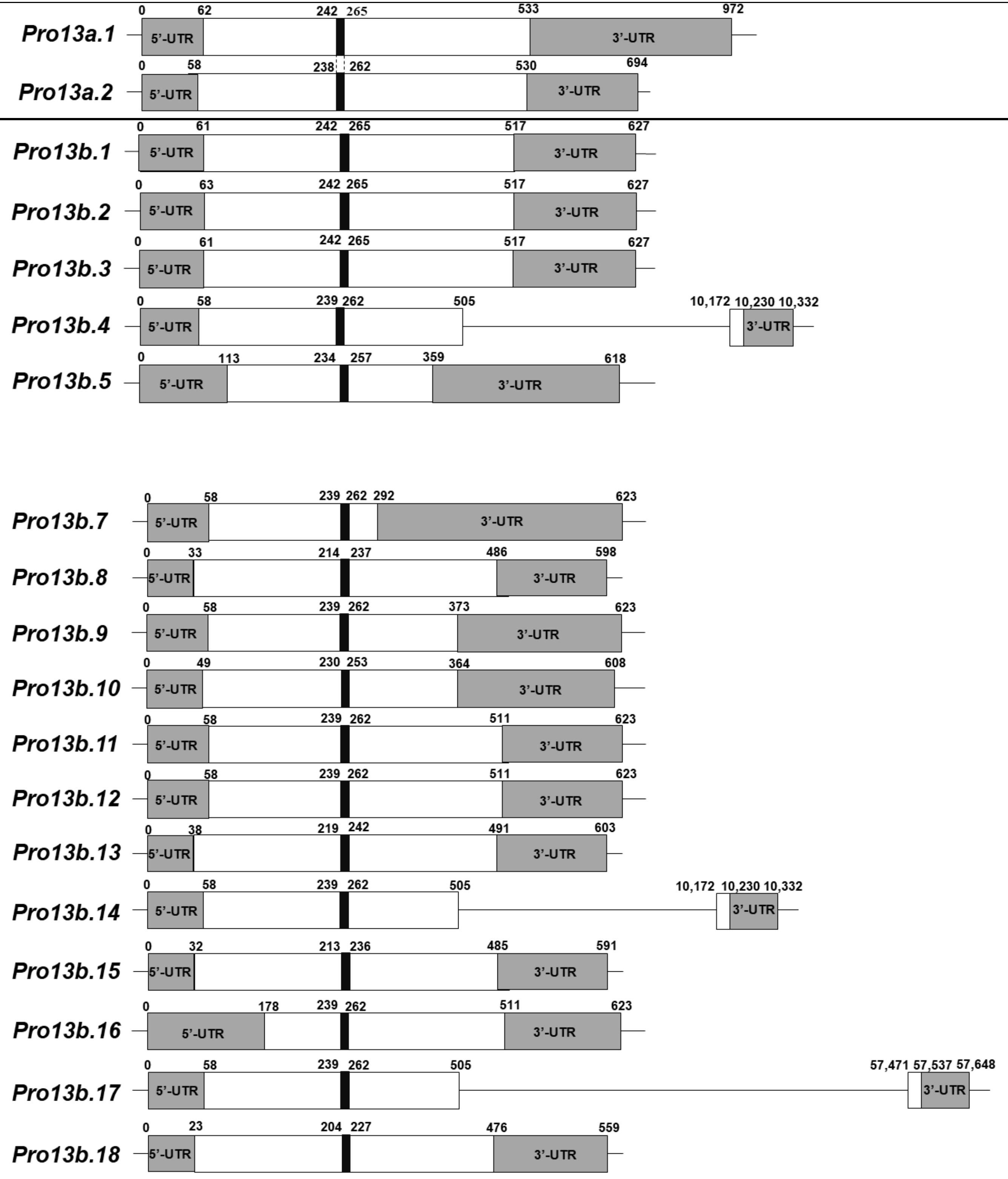

* Red color letters indicate the non-matching nucleotide between sgRNAs and the respective gene sequences
