# Supplementary material for: Compensatory Modulation of Seed Storage Protein Synthesis and Alteration of Starch Accumulation by Selective Editing of 13 kDa Prolamin Genes by CRISPR-Cas9 in Rice
Source: Int J Mol Sci. 2024 Jun 14;25(12):6579. doi: 10.3390/ijms25126579 (PMC11204006; doi:10.3390/ijms25126579)
Supplement: Supplementary file 1 [file ijms-25-06579-s001.zip › Supplementary Tables.zip!/Table S2.pdf]

Table S2 : Genotype of *13 kDa prolamin*-knockout lines (T_0 generation)

**sgRNA**	**T_0 Plants**	**Transgenicity Assay[a]**	**Edited gene**	**Frequency[b]**	**Type**
		SpCas9/Hyg^R			
sgRNA-pro13a	*1a*	+/+	Pro13a-I	51.3%	Ins
	2a	+/+	Pro13a-I	28.5%	Del
	3a	+/+	Pro13a-I	2.3%	Del
	4a	-/-	Pro13a-I	0.0%	WT
	5a	+/+	Pro13a-I	0.1%	WT
	6a	+/+	Pro13a-I	0.0%	WT
	7a	-/-	Pro13a-I	0.0%	WT
	8a	+/+	Pro13a-I	0.0%	WT
	9a	+/+	Pro13a-I	0.0%	WT
	10a	+/+	Pro13a-I	0.4%	WT
sgRNA-pro13b	*1b*	+/+	Pro13b-I/II	0.0%	WT
	2b	+/+	Pro13b-I/II	0.1%	WT
	3b	+/+	Pro13b-I/II	0.2%	WT
	4b	+/+	Pro13b-I/II	4.8%	Ins
	5b	+/+	Pro13b-I/II	0.0%	WT
	6b	+/+	Pro13b-I/II	0.0%	WT
	7b	+/+	Pro13b-I/II	0.0%	WT
	8b	+/+	Pro13b-I/II	12.6%	Del
	9b	+/+	Pro13b-I/II	0.1%	WT
	10b	+/+	Pro13b-I/II	0.0%	WT

[a]Symbol '+' and '-' indicate that SpCas9/Hyg^R genes are present or absent, respectively

[b]Indel (Insertion & Deletion) frequency was calculated from Deep sequencing analysis
