# Supplementary material for: Compensatory Modulation of Seed Storage Protein Synthesis and Alteration of Starch Accumulation by Selective Editing of 13 kDa Prolamin Genes by CRISPR-Cas9 in Rice
Source: Int J Mol Sci. 2024 Jun 14;25(12):6579. doi: 10.3390/ijms25126579 (PMC11204006; doi:10.3390/ijms25126579)
Supplement: Supplementary file 1 [file ijms-25-06579-s001.zip › Supplementary Tables.zip!/Table S3.pdf]

Table S3. Genotype of *13 kDa prolamin*-knockout lines (T_1 generation)

sgRNA	T_1 plants	Transgenicity assay[a] *SpCas9/Hyg[R]*	Edited gene	Frequency[b]	Type	Sequencing results
sgRNA-pro13a	WT	-/-			WT	TTGAAACGTAGCTGGTTGCCA...GAAGGGGGTT
	1a-6	-/-	*Pro13a.1*	99.9%	Ins (1 Nu)	*********************A**********
			Pro13a.2	50.0%	Ins (1 Nu)	*********************A**********
	1a-8	-/-	*Pro13a.1*	99.9%	Ins (1 Nu)	*********************A**********
			Pro13a.2	99.8%	Ins (1 Nu)	*********************A**********
	1a-10	-/-	*Pro13a.1*	99.9%	Ins (1 Nu)	*********************A**********
			Pro13a.2	0.1%	WT	*********************...**********
	1a-11	-/-	*Pro13a.1*	99.9%	Ins (1 Nu)	*********************A**********
			Pro13a.2	48.9%	Ins (1 Nu)	*********************A**********
	2a-1	-/-	*Pro13a.1*	50.6%	Del (1 Nu)	********************-...**********
	2a-2	-/-	*Pro13a.1*	99.8%	Del (1 Nu)	********************-...**********
	2a-6	-/-	*Pro13a.1*	0.0%	WT	*********************...**********
	2a-7	-/-	*Pro13a.1*	50.8%	Del (1 Nu)	********************-...**********
	2a-9	-/-	*Pro13a.1*	52.1%	Del (1 Nu)	********************-...**********
sgRNA-pro13b	WT	-/-			WT	GTTGAAACGCAGCTGATTGCA...AGAAGGTGCT
	4b-1	+/+	*Pro13b.1*	49.3%	Ins (1 Nu)	*********************A**********

4b-2	+/+	*Pro13b.1*	50.6%	Ins (1 Nu)	*********************A**********
4b-3	-/-	*Pro13b.1*	0.0%	WT	*********************...**********
4b-5	+/+	*Pro13b.1*	50.2%	Ins (1 Nu)	*********************A**********
4b-7	+/+	*Pro13b.1*	49.9%	Ins (1 Nu)	*********************A**********
4b-9	-/-	*Pro13b.1*	99.4%	Ins (1 Nu)	*********************A**********
		Pro13b.13	99.8%	Ins (1 Nu)	*********************A**********
4b-10	+/+	*Pro13b.1*	49.4%	Ins (1 Nu)	*********************A**********
8b-3	-/-	*Pro13b.3*	100.0%	Del (4 Nu)	*****************----...**********
8b-6	-/-	*Pro13b.3*	0.1%	WT	*********************...**********
8b-11	-/-	*Pro13b.3*	49.9%	Del (4 Nu)	*****************----...**********

[a]Symbol '+' and '-' indicate that SpCas9/Hyg^R genes are present or absent, respectively

[b]Indel (Insertion & Deletion) frequency was calculated from Deep sequencing analysis

Red color dash and letter indicate the deletion and insertion of nucleotide, respectively

Green letters indicate the PAM sequence

Asterisks indicate the nucleotides with no change
