# Supplementary material for: Compensatory Modulation of Seed Storage Protein Synthesis and Alteration of Starch Accumulation by Selective Editing of 13 kDa Prolamin Genes by CRISPR-Cas9 in Rice
Source: Int J Mol Sci. 2024 Jun 14;25(12):6579. doi: 10.3390/ijms25126579 (PMC11204006; doi:10.3390/ijms25126579)
Supplement: Supplementary file 1 [file ijms-25-06579-s001.zip › Supplementary Tables.zip!/Table S5.pdf]

Table S5.Functional categories of the differentially expressed transcripts in *13 kDa prolamin*-knockout lines in comparison with WT

BIN Code[a]	Annotation	*1a-8-1 vs* Ilmi				*2a-2-1 vs* Ilmi				*4b-9-7 vs* Ilmi				*8b-3-9 vs* Ilmi				Total number of non-redundant genes			
		Up		Down		Up		Down		Up		Down		Up		Down		Up		Down	
		No.	%	No.	%	No.	%	No.	%	No.	%	No.	%	No.	%	No.	%	No.	%	No.	%
1	Photosyntheisis	1	0.37%	4	3.25%	0	0.00%	6	3.37%	0	0.00%	4	4.00%	4	2.29%	5	2.28%	4	1.23%	12	5.24%
2/3/4/7/8[b]	Carbohydrate metabolism-related BINs	4	1.49%	1	0.81%	2	1.03%	2	1.12%	0	0.00%	0	0.00%	0	0.00%	7	3.20%	5	1.53%	7	3.06%
9	Mitochondrial electron transport/ATP synthesis	12	4.48%	0	0.00%	1	0.52%	1	0.56%	0	0.00%	0	0.00%	2	1.14%	2	0.91%	14	4.29%	3	1.31%
10	Cell wall	5	1.87%	1	0.81%	3	1.55%	1	0.56%	1	0.85%	1	1.00%	0	0.00%	1	0.46%	9	2.76%	1	0.44%
11	Lipid metabolism	0	0.00%	2	1.63%	4	2.06%	2	1.12%	0	0.00%	1	1.00%	0	0.00%	1	0.46%	4	1.23%	5	2.18%
12/14/16/18/21/23/24[c]	Other metabolism-related BINs	1	0.37%	2	1.63%	2	1.03%	7	3.93%	2	1.71%	0	0.00%	2	1.14%	7	3.20%	5	1.53%	11	4.80%
13	Amino acid metabolism	1	0.37%	0	0.00%	1	0.52%	1	0.56%	0	0.00%	0	0.00%	3	1.71%	2	0.91%	4	1.23%	2	0.87%
17	Hormone metabolism	2	0.75%	2	1.63%	4	2.06%	1	0.56%	0	0.00%	1	1.00%	4	2.29%	1	0.46%	5	1.53%	3	1.31%
20	Stress	4	1.49%	3	2.44%	11	5.67%	2	1.12%	11	9.40%	0	0.00%	10	5.71%	5	2.28%	23	7.06%	7	3.06%
26	Miscellaneous	13	4.85%	5	4.07%	4	2.06%	7	3.93%	4	3.42%	3	3.00%	4	2.29%	8	3.65%	20	6.13%	15	6.55%
27.1	RNA.processing	40	14.93%	29	23.58%	35	18.04%	37	20.79%	25	21.37%	27	27.00%	31	17.71%	30	13.70%	83	25.46%	69	30.13%
27.2/3	RNA.transcription and RNA.regulation of transcription	5	1.87%	2	1.63%	12	6.19%	5	2.81%	4	3.42%	2	2.00%	7	4.00%	12	5.48%	20	6.13%	17	7.42%
28	DNA synthesis and repair	0	0.00%	1	0.81%	0	0.00%	1	0.56%	0	0.00%	0	0.00%	1	0.57%	2	0.91%	1	0.31%	3	1.31%
29.3/4/5/6[d]	Protein activity regulation-related BINs	7	2.61%	6	4.88%	6	3.09%	9	5.06%	3	2.56%	3	3.00%	2	1.14%	9	4.11%	16	4.91%	18	7.86%
29.2.1/4	Protein synthesis-ribosome and elongation	2	0.75%	2	1.63%	0	0.00%	1	0.56%	1	0.85%	1	1.00%	1	0.57%	2	0.91%	4	1.23%	4	1.75%
29.2.6	Protein synthesis-ribosomal RNA	36	13.43%	0	0.00%	0	0.00%	0	0.00%	2	1.71%	1	1.00%	3	1.71%	0	0.00%	36	11.04%	1	0.44%
29.2.7	Protein synthesis_transfer RNA	8	2.99%	8	6.50%	4	2.06%	7	3.93%	2	1.71%	13	13.00%	4	2.29%	22	10.05%	15	4.60%	25	10.92%
30	Signalling	6	2.24%	0	0.00%	3	1.55%	1	0.56%	2	1.71%	0	0.00%	3	1.71%	4	1.83%	10	3.07%	4	1.75%
31	Cell organization	1	0.37%	1	0.81%	3	1.55%	2	1.12%	1	0.85%	2	2.00%	3	1.71%	3	1.37%	6	1.84%	4	1.75%
33	Development	24	8.96%	3	2.44%	9	4.64%	2	1.12%	4	3.42%	1	1.00%	6	3.43%	3	1.37%	30	9.20%	8	3.49%
34	Transport	8	2.99%	1	0.81%	4	2.06%	4	2.25%	1	0.85%	1	1.00%	4	2.29%	7	3.20%	12	3.68%	10	4.37%
35	Unknown	88	32.84%	50	40.65%	86	44.33%	79	44.38%	54	46.15%	39	39.00%	81	46.29%	86	39.27%	214		174	
Total		268	100.00%	123	100.00%	194	100.00%	178	100.00%	117	100.00%	100	100.00%	175	100.00%	219	100.00%	540	100.00%	403	100.00%

[a] BIN codes of genes were produced according to MapMan classification using the MapCave tool (http://mapman.gabipd.org/web/guest/mapcave).

[b] BIN 2/3/4/7/8 is carbohydrate metabolism-related BINs: major carbohydrates (BIN 2), minor carbohydrates (BIN 3), glycolysis (BIN 4), OPP cycle (BIN 7), and TCA/organic acid transformation (BIN 8)

[c] BIN 12/14/16/18/21/23/24 is the other metabolism-related BINs: nitrogen assimilation (BIN 12), S-assimilation (BIN 14), secondary metabolism (BIN 16), cofactor/vitamin synthesis (BIN 18), redox (BIN 21), nucleotide metabolism (BIN 23), biodegradation of xenobiotics (BIN 24)

[d] BIN 29.3/4/5/6 is protein activity regulation-related BINs including protein targeting (BIN 29.3), protein post-translational modification (BIN 29.4), protein degradation (BIN 29.5), and protein folding (BIN 29.6)
