# Supplementary material for: Compensatory Modulation of Seed Storage Protein Synthesis and Alteration of Starch Accumulation by Selective Editing of 13 kDa Prolamin Genes by CRISPR-Cas9 in Rice
Source: Int J Mol Sci. 2024 Jun 14;25(12):6579. doi: 10.3390/ijms25126579 (PMC11204006; doi:10.3390/ijms25126579)
Supplement: Supplementary file 1 [file ijms-25-06579-s001.zip › Supplementary Tables.zip!/Table S6.pdf]

Table S6. Annotation of genes with the correlation coefficient with 13 kDa prolamin ≥|0.7|

Gene ID	Name	Description	BINcode		Function
ENSRNA049445376	tRNA-Trp	tRNA-Trp for anticodon CCA	29	29.2.7	Protein synthesis_transfer RNA
ENSRNA049445384	tRNA-Trp	tRNA-Trp for anticodon CCA	29	29.2.7	Protein synthesis_transfer RNA
ENSRNA049445795	tRNA-Gln	tRNA-Gln for anticodon CUG	29	29.2.7	Protein synthesis_transfer RNA
ENSRNA049446836	tRNA-Trp	tRNA-Trp for anticodon CCA	29	29.2.7	Protein synthesis_transfer RNA
ENSRNA049447201	tRNA-Trp	tRNA-Trp for anticodon CCA	29	29.2.7	Protein synthesis_transfer RNA
ENSRNA049447972	tRNA-Trp	tRNA-Trp for anticodon CCA	29	29.2.7	Protein synthesis_transfer RNA
ENSRNA049448151	tRNA-Trp	tRNA-Trp for anticodon CCA	29	29.2.7	Protein synthesis_transfer RNA
ENSRNA049449035	tRNA-Trp	tRNA-Trp for anticodon CCA	29	29.2.7	Protein synthesis_transfer RNA
ENSRNA049449070	tRNA-Trp	tRNA-Trp for anticodon CCA	29	29.2.7	Protein synthesis_transfer RNA
ENSRNA049449267	tRNA-Trp	tRNA-Trp for anticodon CCA	29	29.2.7	Protein synthesis_transfer RNA
ENSRNA049449660	tRNA-Trp	tRNA-Trp for anticodon CCA	29	29.2.7	Protein synthesis_transfer RNA
ENSRNA049464941	snoR86	Small nucleolar RNA snoR86	27	27.1	RNA.processing
ENSRNA049464972	snoR86	Small nucleolar RNA snoR86	27	27.1	RNA.processing
ENSRNA049464985	snoR86	Small nucleolar RNA snoR86	27	27.1	RNA.processing
ENSRNA049465003	snoZ279_R105_R108	Small nucleolar RNA Z279/snoR105/snoR108	27	27.1	RNA.processing
ENSRNA049465125	LSU_rRNA_eukarya	Eukaryotic large subunit ribosomal RNA	29	29.2.6	Protein synthesis-ribosomal RNA
ENSRNA049465309	LSU_rRNA_eukarya	Eukaryotic large subunit ribosomal RNA	29	29.2.6	Protein synthesis-ribosomal RNA
ENSRNA049465332	U54	Small nucleolar RNA U54	27	27.1	RNA.processing
ENSRNA049465347	U54	Small nucleolar RNA U54	27	27.1	RNA.processing
ENSRNA049465412	snoR60	Small nucleolar RNA snoR60	27	27.1	RNA.processing
ENSRNA049465951	snoZ169	Small nucleolar RNA Z169	27	27.1	RNA.processing
ENSRNA049466017	snoZ168	Small nucleolar RNA Z168/Z174	27	27.1	RNA.processing
ENSRNA049466121	snoZ168	Small nucleolar RNA Z168/Z174	27	27.1	RNA.processing
ENSRNA049466137	snoZ163	Small nucleolar RNA Z163/Z177 family	27	27.1	RNA.processing
ENSRNA049466338	LSU_rRNA_eukarya	Eukaryotic large subunit ribosomal RNA	29	29.2.6	Protein synthesis-ribosomal RNA
ENSRNA049467117	mir-166	mir-166 microRNA precursor	27	27.1	RNA.processing
ENSRNA049467377	U5	U5 spliceosomal RNA	27	27.1	RNA.processing
ENSRNA049467441	U2	U2 spliceosomal RNA	27	27.1	RNA.processing
ENSRNA049467463	U2	U2 spliceosomal RNA	27	27.1	RNA.processing
ENSRNA049467714	snoR134	small nucleolar RNA snoR134	27	27.1	RNA.processing
ENSRNA049467734	snoZ278	Small nucleolar RNA Z278	27	27.1	RNA.processing
ENSRNA049467742	MIR1428	microRNA MIR1428	27	27.1	RNA.processing
ENSRNA049467928	U1	U1 spliceosomal RNA	27	27.1	RNA.processing
ENSRNA049468027	SNORD36	Small nucleolar RNA SNORD36	27	27.1	RNA.processing
ENSRNA049468126	snoR44_J54	Small nucleolar RNA R44/J54/Z268 family	27	27.1	RNA.processing
ENSRNA049468154	U4	U4 spliceosomal RNA	27	27.1	RNA.processing
ENSRNA049468200	U1	U1 spliceosomal RNA	27	27.1	RNA.processing
ENSRNA049468231	LSU_rRNA_eukarya	Eukaryotic large subunit ribosomal RNA	29	29.2.6	Protein synthesis-ribosomal RNA
ENSRNA049468241	LSU_rRNA_eukarya	Eukaryotic large subunit ribosomal RNA	29	29.2.6	Protein synthesis-ribosomal RNA
ENSRNA049468277	LSU_rRNA_eukarya	Eukaryotic large subunit ribosomal RNA	29	29.2.6	Protein synthesis-ribosomal RNA
ENSRNA049468467	MIR820	microRNA MIR820	27	27.1	RNA.processing
ENSRNA049468702	U6	U6 spliceosomal RNA	27	27.1	RNA.processing
ENSRNA049468709	snoR134	small nucleolar RNA snoR134	27	27.1	RNA.processing
ENSRNA049468722	snoZ223	Small nucleolar RNA Z223	27	27.1	RNA.processing
ENSRNA049468767	MIR820	microRNA MIR820	27	27.1	RNA.processing

ENSRNA049468908	snoU31b	Small nucleolar RNA U31b	27	27.1	RNA.processing
ENSRNA049469003	snoR44_J54	Small nucleolar RNA R44/J54/Z268 family	27	27.1	RNA.processing
ENSRNA049469217	U1	U1 spliceosomal RNA	27	27.1	RNA.processing
ENSRNA049469734	snoZ279_R105_R108	Small nucleolar RNA Z279/snoR105/snoR108	27	27.1	RNA.processing
ENSRNA049469812	U2	U2 spliceosomal RNA	27	27.1	RNA.processing
ENSRNA049470113	snoR117	small nucleolar RNA snoR117	27	27.1	RNA.processing
ENSRNA049470180	U5	U5 spliceosomal RNA	27	27.1	RNA.processing
ENSRNA049470195	MIR171_1	microRNA MIR171_1	27	27.1	RNA.processing
ENSRNA049470386	snoZ278	Small nucleolar RNA Z278	27	27.1	RNA.processing
ENSRNA049470425	U54	Small nucleolar RNA U54	27	27.1	RNA.processing
ENSRNA049470806	U1	U1 spliceosomal RNA	27	27.1	RNA.processing
ENSRNA049470973	U12	U12 minor spliceosomal RNA	27	27.1	RNA.processing
ENSRNA049470992	snoZ221_snoR21b	Small nucleolar RNA Z221/R21b	27	27.1	RNA.processing
ENSRNA049471032	SNORD14	Small nucleolar RNA SNORD14	27	27.1	RNA.processing
ENSRNA049471043	LSU_rRNA_eukarya	Eukaryotic large subunit ribosomal RNA	29	29.2.6	Protein synthesis-ribosomal RNA
ENSRNA049471090	5_8S_rRNA	5.8S ribosomal RNA	29	29.2.6	Protein synthesis-ribosomal RNA
ENSRNA049471102	SSU_rRNA_eukarya	Eukaryotic small subunit ribosomal RNA	29	29.2.6	Protein synthesis-ribosomal RNA
ENSRNA049471325	snoZ102_R77	Small nucleolar RNA Z102/R77	27	27.1	RNA.processing
ENSRNA049471381	MIR159	microRNA MIR159	27	27.1	RNA.processing
ENSRNA049471535	RNase_MRP	RNase MRP	35	35	Unknown
ENSRNA049471546	U4	U4 spliceosomal RNA	27	27.1	RNA.processing
ENSRNA049471602	Plant_SRP	Plant signal recognition particle RNA	27	27.1	RNA.processing
ENSRNA049471778	LSU_rRNA_eukarya	Eukaryotic large subunit ribosomal RNA	27	27.1	RNA.processing
ENSRNA049471985	snoZ118	Small nucleolar RNA Z118/Z121/Z120	27	27.1	RNA.processing
ENSRNA049472003	snoZ118	Small nucleolar RNA Z118/Z121/Z120	27	27.1	RNA.processing
ENSRNA049472018	snoR11	Small nucleolar RNA R11/Z151	27	27.1	RNA.processing
ENSRNA049472073	snoZ118	Small nucleolar RNA Z118/Z121/Z120	27	27.1	RNA.processing
ENSRNA049472089	snoZ152	Small nucleolar RNA Z152/R70/R12/	27	27.1	RNA.processing
ENSRNA049472132	snoZ199	Small nucleolar RNA Z199	27	27.1	RNA.processing
ENSRNA049472195	snoZ118	Small nucleolar RNA Z118/Z121/Z120	27	27.1	RNA.processing
ENSRNA049472208	snoZ118	Small nucleolar RNA Z118/Z121/Z120	27	27.1	RNA.processing
ENSRNA049472231	snoZ118	Small nucleolar RNA Z118/Z121/Z120	27	27.1	RNA.processing
ENSRNA049472320	U2	U2 spliceosomal RNA	27	27.1	RNA.processing
ENSRNA049472337	U2	U2 spliceosomal RNA	27	27.1	RNA.processing
ENSRNA049472543	MIR1428	microRNA MIR1428	27	27.1	RNA.processing
ENSRNA049472690	snoZ105	Small nucleolar RNA Z105	27	27.1	RNA.processing
ENSRNA049472907	SNORD14	Small nucleolar RNA SNORD14	27	27.1	RNA.processing
ENSRNA049472947	SNORD24	Small nucleolar RNA SNORD24	27	27.1	RNA.processing
ENSRNA049473120	snoU31b	Small nucleolar RNA U31b	27	27.1	RNA.processing
ENSRNA049473128	SNORD14	Small nucleolar RNA SNORD14	27	27.1	RNA.processing
ENSRNA049473164	SNORD14	Small nucleolar RNA SNORD14	27	27.1	RNA.processing
ENSRNA049473172	SNORD14	Small nucleolar RNA SNORD14	27	27.1	RNA.processing
ENSRNA049473180	SNORD14	Small nucleolar RNA SNORD14	27	27.1	RNA.processing
ENSRNA049473223	SNORD14	Small nucleolar RNA SNORD14	27	27.1	RNA.processing
ENSRNA049473260	U1	U1 spliceosomal RNA	27	27.1	RNA.processing
ENSRNA049473335	SSU_rRNA_eukarya	Eukaryotic small subunit ribosomal RNA	29	29.2.6	Protein synthesis-ribosomal RNA
ENSRNA049473437	Plant_U3	Plant small nucleolar RNA U3	27	27.1	RNA.processing
ENSRNA049473610	SNORD25	Small nucleolar RNA SNORD25	27	27.1	RNA.processing

ENSRNA049473620	SNORD25	Small nucleolar RNA SNORD25	27	27.1	RNA.processing
ENSRNA049473627	SNORD25	Small nucleolar RNA SNORD25	27	27.1	RNA.processing
ENSRNA049473842	snoZ266	Small nucleolar RNA Z266	27	27.1	RNA.processing
ENSRNA049473876	snoZ103	Small nucleolar RNA Z103	27	27.1	RNA.processing
ENSRNA049473879	snoZ103	Small nucleolar RNA Z103	27	27.1	RNA.processing
ENSRNA049473887	snoZ103	Small nucleolar RNA Z103	27	27.1	RNA.processing
ENSRNA049473899	snoZ223	Small nucleolar RNA Z223	27	27.1	RNA.processing
ENSRNA049473908	snoR134	small nucleolar RNA snoR134	27	27.1	RNA.processing
ENSRNA049473918	U1	U1 spliceosomal RNA	27	27.1	RNA.processing
ENSRNA049474010	MIR171_1	microRNA MIR171_1	27	27.1	RNA.processing
ENSRNA049474050	LSU_rRNA_eukarya	Eukaryotic large subunit ribosomal RNA	29	29.2.6	Protein synthesis-ribosomal RNA
ENSRNA049474061	U5	U5 spliceosomal RNA	27	27.1	RNA.processing
ENSRNA049474711	5S_rRNA	5S ribosomal RNA	29	29.2.6	Protein synthesis-ribosomal RNA
ENSRNA049475361	5S_rRNA	5S ribosomal RNA	29	29.2.6	Protein synthesis-ribosomal RNA
ENSRNA049475575	snoZ43	Small nucleolar RNA Z43	27	27.1	RNA.processing
ENSRNA049475592	U5	U5 spliceosomal RNA	27	27.1	RNA.processing
ENSRNA049475677	SSU_rRNA_eukarya	Eukaryotic small subunit ribosomal RNA	29	29.2.6	Protein synthesis-ribosomal RNA
ENSRNA049475720	U54	Small nucleolar RNA U54	27	27.1	RNA.processing
ENSRNA049475757	LSU_rRNA_eukarya	Eukaryotic large subunit ribosomal RNA	29	29.2.6	Protein synthesis-ribosomal RNA
ENSRNA049475834	U6	U6 spliceosomal RNA	27	27.1	RNA.processing
ENSRNA049475930	snoZ159	Small nucleolar RNA Z159/U59	27	27.1	RNA.processing
ENSRNA049475944	LSU_rRNA_eukarya	Eukaryotic large subunit ribosomal RNA	29	29.2.6	Protein synthesis-ribosomal RNA
ENSRNA049475950	snoR135	small nucleolar RNA snoR135	27	27.1	RNA.processing
ENSRNA049476188	snoZ242	Small nucleolar RNA Z242	27	27.1	RNA.processing
ENSRNA049476209	Plant_U3	Plant small nucleolar RNA U3	27	27.1	RNA.processing
ENSRNA049476293	snoZ242	Small nucleolar RNA Z242	27	27.1	RNA.processing
ENSRNA049476335	snosnR60_Z15	Small nucleolar RNA snR60/Z15/Z230/Z193/J17	27	27.1	RNA.processing
ENSRNA049476340	MIR171_1	microRNA MIR171_1	27	27.1	RNA.processing
ENSRNA049476404	snoZ266	Small nucleolar RNA Z266	27	27.1	RNA.processing
ENSRNA049476411	snoZ266	Small nucleolar RNA Z266	27	27.1	RNA.processing
ENSRNA049476430	SSU_rRNA_eukarya	Eukaryotic small subunit ribosomal RNA	29	29.2.6	Protein synthesis-ribosomal RNA
ENSRNA049476437	SSU_rRNA_eukarya	Eukaryotic small subunit ribosomal RNA	29	29.2.6	Protein synthesis-ribosomal RNA
ENSRNA049476444	SSU_rRNA_eukarya	Eukaryotic small subunit ribosomal RNA	29	29.2.6	Protein synthesis-ribosomal RNA
ENSRNA049476447	SSU_rRNA_eukarya	Eukaryotic small subunit ribosomal RNA	29	29.2.6	Protein synthesis-ribosomal RNA
ENSRNA049476452	SSU_rRNA_eukarya	Eukaryotic small subunit ribosomal RNA	29	29.2.6	Protein synthesis-ribosomal RNA
ENSRNA049476493	5_8S_rRNA	5.8S ribosomal RNA	29	29.2.6	Protein synthesis-ribosomal RNA
ENSRNA049476498	5_8S_rRNA	5.8S ribosomal RNA	29	29.2.6	Protein synthesis-ribosomal RNA
ENSRNA049476502	5_8S_rRNA	5.8S ribosomal RNA	29	29.2.6	Protein synthesis-ribosomal RNA
ENSRNA049476508	5_8S_rRNA	5.8S ribosomal RNA	29	29.2.6	Protein synthesis-ribosomal RNA
ENSRNA049476514	5_8S_rRNA	5.8S ribosomal RNA	29	29.2.6	Protein synthesis-ribosomal RNA
ENSRNA050013642	Intron_gpII	Group II catalytic intron	27	27.1	RNA.processing
ENSRNA050013643	Intron_gpII	Group II catalytic intron	27	27.1	RNA.processing
ENSRNA050013644	5S_rRNA	5S ribosomal RNA	29	29.2.6	Protein synthesis-ribosomal RNA
ENSRNA050013645	5S_rRNA	5S ribosomal RNA	29	29.2.6	Protein synthesis-ribosomal RNA
ENSRNA050013646	Intron_gpII	Group II catalytic intron	27	27.1	RNA.processing
ENSRNA050013647	Intron_gpII	Group II catalytic intron	27	27.1	RNA.processing
ENSRNA050013650	Intron_gpII	Group II catalytic intron	27	27.1	RNA.processing
ENSRNA050013661	Intron_gpII	Group II catalytic intron	27	27.1	RNA.processing

ENSRNA050013662	Intron_gpII	Group II catalytic intron	27	27.1	RNA.processing
ENSRNA050013664	Intron_gpII	Group II catalytic intron	27	27.1	RNA.processing
ENSRNA050013665	Intron_gpII	Group II catalytic intron	27	27.1	RNA.processing
ENSRNA050013666	Intron_gpII	Group II catalytic intron	27	27.1	RNA.processing
ENSRNA050013667	Intron_gpII	Group II catalytic intron	27	27.1	RNA.processing
ENSRNA050013668	Intron_gpII	Group II catalytic intron	27	27.1	RNA.processing
ENSRNA050013672	Intron_gpII	Group II catalytic intron	27	27.1	RNA.processing
EPlORYSAT000373608	rrn26	rRNA	29	29.2.6	Protein synthesis-ribosomal RNA
EPlORYSAT000373610	pseudo-trnV	tRNA pseudogene	29	29.2.7	Protein synthesis_transfer RNA
EPlORYSAT000373611	rrn5	rRNA	29	29.2.6	Protein synthesis-ribosomal RNA
EPlORYSAT000373615	trnR	tRNA	29	29.2.7	Protein synthesis_transfer RNA
EPlORYSAT000373619	trnD	tRNA	29	29.2.7	Protein synthesis_transfer RNA
EPlORYSAT000373647	rrn18	rRNA	29	29.2.6	Protein synthesis-ribosomal RNA
EPlORYSAT000373657	trnS	tRNA	29	29.2.7	Protein synthesis_transfer RNA
EPlORYSAT000373785	tRNA-Ser (UGA)	tRNA	29	29.2.7	Protein synthesis_transfer RNA
EPlORYSAT000373795	tRNA-Thr (GGU)	tRNA	29	29.2.7	Protein synthesis_transfer RNA
EPlORYSAT000373805	tRNA-Val (UAC)	tRNA	29	29.2.7	Protein synthesis_transfer RNA
EPlORYSAT000373810	tRNA-Tyr (GUA)	tRNA	29	29.2.7	Protein synthesis_transfer RNA
EPlORYSAT000373811	rRNA	tRNA	29	29.2.7	Protein synthesis_transfer RNA
EPlORYSAT000373814	tRNA-Ser (GCU)	tRNA	29	29.2.7	Protein synthesis_transfer RNA
EPlORYSAT000373829	rRNA	rRNA	29	29.2.6	Protein synthesis-ribosomal RNA
EPlORYSAT000373834	rRNA	rRNA	29	29.2.6	Protein synthesis-ribosomal RNA
EPlORYSAT000373835	tRNA-Arg (UCU)	tRNA	29	29.2.7	Protein synthesis_transfer RNA
EPlORYSAT000373839	rRNA	rRNA	29	29.2.6	Protein synthesis-ribosomal RNA
EPlORYSAT000373840	tRNA-Asp (GUC)	tRNA	29	29.2.7	Protein synthesis_transfer RNA
EPlORYSAT000373848	tRNA-His (GUG)	tRNA	29	29.2.7	Protein synthesis_transfer RNA
EPlORYSAT000373849	rRNA	rRNA	29	29.2.6	Protein synthesis-ribosomal RNA
EPlORYSAT000373852	rRNA	rRNA	29	29.2.6	Protein synthesis-ribosomal RNA
EPlORYSAT000373862	tRNA-Ile (GAU)	tRNA	29	29.2.7	Protein synthesis_transfer RNA
EPlORYSAT000373867	tRNA-Pro (UGG)	tRNA	29	29.2.7	Protein synthesis_transfer RNA
EPlORYSAT000373872	tRNA-Ile (GAU)	tRNA	29	29.2.7	Protein synthesis_transfer RNA
gene-atp1		ATP synthase subunit alpha	9	9	Mitochondrial electron transport / ATP synthesis
gene-atp6		ATP synthase subunit a	9	9	Mitochondrial electron transport / ATP synthesis
gene-atp9		ATP synthase subunit 9, mitochondrial	9	9	Mitochondrial electron transport / ATP synthesis
gene-cox1		unknown	35	35	Unknown
gene-cox2		Cytochrome c oxidase subunit 2	9	9	Mitochondrial electron transport / ATP synthesis
gene-cox3		Cytochrome c oxidase subunit 3	9	9	Mitochondrial electron transport / ATP synthesis
gene-nad2		unknown	35	35	Unknown
gene-nad4L		NADH dehydrogenase subunit 4L	9	9	Mitochondrial electron transport / ATP synthesis
gene-nad5		unknown	35	35	Unknown
gene-nad6		NADH-ubiquinone oxidoreductase chain 6	9	9	Mitochondrial electron transport / ATP synthesis
gene-nad7		NAD(P)H dehydrogenase subunit H	9	9	Mitochondrial electron transport / ATP synthesis
gene-nad9		NADH dehydrogenase subunit 9	9	9	Mitochondrial electron transport / ATP synthesis
gene-orf25		ATP synthase protein MI25	9	9	Mitochondrial electron transport / ATP synthesis
gene-orfB		H(+)-transporting two-sector ATPase	9	9	Mitochondrial electron transport / ATP synthesis
gene-petE		Cytochrome b6-f complex subunit 5	1	1	Photosyntheisis
gene-psbK		Chloroplast PSII K protein	1	1	Photosyntheisis
gene-rpl36		Large ribosomal subunit protein bL36c	29	29.2.1	Protein.synthesis_ribosomal protein

gene-rps1		Ribosomal protein S1	29	29.2.1	Protein.synthesis_ribosomal protein
Os01g0106400		Similar to Isoflavone reductase homolog IRL (EC 1.3.1.-)	16	16.8.5.1	Secondary metabolism.flavonoids.isoflavones.isoflavone reductase
Os01g0127600		Similar to Bowman-Birk type proteinase inhibitor D-II precursor (IV)	35	35.2	Unknown
Os01g0148000		E3 ubiquitin-protein ligase	29	29.5.11	Protein degradation ubiquitin
Os01g0179600	OsUGT75K1	UDP-glucuronosyl/UDP-glucosyltransferase family protein	26	26.2	Misc.UDP glucosyl and glucoronyl transferases
Os01g0209500		Zinc finger%2C RING-type domain containing protein	27	27.3.67	RNA.regulation of transcription.putative transcription regulator
Os01g0209566		Hypothetical protein	35	35	Unknown
Os01g0212000		Hypothetical protein	35	35	Unknown
Os01g0221400		Conserved hypothetical protein	35	35.2	Unknown
Os01g0237200		Conserved hypothetical protein	35	35	Unknown
Os01g0252300		Conserved hypothetical protein	35	35.2	Unknown
Os01g0262500		TRAM/LAG1/CLN8 homology domain domain containing protein	35	35.2	Unknown
Os01g0281301		Protein of unknown function DUF1645 domain containing protein	35	35.2	Unknown
Os01g0287400		Similar to Hydrophobic protein LTI6A (Low temperature-induced protein 6A)	35	35	Unknown
Os01g0288700		Hypothetical gene	35	35	Unknown
Os01g0303000		Similar to CP12 (Fragment)	1	1.3	Photosyntheisis
Os01g0323775		Hypothetical protein	35	35	Unknown
Os01g0331500		Non-protein coding transcript	35	35	Unknown
Os01g0361700		Conserved hypothetical protein	11	11.7	Lipid metabolism.unassigned
Os01g0371200	OsGSTF1	Similar to Glutathione-S-transferase 19E50	26	26.9	misc.glutathione S transferases
Os01g0374000	OsGSTF12	Similar to Glutathione S-transferase I (EC 2.5.1.18) (GST-I) (GST-29) (GST class- phi)	26	26.9	misc.glutathione S transferases
Os01g0374100		Non-protein coding transcript	35	35	Unknown
Os01g0384450		Similar to H0124E07.4 protein	35	35.2	Unknown
Os01g0387566		Conserved hypothetical protein	35	35	Unknown
Os01g0392600		Conserved hypothetical protein	35	35.2	Unknown
Os01g0444100		Conserved hypothetical protein	35	35.2	Unknown
Os01g0524251		Conserved hypothetical protein	35	35	Unknown
Os01g0541800	OsSWEET2a	MtN3 and saliva related transmembrane protein family protein	33	33.99	Development.unspecified
Os01g0550250		Non-protein coding transcript	35	35	Unknown
Os01g0559550		Non-protein coding transcript	33	33.99	Development.unspecified
Os01g0576050		Hypothetical conserved gene	35	35	Unknown
Os01g0579900		Esterase/lipase/thioesterase domain containing protein	35	35.2	Unknown
Os01g0586900		Non-protein coding transcript	35	35	Unknown
Os01g0593700	OsSultr3	Sulphate transporter domain containing protein	34	34.6	Transport sulphate
Os01g0594500		Conserved hypothetical protein	35	35.2	Unknown
Os01g0606900	OsDjC10	Heat shock protein DnaJ%2C N-terminal domain containing protein	20	20.2.1	Stress.abiotic.heat
Os01g0626350		Non-protein coding transcript	35	35	Unknown
Os01g0634600	OsPME4	Pectin lyase fold/virulence factor domain containing protein	10	10.8.1	Cell wall.pectin*esterases.PME
Os01g0639900	BETACA1	beta-carbonic anhydrase (EC:4.2.1.1)%2C Carbon assimilation%2C CO₂-mediated stomatal pore respons	8	8.3	TCA / org. transformation
Os01g0652650		Non-protein coding transcript	35	35	Unknown

Os01g0656600		Conserved hypothetical protein	35	35.2	Unknown
Os01g0667550		Hypothetical gene	35	35	Unknown
Os01g0678900		Non-protein coding transcript	35	35	Unknown
Os01g0682001		Similar to NADH dependent Glutamate Synthase	12	12.2.1	N-metabolism.ammonia metabolism.glutamate synthase
Os01g0687500		Methionine tRNA Formyltransferase-like domain containing protein	13	13.2.3.4	amino acid metabolism.degradation.aspartate family.methionine
Os01g0692000	OsGSTU40	Similar to Glutathione S-transferase GSTU6	26	26.9	misc.glutathione S transferases
Os01g0702450		Similar to Chaperone protein dnaJ 10	9	9	Mitochondrial electron transport / ATP synthesis
Os01g0702900		Similar to Sucrose-phosphate synthase (EC 2.4.1.14)	27	27.1	RNA.processing
Os01g0725000		Similar to H0402C08.3 protein	35	35.2	Unknown
Os01g0725900		Similar to arabinogalactan protein	35	35.2	Unknown
Os01g0733200	OsHsfC1b	Similar to Heat shock transcription factor 29 (Fragment)	27	27.3.23	RNA.regulation of transcription.HSF,Heat-shock transcription factor family
Os01g0734150		Hypothetical gene	35	35	Unknown
Os01g0734200	rbohA	Similar to respiratory burst oxidase protein B	9	9	Mitochondrial electron transport / ATP synthesis
Os01g0737200		Non-protein coding transcript	35	35	Unknown
Os01g0741250		Hypothetical protein	35	35	Unknown
Os01g0741900	IAA6	Auxin-responsive protein%2C Drought tolerance%2C Control of tiller outgrowt	17	17.2.3	Hormone metabolism.auxin.induced-regulated-responsive-activated
Os01g0745850		Hypothetical protein	35	35	Unknown
Os01g0748150	OsENODL1	Cupredoxin domain containing protein	26	26.19	misc.plastocyanin-like
Os01g0750300	OsCesA4	Similar to Cellulose synthase (Fragment)	10	10.2.1	Cell wall.cellulose synthesis.cellulose synthase
Os01g0760701		Hypothetical gene	35	35	Unknown
Os01g0761400		TGF-beta receptor%2C type I/II extracellular region family protein	34	34.13	transport.peptides and oligopeptides
Os01g0778500		Similar to predicted protein	35	35.2	Unknown
Os01g0787600	OsPOP4	Similar to Salicylic acid-binding protein 2	26	26.8	Misc.nitrilases, *nitrile lyases, berberine bridge enzymes, reticuline oxidases, troponine reductases
Os01g0794400		Thioredoxin domain 2 containing protein	35	35.2	Unknown
Os01g0798200		Conserved hypothetical protein	35	35	Unknown
Os01g0805900	OsTUB4	Tubulin beta-2 chain (Beta-2 tubulin)	31	31.1	Cell organisation
Os01g0812100		Similar to NHL25	33	33.2	Development.late embryogenesis abundant
Os01g0819300	OsNTMC2T1.3	Similar to calcium lipid binding protein-like	30	30.3	Signalling.calcium
Os01g0825800	OsATL7	Amino acid transporter%2C transmembrane domain containing protein	34	34.3	Transport.amino acids
Os01g0829400	OsGRL2	Thioredoxin fold domain containing protein	21	21.4	redox.glutaredoxins
Os01g0829600		Protein of unknown function DUF688 domain containing protein	35	35.2	Unknown
Os01g0840100	OsMed37_1	Heat shock protein Hsp70 family protein	20	20.2.1	Stress
Os01g0846700		Hypothetical protein	35	35	Unknown
Os01g0846800		Regulator of chromosome condensation%2C RCC1 domain containing protein	31	31.2	Cell division
Os01g0860601		Similar to Ferredoxin%2C root R-B1	1	1.1.5.2	Photosyntheisis
Os01g0872100		TGF-beta receptor%2C type I/II extracellular region family protein	34	34.13	Transport.peptides and oligopeptides
Os01g0880300	OsPME7	Similar to Pectin methylesterase-like protein	10	10.8.1	Cell wall.pectin*esterases.PME
Os01g0899387		Conserved hypothetical protein	3	3.6	minor CHO metabolism
Os01g0909150		Conserved hypothetical protein	35	35	Unknown
Os01g0915300		Non-protein coding transcript	35	35	Unknown

Os01g0915401		Proteinase inhibitor I25%2C cystatin domain containing protein	35	35.2	Unknown
Os01g0926300		Similar to Transaldolase (EC 2.2.1.2)	7	7.2.2	OPP
Os01g0937100		Similar to Xylanase inhibitor precursor (Xylanase inhibitor TAXI-I)	29	29.5.4	Protein.degradation.aspartate protease
Os01g0939901		Non-protein coding transcript	35	35	Unknown
Os01g0958200		Curculin-like (mannose-binding) lectin domain containing protein	29	29.4	Protein.postranslational modification
Os01g0971800	OsPCL1	Transcription factor with a GARP DNA-binding domain%2C Photoperiodic control of flowering time%2C Clock associated-componen	27	27.3.20	RNA.regulation of transcription.G2-like transcription factor family, GARP
Os01g0974400	XBOS31	Zinc finger%2C RING/FYVE/PHD-type domain containing protein	31	31.1	Cell organisation
Os01g0974600		Similar to Histone H2A	35	35	Unknown
Os02g0102900	OsCPn60beta2	Similar to RuBisCO subunit binding-protein beta subunit%2C chloroplast (60 kDa chaperonin beta subunit) (CPN-60 beta) (Fragment)	29	29.6	Protein.folding
Os02g0106966		Similar to emb1507 (embryo defective 1507)%3B ATP binding / ATP-dependent helicase/ helicase/ nucleic acid binding / nucleoside-triphosphatase/ nucleotide binding	9	9	Mitochondrial electron transport / ATP synthesis
Os02g0115950		Glutamate dehydrogenase%2C NAD-specific domain containing protein	9	9	Mitochondrial electron transport / ATP synthesis
Os02g0129000		Hypothetical conserved gene	4	4.1.6	Protein.postranslational modification.kinase.receptor like cytoplasmatic kinase X
Os02g0129800		Conserved hypothetical protein	35	35.2	Unknown
Os02g0134200	OsSGL	DUF1645 family protein%2C Regulation of stress-tolerance and grain lengt	20	20	Stress
Os02g0152800		RNA polymerase Rpb1%2C domain 1 containing protein	27	27.2	RNA.transcription
Os02g0175950		Non-protein coding transcript	35	35	Unknown
Os02g0187300		Similar to predicted protein	35	35.2	Unknown
Os02g0189400		Defence response%2C Rin4 domain containing protein	30	30.1	Signalling.in sugar and nutrient physiology
Os02g0191300	OsATL6	Similar to Amino acid transporter-like protein	34	34.3	Transport.amino acids
Os02g0191700		EF-HAND 2 domain containing protein	30	30.3	Signalling.calcium
Os02g0192250		Non-protein coding transcript	35	35	Unknown
Os02g0193967		Non-protein coding transcript	35	35	Unknown
Os02g0226350		Non-protein coding transcript	34	34.9	Transport.metabolite transporters at the mitochondrial membrane
Os02g0227100		Conserved hypothetical protein	35	35.2	Unknown
Os02g0244450		Non-protein coding transcript	35	35	Unknown
Os02g0257400		Non-protein coding transcript	26	26.23	Misc.rhodanese
Os02g0301450		Hypothetical gene	35	35	Unknown
Os02g0322251		Hypothetical protein	35	35	Unknown
Os02g0322400		Similar to NPKL2 (Fragment)	29	29.4	Protein.postranslational modification
Os02g0452700		Hypothetical protein	35	35.2	Unknown
Os02g0514500		Similar to Glycerophosphoryl diester phosphodisterase (Fragment)	11	11.9.3.3	Lipid metabolism.lipid degradation.lysophospholipases.glycerophosphodiester phosphodiesterase
Os02g0519700		Conserved hypothetical protein	35	35.2	Unknown
Os02g0527200		Similar to OSIGBa0142C11.2 protein	35	35.2	Unknown
Os02g0533200		SOUL haem-binding protein domain containing protein	21	21.3	redox.heme
Os02g0541300		Hypothetical conserved gene	35	35	Unknown
Os02g0549850		Non-protein coding transcript	35	35	Unknown

Os02g0553802		Hypothetical conserved gene	35	35	Unknown
Os02g0563000	OsKMD3	Hypothetical conserved gene	29	29.5.11.4.3.2	Protein.degradation.ubiquitin.E3.SCF.FBOX
Os02g0580500		Similar to Prohibitin	9	9.1.1	Mitochondrial electron transport / ATP synthesis.NADH-DH.complex I
Os02g0586900	OsEnS-40	Hypothetical conserved gene	35	35.2	Unknown
Os02g0594232		Hypothetical conserved gene	35	35	Unknown
Os02g0594700		Similar to Non-phototropic hypocotyl 3	30	30.11	Signalling.light
Os02g0604000	OsGELP38	Similar to alpha-L-fucosidase 2	26	26.28	Misc.GDSL-motif lipase
Os02g0629000		Protein of unknown function DUF584 family protein	35	35.2	Unknown
Os02g0630250		Similar to predicted protein	35	35.2	Unknown
Os02g0635400		Non-protein coding transcript	35	35.2	Unknown
Os02g0653300		Conserved hypothetical protein	35	35	Unknown
Os02g0666250		Hypothetical gene	35	35	Unknown
Os02g0670500	OsRALF-8	Rapid ALkalinization Factor family protein	30	30.8	Signalling.misc
Os02g0685200	OsMyb1R	MYB transcription factor%2C Transcriptional activator in mediating stress and rhythm responsivegene expressio	27	27.3.26	RNA.regulation of transcription.MYB-related transcription factor family
Os02g0688800		Non-protein coding transcript	29	29.5.11.4.2	Protein.degradation.ubiquitin.E3.RING
Os02g0693500		Similar to OSIGBa0142I02-OSIGBa0101B20.17 protein	35	35.2	Unknown
Os02g0708500		Conserved hypothetical protein	27	27.3.11	RNA.regulation of transcription.C2H2 zinc finger family
Os02g0722300		Nonaspanin (TM9SF) family protein	28	28.99	DNA.unspecified
Os02g0728850		Non-protein coding transcript	35	35	Unknown
Os02g0730075		Hypothetical protein	35	35	Unknown
Os02g0732800	OsGELP43	Similar to Anther-specific proline-rich protein APG	26	26.28	Misc.GDSL-motif lipase
Os02g0738600	OsGH9B8	Similar to CEL5%3DCELLULASE 5 (Fragment)	26	26.3.4	Misc.gluco-, galacto- and mannosidases.endoglucanase
Os02g0761500		Conserved hypothetical protein	35	35	Unknown
Os02g0772000		Similar to global transcription factor group	27	27.3.52	RNA.regulation of transcription.Global transcription factor group
Os02g0783650		Hypothetical gene	35	35	Unknown
Os02g0783700		Similar to Lysine ketoglutarate reductase/saccharopine dehydrogenase	13	13.2.3.5	Amino acid metabolism.degradation.aspartate family.lysine
Os02g0801255		Similar to cDNA clone:J013118P06%2C full insert sequence	35	35.2	Unknown
Os02g0822050		Hypothetical gene	35	35	Unknown
Os03g0111200	OsREM1.3	Similar to Remorin	27	27.3.99	RNA.regulation of transcription.unclassified
Os03g0111400		Heavy metal transport/detoxification protein domain containing protein	35	35.2	Unknown
Os03g0112900	OsF5HL2	Similar to Aldehyde 5-hydroxylase	26	26.1	Misc.misc2
Os03g0117100	PEX11-1	Peroxisomal protein%2C Response to salt and low nitrogen stresse	29	29.3.5	Protein.targeting.peroxisomes
Os03g0126700		Similar to Barley stem rust resistance protein	28	28.2	DNA.repair
Os03g0128600		Conserved hypothetical protein	35	35.2	Unknown
Os03g0133500	OsACS	Similar to Adenosine monophosphate binding protein 1 AMPBP1	11	11.1.8	Lipid metabolism.FA synthesis and FA elongation.acyl coa ligase
Os03g0133850		Hypothetical gene	35	35	Unknown
Os03g0137250		Non-protein coding transcript	35	35	Unknown
Os03g0145751		Hypothetical genes	35	35	Unknown
Os03g0146800	OsIPS1	Non-protein coding transcript	35	35.2	Unknown
Os03g0180100		Protein of unknown function DUF1677%2C plant domain containing protein	35	35.2	Unknown
Os03g0192750		Hypothetical gene	35	35	Unknown

Os03g0204500		Conserved hypothetical protein	35	35.2	Unknown
Os03g0226200	HB2	Non-symbiotic hemoglobin 2 (rHb2	21	21.3	redox.heme
Os03g0232400		Phytosulfokine family protein	35	35.2	Unknown
Os03g0237500		Similar to predicted protein	35	35.2	Unknown
Os03g0245800	OsHSP26	Similar to Heat shock protein 26	20	20.2.1	Stress
Os03g0254250		Non-protein coding transcript	35	35.2	Unknown
Os03g0262200		Similar to predicted protein	29	29.4	Protein.postranslational modification
Os03g0265900		Conserved hypothetical protein	35	35.2	Unknown
Os03g0266100		Zinc finger%2C LIM-type domain containing protein	33	33.99	Development.unspecified
Os03g0267100		Hypothetical protein	35	35	Unknown
Os03g0267200	OsHsp17.7	Low molecular mass heat shock protein Oshsp17.7	20	20.2.1	Stress
Os03g0276500	OsHsp71.1	Similar to Heat shock protein 70	20	20.2.1	Stress
Os03g0290900	OsTBL18	Protein of unknown function DUF231%2C plant domain containing protein	35	35.2	Unknown
Os03g0293000	OsERdj3A	Similar to DnaJ domain containing protein%2C expressed	20	20.2.1	Stress
Os03g0299700		Conserved hypothetical protein	35	35.2	Unknown
Os03g0306900		Haem oxygenase-like%2C multi-helical domain containing protein	35	35.2	Unknown
Os03g0351700		Conserved hypothetical protein	35	35.2	Unknown
Os03g0369100	OsLTPL36	Bifunctional inhibitor/plant lipid transfer protein/seed storage domain containing protein	11	11.6	Lipid metabolism.lipid transfer proteins etc
Os03g0379650		Hypothetical gene	35	35	Unknown
Os03g0381300		CS domain domain containing protein	35	35.2	Unknown
Os03g0388432		Non-protein coding transcript	35	35	Unknown
Os03g0407050		Hypothetical protein	35	35.2	Unknown
Os03g0409400		Similar to mps one binder kinase activator-like 1A	27	27.3.99	RNA.regulation of transcription.unclassified
Os03g0432277		Hypothetical protein	35	35	Unknown
Os03g0441050		Hypothetical protein	35	35	Unknown
Os03g0567100		Conserved hypothetical protein	35	35.2	Unknown
Os03g0586500		Similar to chloroplast post-illumination chlorophyll fluorescence increase protein	1	1.1.40	Photosyntheisis
Os03g0618366		Non-protein coding transcript	35	35	Unknown
Os03g0629800		Conserved hypothetical protein	35	35.2	Unknown
Os03g0643611		Hypothetical protein	35	35	Unknown
Os03g0648450		Hypothetical gene	35	35	Unknown
Os03g0663800	OsEnS-51	Globulin 1 (Fragment)	33	33.1	Development.storage proteins
Os03g0679700	OsTHIC	Similar to Thiamine biosynthesis protein thiC	18	18.2	Co-factor and vitamine metabolism.thiamine
Os03g0682200	OsAGO12	Similar to Protein argonaute 12	33	33.99	Development.unspecified
Os03g0721900		Similar to Histone H2A	28	28.1.3	DNA.synthesis/chromatin structure.histone
Os03g0722500		Glycoside hydrolase%2C family 17 protein	26	26.4.1	Misc.beta 1,3 glucan hydrolases.glucan endo-1,3-beta-glucosidase
Os03g0726300	OsOSCA2.3	Protein of unknown function DUF221 domain containing protein	20	20.2.3	Stress.abiotic.drought/salt
Os03g0738350		Non-protein coding transcript	35	35	Unknown
Os03g0745000	HSfA2a	Similar to Heat stress transcription factor A-2a	27	27.2	RNA.transcription
Os03g0764250		Hypothetical gene	35	35	Unknown
Os03g0764300	OsMAPKKK18	Serine/threonine protein kinase domain containing protein	29	29.4	Protein.postranslational modification
Os03g0765400		Similar to nucleoporin p58/p45	29	29.3.1	Protein.targeting.nucleus
Os03g0768450		Hypothetical conserved gene	35	35	Unknown

Os03g0776900	OsDjC35	Similar to mitochondrial import inner membrane translocase subunit TIM14	20	20.2.1	Stress
Os03g0794000	OsLTPG9	Plant lipid transfer protein/Par allergen family protein	11	11.6	Lipid metabolism.lipid transfer proteins etc
Os03g0797400	OsIGL	Similar to Indole-3-glycerol phosphate lyase	13	13.1.6.5.5	Amino acid metabolism.synthesis.aromatic aa.tryptophan.tryptophan synthase
Os03g0808500	OsLTP1.3	Plant lipid transfer protein/Par allergen family protein	11	11.6	Lipid metabolism.lipid transfer proteins etc
Os03g0810700		Similar to Mitochondrial Rho GTPase	30	30.5	Signalling.G-proteins
Os03g0821275		Non-protein coding transcript	35	35	Unknown
Os03g0826800		Conserved hypothetical protein	35	35.2	Unknown
Os03g0828500		Similar to (1-4)-beta-mannan endohydrolase precursor (EC 3.2.1.78)	10	10.6.2	Cell wall.degradation.mannan-xylose-arabinose-fucose
Os03g0832200	CRT	Similar to Calcium-binding protein precursor (Calreticulin)	30	30.3	Signalling.calcium
Os03g0844301		Hypothetical gene	35	35	Unknown
Os04g0103601		Conserved hypothetical protein	35	35	Unknown
Os04g0105050		Hypothetical gene	35	35	Unknown
Os04g0107900	Hsp90	Similar to Heat shock protein 82	20	20.2.1	Stress
Os04g0118450		Hypothetical protein	35	35	Unknown
Os04g0119300		Similar to H0716A07.6 protein	35	35.2	Unknown
Os04g0162500		Zinc finger%2C C2H2-type domain containing protein	27	27.3	RNA.regulation of transcription
Os04g0173800		Lectin precursor (Agglutinin)	20	20.1	Stress.biotic
Os04g0210700		Similar to OSIGBa0114M03.3 protein	1	1	Photosyntheisis
Os04g0223500		Dimethylaniline monooxygenase%2C N-oxide-forming domain containing protein	26	26.7	Misc.oxidases - copper, flavone etc.
Os04g0252850		Hypothetical gene	35	35	Unknown
Os04g0372775		Non-protein coding transcript	35	35	Unknown
Os04g0409200	OsBEIIa	2.1.2.3			
Os04g0412000		Hypothetical protein	35	35.2	Unknown
Os04g0429087		Non-protein coding transcript	35	35	Unknown
Os04g0445100		Similar to 22.7 kDa class IV heat shock protein precursor	20	20.2.1	Stress
Os04g0445700		Similar to OSIGBa0140O07.8 protein	11	11.1.3	Lipid metabolism.FA synthesis and FA elongation.ketoacyl ACP synthase
Os04g0450900		Protein kinase PKN/PRK1%2C effector domain containing protein	29	29.4	Protein.postranslational modification
Os04g0479550		Hypothetical protein	35	35	Unknown
Os04g0481050		Hypothetical gene	35	35	Unknown
Os04g0481200		Similar to H0510A06.2 protein	26	26.3.5	Misc.gluco-, galacto- and mannosidases.glycosyl hydrolase family 5
Os04g0483450		Hypothetical conserved gene	35	35	Unknown
Os04g0485700		Similar to Exonuclease	35	35.2	Unknown
Os04g0502900		EF-Hand type domain containing protein	30	30.3	Signalling.calcium
Os04g0511200	EFA27	EFA27 for EF hand%2C abscisic acid%2C 27kD	33	33.99	Development.unspecified
Os04g0522100	DEFL12	Similar to OSIGBa0131L05.12 protein	35	35.2	Unknown
Os04g0526800		GRAM domain containing protein	17	17.1.3	Hormone metabolism.abscisic acid.induced-regulated-responsive-activated
Os04g0527000		GRAM domain containing protein	17	17.1.3	Hormone metabolism.abscisic acid.induced-regulated-responsive-activated
Os04g0528000	OsEnS-71	Similar to OSIGBa0115K01-H0319F09.15 protein	35	35.2	Unknown
Os04g0533602		Hypothetical genes	35	35	Unknown
Os04g0538166		Similar to Elongation factor G%2C chloroplastic	29	29.2.4	Protein.synthesis.elongation
Os04g0552300	CycU2	Cyclin-related 2 domain containing protein	31	31.3	Cell cycle

Os04g0560600	CDPK12	Calcium-dependent protein kinase%2C Salt-stress tolerance%2C Blast disease resistanc	30	30.3	Signalling.calcium
Os04g0563350		Conserved hypothetical protein	35	35.2	Unknown
Os04g0573700		Non-protein coding transcript	35	35	Unknown
Os04g0583900	LHY	Similar to LHY protein	27	27.3.26	RNA.regulation of transcription.MYB-related transcription factor family
Os04g0587200		Similar to invertase inhibitor	26	26.18	Misc.invertase/pectin methylesterase inhibitor family protein
Os04g0602250		Non-protein coding transcript	35	35	Unknown
Os04g0614300		Hypothetical conserved gene	35	35	Unknown
Os04g0615200		Hypothetical protein	35	35.2	Unknown
Os04g0616250		Hypothetical gene	35	35	Unknown
Os04g0617050	OsSAUR21	Similar to H0313F03.1 protein	17	17.2.3	Hormone metabolism.auxin.induced-regulated-responsive-activated
Os04g0617500		Similar to H0313F03.4 protein	35	35	Unknown
Os04g0625850		Hypothetical protein	35	35	Unknown
Os04g0641101		Non-protein coding transcript	35	35	Unknown
Os04g0659800	OsAAP7B	Similar to OSIGBa0132E09-OSIGBa0108L24.20 protein	34	34.3	Transport.amino acids
Os04g0661250		Hypothetical gene	35	35	Unknown
Os04g0669900	OsMTK2	Similar to H0818H01.11 protein	13	13.99	Amino acid metabolism.misc
Os04g0673300	RR6	A-type response regulator%2C Cytokinin signalin	27	27.3.5	RNA.regulation of transcription.ARR
Os04g0674025		Similar to H0403D02.10 protein	35	35	Unknown
Os04g0675400	OsDjC45	Similar to Chaperone protein dnaJ	20	20.2.1	Stress
Os04g0681600		Protein of unknown function DUF580 family protein	34	34.99	Transport.misc
Os04g0682100		C2 calcium/lipid-binding region%2C CaLB domain containing protein	35	35.2	Unknown
Os04g0682400		Similar to H0124B04.13 protein	27	27.1	RNA.processing
Os04g0684800	OsUEV1D	Similar to CROC-1-like protein (Fragment)	29	29.5.11.3	Protein.degradation.ubiquitin.E2
Os05g0100100		Similar to cDNA clone:006-204-G09%2C full insert sequence	35	35.2	Unknown
Os05g0102700		Hypothetical protein	35	35	Unknown
Os05g0103400		Protein of unknown function DUF3339 domain containing protein	35	35.2	Unknown
Os05g0103800		Conserved hypothetical protein	35	35.2	Unknown
Os05g0104700	PGIP4	Leucine-rich repeat (LRR) protein%2C Inhibitor of fungal polygalacturonase%2C Defence respons	10	10.6.3	Cell wall.degradation.pectate lyases and polygalacturonases
Os05g0121725		Non-protein coding transcript	35	35	Unknown
Os05g0132601		Non-protein coding transcript	35	35	Unknown
Os05g0156900	Chalk5	Vacuolar H+-translocating pyrophosphatase%2C Regulation of grain chalkines	34	34.3	Transport.amino acids
Os05g0167300		Hypothetical conserved gene	35	35.2	Unknown
Os05g0179950		Similar to cDNA%2C clone: J100088D24%2C full insert sequence	26	26.2	Misc.UDP glucosyl and glucoronyl transferases
Os05g0217000	OsAGP23	Protein of unknown function DUF1070 family protein	35	35.2	Unknown
Os05g0228900		Hypothetical protein	35	35.2	Unknown
Os05g0270500	OsRPP14.1	Similar to predicted protein	18	18	Co-factor and vitamine metabolism
Os05g0328300	OsOFP33	Similar to OFP17 (ARABIDOPSIS THALIANA OVATE FAMILY PROTEIN 17)	35	35.2	Unknown
Os05g0328333	pro13b.5	Similar to Prolamin	33	33.1	Development.storage proteins
Os05g0328466	pro13b.6	Similar to Prolamin	33	33.1	Development.storage proteins
Os05g0328901	pro13b.9	Similar to Prolamin	33	33.1	Development.storage proteins
Os05g0329001	pro13b.10	Similar to Prolamin	33	33.1	Development.storage proteins

Os05g0329100	pro13b.11	Prolamin	33	33.1	Development.storage proteins
Os05g0329200	pro13b.16	Similar to Prolamin	33	33.1	Development.storage proteins
Os05g0329300	pro13b.12	Prolamin	33	33.1	Development.storage proteins
Os05g0329350	pro13b.13	Prolamin	33	33.1	Development.storage proteins
Os05g0329400	pro13b.14	Similar to Prolamin	33	33.1	Development.storage proteins
Os05g0329700	pro13b.15	Similar to Prolamin	33	33.1	Development.storage proteins
Os05g0330150	pro13b.17	Similar to Prolamin	33	33.1	Development.storage proteins
Os05g0330600	pro13b.18	Similar to Prolamin	33	33.1	Development.storage proteins
Os05g0331366	pro13b.19	Similar to Prolamin	33	33.1	Development.storage proteins
Os05g0331532	pro13b.20	Similar to Prolamin	33	33.1	Development.storage proteins
Os05g0331800	pro13b.21	Similar to Prolamin	33	33.1	Development.storage proteins
Os05g0331900		Conserved hypothetical protein	35	35.2	Unknown
Os05g0332000	pro13b.22	Similar to Prolamin precursor	33	33.1	Development.storage proteins
Os05g0370650		Hypothetical protein	35	35	Unknown
Os05g0392050		Hypothetical conserved gene	35	35	Unknown
Os05g0395400		Hypothetical protein	35	35.2	Unknown
Os05g0405400		Hypothetical conserved gene	35	35.2	Unknown
Os05g0407100		Four F5 protein family protein	35	35.2	Unknown
Os05g0433100		Similar to Serine/threonine-protein kinase SAPK4	29	29.4	Protein.postranslational modification
Os05g0437401		Conserved hypothetical protein	35	35.2	Unknown
Os05g0438500		23.4.99			
Os05g0440150		Hypothetical gene	35	35	Unknown
Os05g0456925		Similar to regulator of chromosome condensation (RCC1) family protein	31	31.2	Cell division
Os05g0460000	OsctHSP70-1	Similar to 70 kDa heat shock cognate protein 1	20	20.2.1	Stress
Os05g0474600		Similar to Aldose reductase-related protein (EC 1.1.1.21)	33	33.99	Development.unspecified
Os05g0517400		Conserved hypothetical protein	35	35.2	Unknown
Os05g0519000	OsVQ21	VQ domain containing protein	35	35.2	Unknown
Os05g0519700	HSP101	Heat shock protein%2C Long-term acquired thermotoleranc	20	20.2.1	Stress
Os05g0535800		Similar to F14O23.10 protein	29	29.2.1.1.3.1.1	Protein.synthesis.ribosomal protein.prokaryotic.unknown organellar.30S subunit.S1
Os05g0561600		Protein of unknown function DUF1421 domain containing protein	10	10.5.4	Cell wall.cell wall proteins.HRGP
Os05g0570000		Non-protein coding transcript	35	35	Unknown
Os05g0595100	OsUGE1	Uridine-diphospho-(UDP)-glucose 4-epimerase%2C Cell wall carbohydrate partitioning during nitrogen (N) limitatio	10	10.1.2	Cell wall.precursor synthesis.UGE
Os06g0102725		Hypothetical protein	35	35	Unknown
Os06g0107700	OsLFNR2	1.1.5.3			
Os06g0110200	OsLEA2	Late embryogenesis abundant (LEA) group 1 family protein	33	33.2	Development.late embryogenesis abundant
Os06g0131001		Conserved hypothetical protein	35	35	Unknown
Os06g0133400		Conserved hypothetical protein	35	35.2	Unknown
Os06g0141600	OsENOD93	Early nodulin 93 ENOD93 protein family protein	35	35.2	Unknown
Os06g0142200	OSENOD93A	Early nodulin	35	35.2	Unknown
Os06g0142350	UDP-GT	Similar to Early nodulin	35	35.2	Unknown
Os06g0142400	OsENOD93	Early nodulin	35	35.2	Unknown
Os06g0147100		Similar to cDNA clone:J023088C01%2C full insert sequence	35	35.2	Unknown
Os06g0147500		Conserved hypothetical protein	35	35	Unknown
Os06g0157125		Hypothetical protein	35	35	Unknown
Os06g0160200	OsGELP80	Similar to Lanatoside 15'-O-acetylesterase precursor	26	26.28	Misc.GDSL-motif lipase

Os06g0167125		Non-protein coding transcript	35	35	Unknown
Os06g0169800		Hypothetical protein	35	35	Unknown
Os06g0181100		Octicosapeptide/Phox/Bem1p domain containing protein	35	35	Unknown
Os06g0184766		Hypothetical conserved gene	34	34.16	Transport.ABC transporters and multidrug resistance systems
Os06g0192676		Non-protein coding transcript	35	35	Unknown
Os06g0197550		Similar to Chaperonin	20	20.2	Stress.abiotic
Os06g0197575		Similar to Chaperonin	29	29.6	Protein.folding
Os06g0219900		Similar to Phi-1 protein	30	30.1.1	Signalling.in sugar and nutrient physiology
Os06g0220100		Hypothetical protein	35	35	Unknown
Os06g0220900		Conserved hypothetical protein	35	35.2	Unknown
Os06g0223900		Non-protein coding transcript	35	35	Unknown
Os06g0255200		Conserved hypothetical protein	28	28.1	DNA.synthesis/chromatin structure
Os06g0259901		Non-protein coding transcript	35	35	Unknown
Os06g0261300		Conserved hypothetical protein	35	35.2	Unknown
Os06g0264700		Acylphosphatase domain containing protein	35	35.2	Unknown
Os06g0313000		Conserved hypothetical protein	35	35	Unknown
Os06g0330250		Non-protein coding transcript	35	35	Unknown
Os06g0340001		Hypothetical conserved gene	35	35.2	Unknown
Os06g0505202		Hypothetical conserved gene	35	35.2	Unknown
Os06g0506850		Non-protein coding transcript	35	35	Unknown
Os06g0513050	OsTHI3	Conserved hypothetical protein	20	20.1	Stress.biotic
Os06g0513943	OsTHI6	Conserved hypothetical protein	20	20.1	Stress.biotic
Os06g0514100	Osthi7	Conserved hypothetical protein	20	20.1	Stress.biotic
Os06g0523300		Conserved hypothetical protein	35	35.2	Unknown
Os06g0558766		Similar to predicted protein	27	27.1	RNA.processing
Os06g0559400		Conserved hypothetical protein	35	35.2	Unknown
Os06g0566201		Hypothetical gene	35	35	Unknown
Os06g0573600	OsBgal2	Similar to Beta-galactosidase precursor (EC 3.2.1.23) (Lactase)	26	26.3.2	Misc.gluco-, galacto- and mannosidases.beta-galactosidase
Os06g0582400		Conserved hypothetical protein	35	35.2	Unknown
Os06g0611100		Similar to IQ calmodulin-binding motif family protein	35	35	Unknown
Os06g0611300		Conserved hypothetical protein	35	35.2	Unknown
Os06g0614400		Conserved hypothetical protein	35	35.2	Unknown
Os06g0622550		Conserved hypothetical protein	35	35.2	Unknown
Os06g0639550		Non-protein coding transcript	35	35	Unknown
Os06g0643750		Hypothetical gene	35	35	Unknown
Os06g0664300		Similar to Vacuolar sorting receptor 6 precursor (AtVSR6) (Epidermal growth factor receptor-like protein 6) (AtELP6) (BP80-like protein d) (AtBP80d)	29	29.3.4.3	Protein.targeting.secretory pathway.vacuole
Os06g0694200	OsGELP88	Esterase%2C SGNH hydrolase-type domain containing protein	26	26.28	Misc.GDSL-motif lipase
Os06g0716750		Hypothetical gene	35	35	Unknown
Os06g0717850		Non-protein coding transcript	35	35	Unknown
Os06g0728902	OsGSL6	Similar to predicted protein	3	3.6	Minor CHO metabolism
Os07g0100800	OsProT3	Similar to Amino acid permease	34	34.3	Transport.amino acids
Os07g0107300		Plant disease resistance response protein family protein	20	20.1.7	Stress.biotic.PR-proteins
Os07g0119800		Protein of unknown function DUF538 domain containing protein	35	35.2	Unknown

Os07g0136866		Similar to BRI1-KD interacting protein 133 (Fragment)	35	35	Unknown
Os07g0155600	EIN2	Nramp ion-transporter family protein%2C Ethylene signaling pathwa	34	34.12	Transport.metal
Os07g0176400		Hypothetical protein	35	35	Unknown
Os07g0179700		Hypothetical conserved gene	29	29.5	Protein.degradation
Os07g0184850		Similar to histone H1	35	35	Unknown
Os07g0203275		Ribosomal protein S1%2C RNA-binding domain domain containing protein	29	29.2.1	Protein.synthesis_ribosomal protein
Os07g0204400		Conserved hypothetical protein	35	35.2	Unknown
Os07g0206400	pro13a.1	13 kDa prolamin precursor	33	33.1	Development.storage proteins
Os07g0206500	pro13a.2	13 kDa prolamin precursor	33	33.1	Development.storage proteins
Os07g0212400		Transposase%2C Ptta/En/Spm%2C plant domain containing protein	4	4.1.6	Protein.postranslational modification.kinase.receptor like cytoplasmatic kinase X
Os07g0219250	pro13b.1	Similar to cDNA%2C clone: J090058L24%2C full insert sequence	33	33.1	Development.storage proteins
Os07g0219400	pro13b.3	Prolamin precursor	33	33.1	Development.storage proteins
Os07g0226200		Hypothetical protein	35	35.2	Unknown
Os07g0243150		Hypothetical conserved gene	35	35.2	Unknown
Os07g0277200		Conserved hypothetical protein	35	35.2	Unknown
Os07g0413800		Conserved hypothetical protein	35	35.2	Unknown
Os07g0419300		Similar to Thaumatin-like protein	20	20.1	Stress.biotic
Os07g0448400	OsPIP2	Similar to Aquaporin PIP2-5	34	34.19.1	Transport.Major Intrinsic Proteins.PIP
Os07g0451101		Hypothetical conserved gene	35	35	Unknown
Os07g0453132		Conserved hypothetical protein	35	35	Unknown
Os07g0504200		Hypothetical conserved gene	27	27.2	RNA.transcription
Os07g0509800	OsAPRL1	Similar to adenosine 5'-phosphosulfate reductase 2	14	14.2	S-assimilation.APR
Os07g0523400	OsGPT2-3	Glucose-6-phosphate/phosphate-translocator precursor	34	34.8	Transport.metabolite transporters at the envelope membrane
Os07g0523600	OsGPT2	Similar to Glucose-6-phosphate/phosphate-translocator precursor	34	34.8	Transport.metabolite transporters at the envelope membrane
Os07g0523965		Similar to Glucose-6-phosphate/phosphate-translocator precursor	34	34.8	Transport.metabolite transporters at the envelope membrane
Os07g0524200		Harpin-induced 1 domain containing protein	33	33.2	Development.late embryogenesis abundant
Os07g0529600	OsDR8	Similar to Thiazole biosynthetic enzyme 1-1%2C chloroplast precursor	18	18.2	Co-factor and vitamine metabolism.thiamine
Os07g0546050		Non-protein coding transcript	35	35	Unknown
Os07g0553700		Similar to Light induced protein like	35	35.2	Unknown
Os07g0564750		Conserved hypothetical protein	30	30.5	Signalling.G-proteins
Os07g0567500		Similar to predicted protein	35	35.2	Unknown
Os07g0587450		Non-protein coding transcript	35	35	Unknown
Os07g0595750		Similar to predicted protein	35	35	Unknown
Os07g0599900		Conserved hypothetical protein	35	35.2	Unknown
Os07g0609100		Hypothetical conserved gene	35	35	Unknown
Os07g0624700		UMP/CMP kinase a (EC 2.7.1.48)	23	23.4.3	Nucleotide metabolism.phosphotransfer and pyrophosphatases.uridylate kinase
Os07g0633200		Similar to SC35-like splicing factor SCL30a%2C 30a kD	27	27.1.1	RNA.processing.splicing
Os07g0634400		HAD-superfamily hydrolase%2C subfamily IA%2C variant 3 domain containing protein	35	35.2	Unknown
Os07g0647550		Non-protein coding transcript	35	35	Unknown
Os07g0675200	OsEnS-114	Conserved hypothetical protein	35	35.2	Unknown
Os07g0679050		Hypothetical protein	35	35	Unknown

Os07g0684000		Ricin B-related lectin domain containing protein	33	33	Development
Os08g0104400		Conserved hypothetical protein	35	35.2	Unknown
Os08g0104900		Protein of unknown function DUF6%2C transmembrane domain containing protein	34	34.8	Transport.metabolite transporters at the envelope membrane
Os08g0106501		Protein of unknown function DUF679 family protein	35	35.2	Unknown
Os08g0106700		Similar to 10A19I.15	35	35	Unknown
Os08g0109300	AK1	Homolog of Arabidopsis ADENOSINE MONOPHOSPHATE KINASE 2 (AMK2)%2C A member of adenylate kinase (AK) famil	23	23.4.1	Nucleotide metabolism.phosphotransfer and pyrophosphatases.adenylate kinase
Os08g0112700	MADS26	MADS-box transcription factor%2C Regulator of both biotic and abiotic stress response	27	27.3.24	RNA.regulation of transcription.MADS box transcription factor family
Os08g0157600	CCA1	MYB transcription factor%2C Circadian cloc	27	27.3.26	RNA.regulation of transcription.MYB-related transcription factor family
Os08g0184700		Conserved hypothetical protein	35	35	Unknown
Os08g0207500	OsZIP4	Similar to Zinc transporter ZIP1 (Fragment)	34	34.12	Transport.metal
Os08g0237500		Non-protein coding transcript	35	35	Unknown
Os08g0243866		Similar to chromatin remodeling complex subunit	27	27.3.44	RNA.regulation of transcription.Chromatin Remodeling Factors
Os08g0269000		Hypothetical conserved gene	35	35.2	Unknown
Os08g0300501		Conserved hypothetical protein	35	35	Unknown
Os08g0374000	OsBetvI	Bet v I allergen family protein	8	8.3	TCA / org. transformation
Os08g0377500		Protein of unknown function DUF599 family protein	35	35.2	Unknown
Os08g0382400	OsCYP38	Peptidyl-prolyl cis-trans isomerase%2C cyclophilin-type domain containing protein	29	29.6	Protein.folding
Os08g0383750		Hypothetical genes	35	35	Unknown
Os08g0386125		Hypothetical protein	35	35	Unknown
Os08g0413500		Hypothetical conserved gene	35	35	Unknown
Os08g0414900		Hypothetical gene	35	35	Unknown
Os08g0415400		Conserved hypothetical protein	35	35	Unknown
Os08g0431800		Conserved hypothetical protein	35	35	Unknown
Os08g0433050		Similar to Arginine decarboxylase	35	35.2	Unknown
Os08g0459333		Hypothetical gene	35	35	Unknown
Os08g0472800	OsABA8OX2	Cytochrome P450 family protein	26	26.1	Misc.misc2
Os08g0499250		Hypothetical conserved gene	35	35	Unknown
Os08g0500400		Hypothetical protein	35	35	Unknown
Os08g0502000		Conserved hypothetical protein	27	27.3.67	RNA.regulation of transcription.putative transcription regulator
Os08g0508500	JMJ717	Similar to predicted protein	27	27.3.57	RNA.regulation of transcription.JUMONJI family
Os08g0518800	OsGH	Similar to Class III chitinase homologu	20	20.1	Stress.biotic
Os08g0531000	NPP1	Nucleotide pyrophosphatase/phosphodiesterase%2C Negative effect on plant growth and starch accumulatio	26	26.13	Misc.acid and other phosphatases
Os08g0532350		Hypothetical gene	35	35	Unknown
Os08g0536300	OsBBX26	Similar to Hd1	27	27.3.7	RNA.regulation of transcription.C2C2(Zn) CO-like, Constans-like zinc finger family
Os08g0546800	HSFB2B	Similar to Heat stress transcription factor B-2b	27	27.3.23	RNA.regulation of transcription.HSF,Heat-shock transcription factor family
Os08g0557800		Similar to Fatty acyl coA reductase	11	11.9.4.13	Lipid metabolism.lipid degradation.beta-oxidation.acyl CoA reductase
Os09g0252700		Similar to E3 ubiquitin protein ligase UPL1 (EC 6.3.2.-) (Ubiquitin-protein ligase 1)	29	29.5	Protein.degradation
Os09g0261400		Protein of unknown function DUF1296 family protein	35	35.2	Unknown

Os09g0294000		Similar to Bifunctional aspartokinase/homoserine dehydrogenase 2%2C chloroplast precursor (AK-HD 2) (AK-HSDH 2) [Includes: Aspartokinase (EC 2.7.2.4)%3B Homoserine dehydrogenase (EC 1.1.1.3)]	13	13.1.3.6.1.1	Amino acid metabolism.synthesis.aspartate family.misc.homoserine.aspartate kinase
Os09g0294300	OsUBC40	Ubiquitin-conjugating enzyme/RWD-like domain containing protein	29	29.5.11.3	Protein.degradation.ubiquitin.E2
Os09g0321000		Conserved hypothetical protein	35	35.2	Unknown
Os09g0322000		Similar to PaMst-1	35	35.2	Unknown
Os09g0327000		Hypothetical conserved gene	35	35.2	Unknown
Os09g0331900		Hypothetical conserved gene	35	35.2	Unknown
Os09g0346500	CAB1R	Similar to Chlorophyll a-b binding protein%2C chloroplast precursor (LHCII type I CAB) (LHCP)	1	1.1.1.1	Photosyntheisis
Os09g0364400		Conserved hypothetical protein	35	35.2	Unknown
Os09g0391501		Hypothetical gene	35	35.2	Unknown
Os09g0397800		Hypothetical gene	35	35	Unknown
Os09g0401600		Similar to PUX2 (PLANT UBX DOMAIN-CONTAINING PROTEIN 2)%3B nucleic acid binding / zinc ion binding	27	27.3.99	RNA.regulation of transcription.unclassified
Os09g0412300		Similar to Calmodulin-like protein	35	35.2	Unknown
Os09g0417600	WRKY76	WRKY transcription factor%2C Transcriptional repressor%2C Pathogen defens	27	27.3.32	RNA.regulation of transcription.WRKY domain transcription factor family
Os09g0425500		Hypothetical gene	35	35.2	Unknown
Os09g0437500		Dormancyauxin associated family protein	35	35.2	Unknown
Os09g0444500	OsBRXL5	Similar to Protein Brevis radix-like 1	20	20	Stress
Os09g0458700		EF hand domain containing protein	35	35.2	Unknown
Os09g0512700	NEF	Armadillo-like helical domain containing protein	34	34	Transport
Os09g0514500		Conserved hypothetical protein	35	35.2	Unknown
Os09g0514550		Hypothetical protein	35	35	Unknown
Os09g0515550		Hypothetical protein	35	35.2	Unknown
Os09g0522000	OsDREB1B	Similar to Dehydration-responsive element-binding protein 1B	17	17.5.2	Hormone metabolism.ethylene.signal transduction
Os09g0526600	HSFB2C	Similar to Isoform 2 of Heat stress transcription factor B-2c	27	27.3.23	RNA.regulation of transcription.HSF,Heat-shock transcription factor family
Os09g0528200	Oshox6	Similar to Homeobox-leucine zipper protein HOX6	27	27.3.22	RNA.regulation of transcription.HB,Homeobox transcription factor family
Os09g0538000	OsRNS5	Ribonuclease T2 family protein	27	27.1.19	RNA.processing.ribonucleases
Os09g0552900	ONAC110	Similar to NAC domain protein%2C IPR003441	33	33.99	Development.unspecified
Os09g0554101		Hypothetical conserved gene	35	35	Unknown
Os09g0558050		Non-protein coding transcript	35	35	Unknown
Os10g0154566		Hypothetical conserved gene	35	35	Unknown
Os10g0156200		Similar to Protein kinase domain containing protein	29	29.4	Protein.postranslational modification
Os10g0195250		Conserved hypothetical protein	35	35.2	Unknown
Os10g0325400	OsREM0.2	Remorin%2C C-terminal domain containing protein	33	33	Development
Os10g0340450		Similar to Putative defensin-like protein 134	35	35.2	Unknown
Os10g0364900		Conserved hypothetical protein	35	35.2	Unknown
Os10g0388701		Non-protein coding transcript	35	35	Unknown
Os10g0389500		Conserved hypothetical protein	35	35.2	Unknown
Os10g0390550		Non-protein coding transcript	35	35	Unknown
Os10g0392600	OsSPX3	SPX domain-containing protein%2C Negative regulation of phosphate signaling%2C Pi homeostasi	20	20.2	Stress.abiotic
Os10g0450900	GRP	Similar to Glycine-rich cell wall structural protein 2 precursor	10	10	Cell wall
Os10g0452500		Similar to Glycine-rich protein precursor	35	35.2	Unknown

Os10g0456800	DCA1	CHY zinc finger protein%2C Transcriptional co-activator of DST (Drought and Salt Tolerance: zinc finger transcription factor gene)%2C Drought and salt tolerance%2C Stomatal aperture contro	29	29.5.11.4.2	Protein.degradation.ubiquitin.E3.RING
Os10g0472400		Similar to Thioredoxin	35	35.2	Unknown
Os10g0489000		Similar to FIP1	17	17.1.3	Hormone metabolism.abscisic acid.induced-regulated-responsive-activated
Os10g0509600		Conserved hypothetical protein	35	35.2	Unknown
Os10g0511800		Similar to Plus-3 domain containing protein%2C expressed	35	35	Unknown
Os10g0544933		Non-protein coding transcript	35	35	Unknown
Os10g0550066	OsMED12_2	Hypothetical conserved gene	35	35	Unknown
Os10g0550400		Conserved hypothetical protein	35	35.2	Unknown
Os10g0562000		Similar to glycoprotein	35	35.2	Unknown
Os10g0565401		Hypothetical conserved gene	35	35	Unknown
Os10g0579150		Hypothetical gene	35	35	Unknown
Os11g0111900		BTB domain containing protein	29	29.5.11.4.5.2	Protein.degradation.ubiquitin.E3.BTB/POZ Cullin3.BTB/POZ
Os11g0133700		Similar to Glucosamine-fructose-6-phosphate aminotransferase%2C isomerizing family protein%2C expressed	3	3.5	Minor CHO metabolism
Os11g0138600		Similar to Phosphoglycerate mutase family protein	4	4.3.12	Glycolysis
Os11g0151700	OsPAP21b	Purple acid phosphatase (EC:3.1.3.2)%2C Improvement in phosphate acquisition and utilizatio	26	26.13	Misc.acid and other phosphatases
Os11g0151800	OsPAP20a	Similar to ATPAP18/PAP18	26	26.13	Misc.acid and other phosphatases
Os11g0153100		Conserved hypothetical protein	35	35.2	Unknown
Os11g0168250		Hypothetical gene	35	35	Unknown
Os11g0172133		Hypothetical conserved gene	35	35	Unknown
Os11g0195800		Conserved hypothetical protein	35	35.2	Unknown
Os11g0210500	Adh2*	Similar to Alcohol dehydrogenase	26	26.11.1	Misc.alcohol dehydrogenases
Os11g0235400		Hypothetical genes	35	35	Unknown
Os11g0244200		Similar to Pisum sativum 17.9 kDa heat shock protein (hsp17.9) (Fragment)	20	20.2	Stress.abiotic
Os11g0249800		Non-protein coding transcript	35	35	Unknown
Os11g0434800		Conserved hypothetical protein	35	35	Unknown
Os11g0447300	OsRALF-1	GTP-binding protein%2C HSR1-related domain containing protein	30	30.5	Signalling.G-proteins
Os11g0474100		Conserved hypothetical protein	35	35	Unknown
Os11g0496500		Similar to AT.I.24-5 protein (Fragment)	35	35.2	Unknown
Os11g0518600		Conserved hypothetical protein	35	35.2	Unknown
Os11g0546500	OsGT6	Galactosyl transferase family protein	26	26.2	Misc.UDP glucosyl and glucoronyl transferases
Os11g0547000	FKF1	Autonomous floral activator%2C Promotion of flowerin	29	29.5.11.4.3.2	Protein.degradation.ubiquitin.E3.SCF.FBOX
Os11g0595200		PAP fibrillin family protein	31	31.1	Cell organisation
Os11g0620150		Hypothetical gene	35	35	Unknown
Os11g0621200		Similar to OSIGBa0117N13.1 protein	13	13	Amino acid metabolism
Os11g0629100		Conserved hypothetical protein	35	35	Unknown
Os11g0641300		Conserved hypothetical protein	35	35.2	Unknown
Os11g0707000	OsRcaII	Similar to Ribulose bisphosphate carboxylase/oxygenase activase%2C chloroplastic	1	1.3.13	Photosyntheisis
Os11g0709000		Similar to H0124E07.4 protein	35	35	Unknown
Os12g0104766		Similar to Clathrin heavy chain	31	31	Cell organisation

Os12g0108500	OsFbox636	Similar to Leucine Rich Repeat family protein%2C expressed	29	29.5.11.4.3.2	Protein.degradation.ubiquitin.E3.SCF.FBOX
Os12g0111500		BTB domain containing protein	29	29.5.11.4.5.2	Protein.degradation.ubiquitin.E3.BTB/POZ Cullin3.BTB/POZ
Os12g0112251		Hypothetical gene	35	35	Unknown
Os12g0112500	OsPP124	Protein-tyrosine phosphatase%2C dual specificity domain containing protein	2	2.2.2.10	major CHO metabolism
Os12g0114100	OsWNK8	Similar to MAP kinase-like protein	29	29.4	Protein.postranslational modification
Os12g0135100		Similar to Phosphoglycerate mutase family protein	4	4.3.12	Glycolysis
Os12g0139400	RR10	A-type response regulator%2C Cytokinin signalin	27	27.3.5	RNA.regulation of transcription.ARR
Os12g0151000	OsPAP20b	Similar to Purple acid phosphatase	26	26.13	Misc.acid and other phosphatases
Os12g0156000	OsS40-11	Similar to OSIGBa0134P10.11 protein	35	35.2	Unknown
Os12g0183100		Similar to Branched chain alpha-keto acid dehydrogenase E1-alpha subunit (Fragment)	35	35	Unknown
Os12g0189300		Pyruvate/Phosphoenolpyruvate kinase%2C catalytic core domain containing protein	27	27.3.99	RNA.regulation of transcription.unclassified
Os12g0204550		Hypothetical gene	35	35	Unknown
Os12g0210601		Hypothetical conserved gene	35	35	Unknown
Os12g0226400		Similar to 2-alkenal reductase	26	26.7	Misc.oxidases - copper, flavone etc.
Os12g0244150		Hypothetical protein	35	35	Unknown
Os12g0269100	pro13a.3	Similar to Prolamin	33	33.1	Development.storage proteins
Os12g0274700	RBCS	Similar to Petunia ribulose 1%2C5-bisphosphate carboxylase small subunit mRNA (clone pSSU 51)%2C partial cds. (Fragment)	1	1.3.2	Photosyntheisis
Os12g0288801		Conserved hypothetical protein	35	35	Unknown
Os12g0291100	RbcS3	Similar to Petunia ribulose 1%2C5-bisphosphate carboxylase small subunit mRNA (clone pSSU 51)%2C partial cds. (Fragment)	16	16.2	Secondary metabolism.phenylpropanoids
Os12g0493900		Armadillo-like helical domain containing protein	35	35.2	Unknown
Os12g0548401		Similar to Proteinase inhibitor	35	35.2	Unknown
Os12g0548700		Similar to MPI	35	35.2	Unknown
Os12g0575300		Conserved hypothetical protein	35	35	Unknown
Os12g0575400		Conserved hypothetical protein	35	35.2	Unknown
Os12g0576100		Conserved hypothetical protein	35	35.2	Unknown
Os12g0583700	RZF71	Zinc finger%2C C2H2-type domain containing protein	27	27.3.11	RNA.regulation of transcription.C2H2 zinc finger family
Os12g0586100	SAPK9	Serine/threonine protein kinase%2C Abscisic acid (ABA)-activated protein kinase%2C Hyperosmotic stress response%2C ABA signal transductio	29	29.4	Protein.postranslational modification
Os12g0610600	OMTN3	NAC transcription factor%2C Negative regulation of drought toleranc	33	33.99	Development.unspecified
Os12g0630600		Carbohydrate/puine kinase%2C PfkB%2C conserved site containing protein	29	29.2.1	Protein.synthesis_ribosomal protein
