# Supplementary material for: Compensatory Modulation of Seed Storage Protein Synthesis and Alteration of Starch Accumulation by Selective Editing of 13 kDa Prolamin Genes by CRISPR-Cas9 in Rice
Source: Int J Mol Sci. 2024 Jun 14;25(12):6579. doi: 10.3390/ijms25126579 (PMC11204006; doi:10.3390/ijms25126579)
Supplement: Supplementary file 1 [file ijms-25-06579-s001.zip › Supplementary Tables.zip!/Table S7.pdf]

Table S7. Correlation with 13 kDa prolamin genes

No	Gene ID	Name	Description	BINcode		Function	Cluster	Correlation with prolamin gene (I r I ≥ 0.7)	
								Positive correlation gene	**Negative correlation gene**
1	Os07g0419300		Similar to Thaumatin-like protein	20	20.1	Stress.biotic	Cluster 1	*Pro13a.3, Pro13b.5, Pro13b.10, Pro13b.17, Pro13b.19*	
2	Os03g0276500	OsHsp71.1	Similar to Heat shock protein 70	20	20.2.1	Stress	Cluster 1	*Pro13a.3, Pro13b.5, Pro13b.10, Pro13b.17, Pro13b.19*	
3	Os05g0460000	OsctHSP70-1	Similar to 70 kDa heat shock cognate protein 1	20	20.2.1	Stress	Cluster 1	*Pro13a.3, Pro13b.5*	
4	Os10g0392600	OsSPX3	SPX domain-containing protein%2C Negative regulation of phosphate signaling%2C Pi homeostasi	20	20.2	Stress.abiotic	Cluster 1	*Pro13a.3*	
5	Os04g0173800		Lectin precursor (Agglutinin)	20	20.1	Stress.biotic	Cluster 1	*Pro13a.3*	
6	Os05g0519700	HSP101	Heat shock protein%2C Long-term acquired thermotoleranc	20	20.2.1	Stress	Cluster 1	*Pro13b.5*	
7	Os01g0840100	OsMed37_1	Heat shock protein Hsp70 family protein	20	20.2.1	Stress	Cluster 1	*Pro13a.3, Pro13b.5*	
8	Os06g0513050	OsTHI3	Conserved hypothetical protein	20	20.1	Stress.biotic	Cluster 1	*Pro13b.5*	
9	Os06g0514100	Osthi7	Conserved hypothetical protein	20	20.1	Stress.biotic	Cluster 1	*Pro13b.5*	
10	Os02g0134200	OsSGL	DUF1645 family protein%2C Regulation of stress-tolerance and grain lengt	20	20	Stress	Cluster 1	*Pro13a.3, Pro13b.6, Pro13b.14*	
11	Os07g0107300		Plant disease resistance response protein family protein	20	20.1.7	Stress.biotic.PR-proteins	Cluster 1	*Pro13a.3, Pro13b.5, Pro13b.10, Pro13b.17, Pro13b.19*	
12	Os03g0293000	OsERdj3A	Similar to DnaJ domain containing protein%2C expressed	20	20.2.1	Stress	Cluster 1	*Pro13a.3, Pro13b.5, Pro13b.10, Pro13b.13 Pro13b.17, Pro13b.19*	
13	ENSRNA049470806	U1	U1 spliceosomal RNA	27	27.1	RNA.processing	Cluster 1	*Pro13b.16, Pro13b.18, Pro13b.22*	
14	Os08g0546800	OsHsfB2b	SD17:D154imilar to Heat stress transcription factor B-2b	27	27.3.23	RNA.regulation of transcription.HSF,Heat-shock transcription factor family	Cluster 1	*Pro13a.3, Pro13b.5, Pro13b.10, Pro13b.17, Pro13b.19*	
15	ENSRNA049473876	snoZ103	Small nucleolar RNA Z103	27	27.1	RNA.processing	Cluster 1	*Pro13a.3, Pro13b.5, Pro13b.10, Pro13b.17, Pro13b.19*	
16	Os04g0682400		Similar to H0124B04.13 protein	27	27.1	RNA.processing	Cluster 1	*Pro13a.3, Pro13b.6, Pro13b.10*	
17	ENSRNA049474010	MIR171_1	microRNA MIR171_1	27	27.1	RNA.processing	Cluster 1	*Pro13a.3, Pro13b.5, Pro13b.9, Pro13b.10, Pro13b.13, Pro13b.17, Pro13b.19, Pro13b.20*	
18	ENSRNA049474061	U5	U5 spliceosomal RNA	27	27.1	RNA.processing	Cluster 1	*Pro13a.3, Pro13b.5, Pro13b.10, Pro13b.19*	
19	ENSRNA049476188	snoZ242	Small nucleolar RNA Z242	27	27.1	RNA.processing	Cluster 1	*Pro13b.5, Pro13b.9, Pro13b.14, Pro13b.17, Pro13b.19, Pro13b.20*	
20	ENSRNA049476293	snoZ242	Small nucleolar RNA Z242	27	27.1	RNA.processing	Cluster 1	*Pro13b.1, Pro13b.3, Pro13b.6, Pro13b.9, Pro13b.11, Pro13b.12, Pro13b.14, Pro13b.16, Pro13b.18, Pro13b.21, Pro13b.22*	
22	ENSRNA049467463	U2	U2 spliceosomal RNA	27	27.1	RNA.processing	Cluster 1	*Pro13a.3, Pro13b.5, Pro13b.10, Pro13b.13, Pro13b.17, Pro13b.19*	
23	ENSRNA050013643	Intron_gpII	Group II catalytic intron	27	27.1	RNA.processing	Cluster 1	*Pro13a.3, Pro13b.3*	

25	ENSRNA049472320	U2	U2 spliceosomal RNA	27	27.1	RNA.processing	Cluster 1	*Pro13a.3, Pro13b.10*
26	ENSRNA049469003	snoR44_J54	Small nucleolar RNA R44/J54/Z268 family	27	27.1	RNA.processing	Cluster 1	*Pro13b.5*
28	ENSRNA049468126	snoR44_J54	Small nucleolar RNA R44/J54/Z268 family	27	27.1	RNA.processing	Cluster 1	*Pro13b.6*
29	ENSRNA049473620	SNORD25	Small nucleolar RNA SNORD25	27	27.1	RNA.processing	Cluster 1	*Pro13b.5, Pro13b.17*
30	ENSRNA049476404	snoZ266	Small nucleolar RNA Z266	27	27.1	RNA.processing	Cluster 1	*Pro13a.3, Pro13b.5, Pro13b.10, Pro13b.13, Pro13b.17, Pro13b.19, Pro 13b.20*
31	ENSRNA050013667	Intron_gpII	Group II catalytic intron	27	27.1	RNA.processing	Cluster 1	*Pro13b.5*
32	Os12g0583700	RZF71	Zinc finger%2C C2H2-type domain containing protein	27	27.3.11	RNA.regulation of transcription.C2H2 zinc finger family	Cluster 1	*Pro13a.3, Pro13b.10*
33	ENSRNA049470113	snoR117	small nucleolar RNA snoR117	27	27.1	RNA.processing	Cluster 1	*Pro13b.5*
34	ENSRNA049470425	U54	Small nucleolar RNA U54	27	27.1	RNA.processing	Cluster 1	*Pro13a.3, Pro13b.1, Pro13b.6, Pro13b.9, Pro13b.11, Pro13b.12, Pro13b.13, Pro13b.14, Pro13b.16, Pro13b.21*
35	ENSRNA049469812	U2	U2 spliceosomal RNA	27	27.1	RNA.processing	Cluster 1	*Pro13b.1, Pro13b.3, Pro13b.6, Pro13b.9, Pro13b.11, Pro13b.12, Pro13b.14, Pro13b.16, Pro13b.18, Pro13b.21, Pro13b.22*
36	ENSRNA049467742	MIR1428	microRNA MIR1428	27	27.1	RNA.processing	Cluster 1	*Pro13a.3*
37	ENSRNA049467714	snoR134	small nucleolar RNA snoR134	27	27.1	RNA.processing	Cluster 1	*Pro13a.3, Pro13b.5, Pro13b.10, Pro13b.17, Pro13b.19*
38	Os02g0685200	OsMyb1R	MYB transcription factor%2C Transcriptional activator in mediating stress and rhythm responsivegene expressio	27	27.3.26	RNA.regulation of transcription.MYB-related transcription factor family	Cluster 1	*Pro13a.3, Pro13b.10*
39	ENSRNA049473879	snoZ103	Small nucleolar RNA Z103	27	27.1	RNA.processing	Cluster 1	*Pro13a.3*
40	Os07g0633200		Similar to SC35-like splicing factor SCL30a%2C 30a kD	27	27.1.1	RNA.processing.splicing	Cluster 1	*Pro13a.3, Pro13b.5, Pro13b.10, Pro13b.19*
41	ENSRNA050013672	Intron_gpII	Group II catalytic intron	27	27.1	RNA.processing	Cluster 1	*Pro13b.5, Pro13b.17*
42	Os09g0538000	OsRNS5	Ribonuclease T2 family protein	27	27.1.19	RNA.processing.ribonucleases	Cluster 1	*Pro13a.3, Pro13b.5, Pro13b.10, Pro13b.13, Pro13b.17, Pro13b.19*
46	Os08g0112700	OsMADS26	MADS-box transcription factor%2C Regulator of both biotic and abiotic stress response	27	27.3.24	RNA.regulation of transcription.MADS box transcription factor family	Cluster 1	*Pro13a.3, Pro13b.5, Pro13b.10, Pro13b.19*
47	ENSRNA049473223	SNORD14	Small nucleolar RNA SNORD14	27	27.1	RNA.processing	Cluster 1	*Pro13b.5*
48	ENSRNA049467377	U5	U5 spliceosomal RNA	27	27.1	RNA.processing	Cluster 1	*Pro13b.18, Pro13b.22*
49	Os04g0583900	OsLHY	Similar to LHY protein	27	27.3.26	RNA.regulation of transcription.MYB-related transcription factor family	Cluster 1	*Pro13a.3, Pro13b.5, Pro13b.10, Pro13b.19*
50	ENSRNA049472132	snoZ199	Small nucleolar RNA Z199	27	27.1	RNA.processing	Cluster 1	*Pro13a.3*
51	ENSRNA050013668	Intron_gpII	Group II catalytic intron	27	27.1	RNA.processing	Cluster 1	*Pro13b.5*
52	Os08g0157600	OsCCA1	MYB transcription factor%2C Circadian cloc	27	27.3.26	RNA.regulation of transcription.MYB-related transcription factor family	Cluster 1	*Pro13a.3, Pro13b.5, Pro13b.10, Pro13b.17, Pro13b.19*
53	Os06g0197575		Similar to Chaperonin	29	29.6	Protein.folding	Cluster 1	*Pro13b.5*
54	EPlORYSAT000373805	tRNA-Val (UAC)	tRNA	29	29.2.7	Protein synthesis_transfer RNA	Cluster 1	*Pro13a.3, Pro13b.5, Pro13b.10, Pro13b.17, Pro13b.19*

55	Os02g0102900	OsCPn60beta2	Similar to RuBisCO subunit binding-protein beta subunit%2C chloroplast (60 kDa chaperonin beta subunit) (CPN-60 beta) (Fragment)	29	29.6	Protein.folding	Cluster 1	*Pro13b.18, Pro13b.22*	
56	Os02g0322400		Similar to NPKL2 (Fragment)	29	29.4	Protein.postranslational modification	Cluster 1	*Pro13a.3, Pro13b.6*	
57	Os01g0958200		Curculin-like (mannose-binding) lectin domain containing protein	29	29.4	Protein.postranslational modification	Cluster 1	*Pro13b.1, Pro13b.3, Pro13b.9, Pro13b.11, Pro13b.12, Pro13b.14, Pro13b.16, Pro13b.18, Pro13b.21, Pro13b.22*	
58	Os08g0382400	OsCYP38	Peptidyl-prolyl cis-trans isomerase%2C cyclophilin-type domain containing protein	29	29.6	Protein.folding	Cluster 1	*Pro13b.5, Pro13b.10*	
59	Os04g0450900		Protein kinase PKN/PRK1%2C effector domain containing protein	29	29.4	Protein.postranslational modification	Cluster 1	*Pro13a.3*	
60	EPlORYSAT000373848	tRNA-His (GUG)	tRNA	29	29.2.7	Protein synthesis_transfer RNA	Cluster 1	*Pro13b.1, Pro13b.3, Pro13b.16, Pro13b.18, Pro13b.22*	
61	Os03g0262200		Similar to predicted protein	29	29.4	Protein.postranslational modification	Cluster 1	*Pro13b.18, Pro13b.22*	
63	Os09g0552900	OsNAC110	Similar to NAC domain protein%2C IPR003441	27	27.3	NAC transcription factor	Cluster 1	*Pro13a.3*	
64	Os01g0761400		TGF-beta receptor%2C type I/II extracellular region family protein	34	34.13	transport.peptides and oligopeptides	Cluster 1	*Pro13b.18, Pro13b.22*	
65	Os06g0184766		Hypothetical conserved gene	34	34.16	Transport.ABC transporters and multidrug resistance systems	Cluster 1	*Pro13a.3, Pro13b.5, Pro13b.10, Pro13b.17, Pro13b.19*	
66	Os09g0512700	NEF	Armadillo-like helical domain containing protein	34	34	Transport	Cluster 1	*Pro13a.3, Pro13b.5, Pro13b.10, Pro13b.17, Pro13b.19*	
67	Os01g0593700	OsSultr3	Sulphate transporter domain containing protein	34	34.6	Transport sulphate	Cluster 1	*Pro13b.1, Pro13b.3, Pro13b.6, Pro13b.11, Pro13b.12, Pro13b.14, Pro13b.16, Pro13b.18, Pro13b.21, Pro13b.22*	
68	Os01g0825800	OsATL7	Amino acid transporter%2C transmembrane domain containing protein	34	34.3	Transport.amino acids	Cluster 1	*Pro13b.5, Pro13b.10, Pro13b.17, Pro13b.19*	
69	Os02g0226350		Non-protein coding transcript	34	34.9	Transport.metabolite transporters at the mitochondrial membrane	Cluster 1	*Pro13a.3, Pro13b.5, Pro13b.10*	
70	Os07g0448400	OsPIP2	Similar to Aquaporin PIP2-5	34	34.19.1	Transport.Major Intrinsic Proteins.PIP	Cluster 1	*Pro13b.16, Pro13b.18, Pro13b.22*	
72	Os08g0518800	OsGH	Similar to Class III chitinase homologu	20	20.1	Stress.biotic	Cluster 2		Pro13b.5, Pro13b.10
73	Os06g0513943	OsTHI6	Conserved hypothetical protein	20	20.1	Stress.biotic	Cluster 2		Pro13b.1, Pro13b.3, Pro13b.16, Pro13b.18, Pro13b.22
74	Os03g0726300	OsOSCA2.3	Protein of unknown function DUF221 domain containing protein	20	20.2.3	Stress.abiotic.drought/salt	Cluster 2		Pro13a.3, Pro13b.5, Pro13b.9, Pro13b.10, Pro13b.11, Pro13b.12, Pro13b.13, Pro13b.14, Pro13b.17, Pro13b.19, Pro13b.20, Pro13b.21
75	Os06g0197550		Similar to Chaperonin	20	20.2	Stress.abiotic	Cluster 2		Pro13a.3, Pro13b.1, Pro13b.9, Pro13b.10, Pro13b.11, Pro13b.12, Pro13b.13, Pro13b.14, Pro13b.16, Pro13b.17, Pro13b.19, Pro13b.20, Pro13b.21

76	ENSRNA049475950	snoR135	small nucleolar RNA snoR135	27	27.1	RNA.processing	Cluster 2	Pro13b.1, Pro13b.3, Pro13b.6
77	ENSRNA049473172	SNORD14	Small nucleolar RNA SNORD14	27	27.1	RNA.processing	Cluster 2	Pro13b.5, Pro13b.10
78	ENSRNA049473908	snoR134	small nucleolar RNA snoR134	27	27.1	RNA.processing	Cluster 2	Pro13a.3, Pro13b.5, Pro13b.9, Pro13b.10, Pro13b.13, Pro13b.17, Pro13b.19, Pro13b.20
79	ENSRNA049473164	SNORD14	Small nucleolar RNA SNORD14	27	27.1	RNA.processing	Cluster 2	Pro13b.5, Pro13b.10
80	ENSRNA049470992	snoZ221_snoR21b	Small nucleolar RNA Z221/R21b	27	27.1	RNA.processing	Cluster 2	Pro13a.3, Pro13b.5, Pro13b.10, Pro13b.13, Pro13b.17, Pro13b.19
81	ENSRNA049468467	MIR820	microRNA MIR820	27	27.1	RNA.processing	Cluster 2	Pro13b.5, Pro13b.10
83	ENSRNA049472073	snoZ118	Small nucleolar RNA Z118/Z121/Z120	27	27.1	RNA.processing	Cluster 2	Pro13b.3, Pro13b.6
85	ENSRNA049472195	snoZ118	Small nucleolar RNA Z118/Z121/Z120	27	27.1	RNA.processing	Cluster 2	Pro13a.3, Pro13b.6, Pro13b.14, Pro13b.21
86	ENSRNA049468709	snoR134	small nucleolar RNA snoR134	27	27.1	RNA.processing	Cluster 2	Pro13a.3
87	ENSRNA049473887	snoZ103	Small nucleolar RNA Z103	27	27.1	RNA.processing	Cluster 2	Pro13b.1, Pro13b.3, Pro13b.6, Pro13b.9, Pro13b.11, Pro13b.12, Pro13b.14, Pro13b.16, Pro13b.18, Pro13b.21, Pro13b.22
88	Os06g0558766		Similar to predicted protein	27	27.1	RNA.processing	Cluster 2	Pro13a.3, Pro13b.5, Pro13b.10, Pro13b.17, Pro13b.19
89	ENSRNA050013642	Intron_gpII	Group II catalytic intron	27	27.1	RNA.processing	Cluster 2	Pro13a.3, Pro13b.5, Pro13b.10, Pro13b.17, Pro13b.19
90	ENSRNA049472947	SNORD24	Small nucleolar RNA SNORD24	27	27.1	RNA.processing	Cluster 2	Pro13b.3
91	Os01g0702900		Similar to Sucrose-phosphate synthase (EC 2.4.1.14)	27	27.1	RNA.processing	Cluster 2	Pro13b.3, Pro13b.6
92	Os02g0152800	RPB1		27	27.2	RNA.transcription	Cluster 2	Pro13a.3, Pro13b.5, Pro13b.10, Pro13b.17, Pro13b.19
93	ENSRNA049468767	MIR820	microRNA MIR820	27	27.1	RNA.processing	Cluster 2	Pro13a.3
95	ENSRNA049476411	snoZ266		27	27.1	RNA.processing	Cluster 2	Pro13b.1, Pro13b.3, Pro13b.6, Pro13b.14
96	ENSRNA049465412	snoR60	Small nucleolar RNA snoR60	27	27.1	RNA.processing	Cluster 2	Pro13b.9, Pro13b.11, Pro13b.12, Pro13b.13, Pro13b.14, Pro13b.16, Pro13b.17, Pro13b.19, Pro13b.20, Pro13b.21
97	ENSRNA049471778	LSU_rRNA_eukarya	Eukaryotic large subunit ribosomal RNA	27	27.1	RNA.processing	Cluster 2	Pro13a.3
98	Os01g0733200	OsHsfC1b	Similar to Heat shock transcription factor 29 (Fragment)	27	27.3.23	RNA.regulation of transcription.HSF,Heat-shock transcription factor family	Cluster 2	Pro13b.5, Pro13b.10
99	Os01g0971800	OsPCL1	Transcription factor with a GARP DNA-binding domain%2C Photoperiodic control of flowering time%2C Clock associated-componen	27	27.3.20	RNA.regulation of transcription.G2-like transcription factor family, GARP	Cluster 2	Pro13a.3, Pro13b.5, Pro13b.10, Pro13b.17, Pro13b.19
100	ENSRNA049467117	mir-166	mir-166 microRNA precursor	27	27.1	RNA.processing	Cluster 2	Pro13a.3
101	ENSRNA049472003	snoZ118	Small nucleolar RNA Z118/Z121/Z120	27	27.1	RNA.processing	Cluster 2	Pro13a.3, Pro13b.5, Pro13b.10, Pro13b.17, Pro13b.19

102	ENSRNA049471381	MIR159	microRNA MIR159	27	27.1	RNA.processing	Cluster 2	Pro13b.17, Pro13b.20
104	ENSRNA049473128	SNORD14	Small nucleolar RNA SNORD14	27	27.1	RNA.processing	Cluster 2	Pro13b.3
105	ENSRNA049475720	U54	Small nucleolar RNA U54	27	27.1	RNA.processing	Cluster 2	Pro13a.3, Pro13b.5, Pro13b.10
106	ENSRNA050013650	Intron_gpII	Group II catalytic intron	27	27.1	RNA.processing	Cluster 2	Pro13a.3
107	ENSRNA049472907	SNORD14	Small nucleolar RNA SNORD14	27	27.1	RNA.processing	Cluster 2	Pro13b.3
108	ENSRNA049447972	tRNA-Trp	tRNA-Trp for anticodon CCA	29	29.2.7	Protein synthesis_transfer RNA	Cluster 2	Pro13a.3
109	ENSRNA049449267	tRNA-Trp	tRNA-Trp for anticodon CCA	29	29.2.7	Protein synthesis_transfer RNA	Cluster 2	Pro13a.3
110	ENSRNA049445376	tRNA-Trp	tRNA-Trp for anticodon CCA	29	29.2.7	Protein synthesis_transfer RNA	Cluster 2	Pro13a.3
111	EPIORYSAT000373795	tRNA-Thr (GGU)	tRNA	29	29.2.7	Protein synthesis_transfer RNA	Cluster 2	Pro13a.3, Pro13b.5, Pro13b.9, Pro13b.10, Pro13b.13,Pro13b.14, Pro13b.17, Pro13b.19, Pro13b.20, Pro13b.21
112	ENSRNA049448151	tRNA-Trp	tRNA-Trp for anticodon CCA	29	29.2.7	Protein synthesis_transfer RNA	Cluster 2	Pro13a.3
113	Os10g0456800	DCA1	CHY zinc finger protein%2C Transcriptional co-activator of DST (Drought and Salt Tolerance: zinc finger transcription factor gene)%2C Drought and salt tolerance%2C Stomatal aperture contro	29	29.5.11.4.2	Protein.degradation.ubiquitin.E3.RING	Cluster 2	Pro13a.3, Pro13b.5
114	Os04g0538166		Similar to Elongation factor G%2C chloroplastic	29	29.2.4	Protein.synthesis.elongation	Cluster 2	Pro13a.3, Pro13b.5, Pro13b.10, Pro13b.17, Pro13b.19
115	ENSRNA049445795	tRNA-Gln	tRNA-Gln for anticodon CUG	29	29.2.7	Protein synthesis_transfer RNA	Cluster 2	Pro13a.3
116	EPIORYSAT000373835	tRNA-Arg (UCU)	tRNA	29	29.2.7	Protein synthesis_transfer RNA	Cluster 2	Pro13a.3
117	Os01g0148000		E3 ubiquitin-protein ligase	29	29.5.11	Protein degradation ubiquitin	Cluster 2	Pro13a.3, Pro13b.5, Pro13b.10, Pro13b.13, Pro13b.17, Pro13b.19
118	ENSRNA049447201	tRNA-Trp	tRNA-Trp for anticodon CCA	29	29.2.7	Protein synthesis_transfer RNA	Cluster 2	Pro13a.3
119	Os12g0586100	SAPK9	Serine/threonine protein kinase%2C Abscisic acid (ABA)-activated protein kinase%2C Hyperosmotic stress response%2C ABA signal transductio	29	29.4	Protein.postranslational modification	Cluster 2	Pro13a.3, Pro13b.5, Pro13b.10, Pro13b.17, Pro13b.19
120	Os09g0252700		Similar to E3 ubiquitin protein ligase UPL1 (EC 6.3.2.-) (Ubiquitin-protein ligase 1)	29	29.5	Protein.degradation	Cluster 2	Pro13a.3, Pro13b.5, Pro13b.10, Pro13b.17, Pro13b.19
121	ENSRNA049446836	tRNA-Trp	tRNA-Trp for anticodon CCA	29	29.2.7	Protein synthesis_transfer RNA	Cluster 2	Pro13a.3
122	Os03g0117100	PEX11-1	Peroxisomal protein%2C Response to salt and low nitrogen stresse	29	29.3.5	Protein.targeting.peroxisomes	Cluster 2	Pro13b.5
123	Os07g0203275		Ribosomal protein S1%2C RNA-binding domain domain containing protein	29	29.2.1	Protein.synthesis_ribosomal protein	Cluster 2	Pro13a.3, Pro13b.5, Pro13b.10, Pro13b.17, Pro13b.19
124	ENSRNA049449660	tRNA-Trp	tRNA-Trp for anticodon CCA	29	29.2.7	Protein synthesis_transfer RNA	Cluster 2	Pro13a.3
125	Os07g0179700	DUF239	Hypothetical conserved gene	29	29.5	Protein.degradation	Cluster 2	Pro13a.3
126	Os05g0433100		Similar to Serine/threonine-protein kinase SAPK4	29	29.4	Protein.postranslational modification	Cluster 2	Pro13a.3, Pro13b.14, Pro13b.19
127	EPIORYSAT000373615	trnR	tRNA	29	29.2.7	Protein synthesis_transfer RNA	Cluster 2	Pro13a.3
128	ENSRNA049449070	tRNA-Trp	tRNA-Trp for anticodon CCA	29	29.2.7	Protein synthesis_transfer RNA	Cluster 2	Pro13a.3

129	ENSRNA049445384	tRNA-Trp	tRNA-Trp for anticodon CCA	29	29.2.7	Protein synthesis_transfer RNA	Cluster 2		Pro13a.3
130	ENSRNA049449035	tRNA-Trp	tRNA-Trp for anticodon CCA	29	29.2.7	Protein synthesis_transfer RNA	Cluster 2		Pro13a.3
131	Os09g0294300	OsUBC40	Ubiquitin-conjugating enzyme/RWD-like domain containing protein	29	29.5.11.3	Protein.degradation.ubiquitin.E2	Cluster 2		Pro13a.3, Pro13b.5, Pro13b.10
132	Os11g0547000	FKF1	Autonomous floral activator%2C Promotion of flowerin	29	29.5.11.4.3.2	Protein.degradation.ubiquitin.E3.SCF.FBOX	Cluster 2		Pro13a.3, Pro13b.5, Pro13b.10, Pro13b.17, Pro13b.19
133	Os03g0765400		Similar to nucleoporin p58/p45	29	29.3.1	Protein.targeting.nucleus	Cluster 2		Pro13b.5
134	Os05g0535800		Similar to F14O23.10 protein	29	29.2.1.1.3.1.1	Protein.synthesis.ribosomal protein.prokaryotic.unknown organellar.30S subunit.S1	Cluster 2		Pro13b.1, Pro13b.9, Pro13b.16, Pro13b.18, Pro13b.21, Pro13b.22
135	Os07g0523400	OsGPT2-3	Glucose-6-phosphate/phosphate-translocator precursor	34	34.8	Transport.metabolite transporters at the envelope membrane	Cluster 2		Pro13b.5, Pro13b.10, Pro13b.17, Pro13b.19
136	Os07g0523965		Similar to Glucose-6-phosphate/phosphate-translocator precursor	34	34.8	Transport.metabolite transporters at the envelope membrane	Cluster 2		Pro13b.5, Pro13b.10, Pro13b.17, Pro13b.19
137	Os04g0681600		Protein of unknown function DUF580 family protein	34	34.99	Transport.misc	Cluster 2		Pro13a.3, Pro13b.5, Pro13b.10, Pro13b.13, Pro13b.17, Pro13b.19
138	Os07g0155600	EIN2	Nramp ion-transporter family protein%2C Ethylene signaling pathwa	34	34.12	Transport.metal	Cluster 2		Pro13a.3, Pro13b.5, Pro13b.10, Pro13b.17, Pro13b.19
139	Os04g0659800	OsAAP7B	Similar to OSIGBa0132E09-OSIGBa0108L24.20 protein	34	34.3	Transport.amino acids	Cluster 2		Pro13a.3, Pro13b.5, Pro13b.19
140	Os01g0872100		TGF-beta receptor%2C type I/II extracellular region family protein	34	34.13	Transport.peptides and oligopeptides	Cluster 2		Pro13a.3, Pro13b.5, Pro13b.10, Pro13b.17, Pro13b.19
141	Os08g0207500	OsZIP4	Similar to Zinc transporter ZIP1 (Fragment)	34	34.12	Transport.metal	Cluster 2		Pro13b.10
142	Os05g0156900	Chalk5	Vacuolar H+-translocating pyrophosphatase%2C Regulation of grain chalkines	34	34.3	Transport.amino acids	Cluster 2		Pro13b.1, Pro13b.3, Pro13b.6, Pro13b.9, Pro13b.11, Pro13b.12, Pro13b.14, Pro13b.18, Pro13b.21, Pro13b.22
143	Os07g0523600	OsGPT2	Similar to Glucose-6-phosphate/phosphate-translocator precursor	34	34.8	Transport.metabolite transporters at the envelope membrane	Cluster 2		Pro13b.5, Pro13b.10, Pro13b.17, Pro13b.19
144	Os04g0675400	OsDjC45	Similar to Chaperone protein dnaJ	20	20.2.1	Stress	Cluster 3	*Pro13a.1*	Pro13a.3, Pro13b.5, Pro13b.9,Pro13b.10, Pro13b.11, Pro13b.12, Pro13b.13, Pro13b.17, Pro13b.19, Pro13b.20, Pro13b.21
145	Os03g0245800	OsHSP26	Similar to Heat shock protein 26	20	20.2.1	Stress	Cluster 3	*Pro13a.1*	
146	Os04g0445100	HSP22a	Similar to 22.7 kDa class IV heat shock protein precursor	20	20.2.1	Stress	Cluster 3	*Pro13a.1*	Pro13b.10, pro13b.13
147	Os11g0244200	HSP20	Similar to Pisum sativum 17.9 kDa heat shock protein (hsp17.9) (Fragment)	20	20.2	Stress.abiotic	Cluster 3	*Pro13a.1*	Pro13b.13
148	Os04g0107900	Hsp90	Similar to Heat shock protein 82	20	20.2.1	Stress	Cluster 3	*Pro13a.1*	
149	Os03g0776900	OsDjC35	Similar to mitochondrial import inner membrane translocase subunit TIM14	20	20.2.1	Stress	Cluster 3	*Pro13a.1*	
150	Os03g0267200	OsHsp17.7	Low molecular mass heat shock protein Oshsp17.7	20	20.2.1	Stress	Cluster 3	*Pro13a.1*	

151	ENSRNA049468908	snoU31b	Small nucleolar RNA U31b	27	27.1	RNA.processing	Cluster 3	*Pro13a.1*	Pro13b.13
152	Os03g0409400	MOB1A	Similar to mps one binder kinase activator-like 1A	27	27.3.99	RNA.regulation of transcription.unclassified	Cluster 3	*Pro13a.1*	Pro13b.5, Pro13b.10, Pro13b.13, Pro13b.17, Pro13b.19
153	Os09g0417600	WRKY76	WRKY transcription factor%2C Transcriptional repressor%2C Pathogen defens	27	27.3.32	RNA.regulation of transcription.WRKY domain transcription factor family	Cluster 3	*Pro13a.1*	Pro13a.3, Pro13b.5, Pro13b.9,Pro13b.10, Pro13b.11, Pro13b.12, Pro13b.13, Pro13b.14, Pro13b.17, Pro13b.19, Pro13b.20
154	Os08g0508500	JMJ717	Similar to predicted protein	27	27.3.57	RNA.regulation of transcription.JUMONJI family	Cluster 3	*Pro13a.1*	Pro13a.3, Pro13b.5, Pro13b.10, Pro13b.13, Pro13b.17, Pro13b.19
155	ENSRNA049464941	snoR86	Small nucleolar RNA snoR86	27	27.1	RNA.processing	Cluster 3	*Pro13a.1*	Pro13b.13
156	ENSRNA049466121	snoZ168	Small nucleolar RNA Z168/Z174	27	27.1	RNA.processing	Cluster 3	*Pro13a.1*	
157	ENSRNA049468722	snoZ223	Small nucleolar RNA Z223	27	27.1	RNA.processing	Cluster 3	*Pro13a.1*	Pro13b.13
158	ENSRNA049472231	snoZ118	Small nucleolar RNA Z118/Z121/Z120	27	27.1	RNA.processing	Cluster 3	*Pro13a.1*	Pro13b.1, Pro13b.3, Pro13b.6, Pro13b.11, Pro13b.12, Pro13b.13, Pro13b.14, Pro13b.16, Pro13b.21
159	ENSRNA049473120	snoU31b	Small nucleolar RNA U31b	27	27.1	RNA.processing	Cluster 3	*Pro13a.1*	Pro13a.3, Pro13b.1, Pro13b.3, Pro13b.6, Pro13b.9, Pro13b.11, Pro13b.12, Pro13b.13, Pro13b.14, Pro13b.16, Pro13b.21
160	ENSRNA049470386	snoZ278	Small nucleolar RNA Z278	27	27.1	RNA.processing	Cluster 3	*Pro13a.1*	Pro13b.13
161	ENSRNA049470195	MIR171_1	microRNA MIR171_1	27	27.1	RNA.processing	Cluster 3	*Pro13a.1*	Pro13b.13
162	Os08g0243866	CHR743	Similar to chromatin remodeling complex subunit	27	27.3.44	RNA.regulation of transcription.Chromatin Remodeling Factors	Cluster 3	*Pro13a.1*	Pro13a.3, Pro13b.5, Pro13b.10, Pro13b.13, Pro13b.17, Pro13b.19
163	ENSRNA049465951	snoZ169	Small nucleolar RNA Z169	27	27.1	RNA.processing	Cluster 3	*Pro13a.1*	Pro13a.3, Pro13b.6, Pro13b.9, Pro13b.10, Pro13b.11, Pro13b.12, Pro13b.13, Pro13b.14, Pro13b.19
164	ENSRNA049472208	snoZ118	Small nucleolar RNA Z118/Z121/Z120	27	27.1	RNA.processing	Cluster 3	*Pro13a.1*	Pro13b.13
166	Os09g0528200	Oshox6	Similar to Homeobox-leucine zipper protein HOX6	27	27.3.22	RNA.regulation of transcription.HB,Homeobox transcription factor family	Cluster 3	*Pro13a.1*	Pro13a.3, Pro13b.5, Pro13b.9, Pro13b.10, Pro13b.11, Pro13b.12, Pro13b.13, Pro13b.14, Pro13b.17, Pro13b.19, Pro13b.20, Pro13b.21
167	ENSRNA049472018	snoR11	Small nucleolar RNA R11/Z151	27	27.1	RNA.processing	Cluster 3	*Pro13a.1*	Pro13b.13
168	ENSRNA049472690	snoZ105	Small nucleolar RNA Z105	27	27.1	RNA.processing	Cluster 3	*Pro13a.1*	Pro13a.3, Pro13b.1, Pro13b.3, Pro13b.6, Pro13b.9, Pro13b.11, Pro13b.12, Pro13b.13, Pro13b.14, Pro13b.16, Pro13b.19, Pro13b.20, Pro13b.21
169	Os04g0684800	OsUEV1D	Similar to CROC-1-like protein (Fragment)	29	29.5.11.3	Protein.degradation.ubiquitin.E2	Cluster 3	*Pro13a.1*	Pro13b.5, Pro13b.10, Pro13b.17, Pro13b.19
170	Os12g0108500	OsFbox636	Similar to Leucine Rich Repeat family protein%2C expressed	29	29.5.11.4.3.2	Protein.degradation.ubiquitin.E3.SCF.FBOX	Cluster 3	*Pro13a.1*	
171	EPlORYSAT000373840	tRNA-Asp (GUC)	tRNA	29	29.2.7	Protein synthesis_transfer RNA	Cluster 3	*Pro13a.1*	Pro13b.13

172	Os10g0156200		Similar to Protein kinase domain containing protein	29	29.4	Protein.postranslational modification	Cluster 3	*Pro13a.1*	Pro13a.3, Pro13b.5, Pro13b.9, Pro13b.10, Pro13b.11, Pro13b.12, Pro13b.13, Pro13b.14, Pro13b.17, Pro13b.19, Pro13b.20, Pro13b.21
173	Os03g0764300	OsMAPKKK18	Serine/threonine protein kinase domain containing protein	29	29.4	Protein.postranslational modification	Cluster 3	*Pro13a.1*	Pro13a.3, Pro13b.1, Pro13b.3, Pro13b.6, Pro13b.9, Pro13b.11, Pro13b.12, Pro13b.13, Pro13b.14, Pro13b.16, Pro13b.19, Pro13b.20, Pro13b.21
174	Os06g0664300		Similar to Vacuolar sorting receptor 6 precursor (AtVSR6) (Epidermal growth factor receptor-like protein 6) (AtELP6) (BP80-like protein d) (AtBP80d)	29	29.3.4.3	Protein.targeting.secretory pathway.vacuole	Cluster 3	*Pro13a.1*	Pro13b.10
176	ENSRNA049476335	snosnR60_Z15	Small nucleolar RNA snR60/Z15/Z230/Z193/J17	27	27.1	RNA.processing	Cluster 4	*Pro13b.6*	Pro13a.1
177	ENSRNA049468200	U1	U1 spliceosomal RNA	27	27.1	RNA.processing	Cluster 4	*Pro13b.1, Pro13b.3, Pro13b.6, Pro13b.9, Pro13b.11, Pro13b.12, Pro13b.13, Pro13b.14, Pro13b.16, Pro13b.18, Pro13b.20, Pro13b.21, Pro13b.22*	Pro13a.1
178	ENSRNA049473180	SNORD14	Small nucleolar RNA SNORD14	27	27.1	RNA.processing	Cluster 4	*Pro13b.6, Pro13b.14*	Pro13a.1
179	ENSRNA049469734	snoZ279_R105_R108	Small nucleolar RNA Z279/snoR105/snoR108	27	27.1	RNA.processing	Cluster 4	*Pro13b.1, Pro13b.3, Pro13b.6, Pro13b.9, Pro13b.11, Pro13b.12, Pro13b.13, Pro13b.14, Pro13b.16, Pro13b.18, Pro13b.20, Pro13b.21, Pro13b.22*	Pro13a.1
180	Os04g0673300	RR6	A-type response regulator%2C Cytokinin signalin	27	27.3.5	RNA.regulation of transcription.ARR	Cluster 4	*Pro13a.3, Pro13b.1, Pro13b.3, Pro13b.6, Pro13b.9, Pro13b.14, Pro13b.16, Pro13b.21*	Pro13a.1
182	ENSRNA050013666	Intron_gpII	Group II catalytic intron	27	27.1	RNA.processing	Cluster 4	*Pro13a.3, Pro13b.6, Pro13b.10, Pro13b.14, Pro13b.17, Pro13b.19*	Pro13a.1
183	Os12g0139400	RR10	A-type response regulator%2C Cytokinin signalin	27	27.3.5	RNA.regulation of transcription.ARR	Cluster 4	*Pro13b.1, Pro13b.3, Pro13b.6, Pro13b.9, Pro13b.11, Pro13b.12, Pro13b.13, Pro13b.14, Pro13b.16, Pro13b.18, Pro13b.20, Pro13b.21, Pro13b.22*	Pro13a.1
184	ENSRNA050013661	Intron_gpII	Group II catalytic intron	27	27.1	RNA.processing	Cluster 4	*Pro13b.1, Pro13b.3, Pro13b.6, Pro13b.9, Pro13b.11, Pro13b.12, Pro13b.13, Pro13b.14, Pro13b.16, Pro13b.18, Pro13b.20, Pro13b.21, Pro13b.22*	Pro13a.1
185	Os11g0111900		BTB domain containing protein	29	29.5.11.4.5.2	Protein.degradation.ubiquitin.E3.BTB/POZ Cullin3.BTB/POZ	Cluster 4	*Pro13b.1, Pro13b.3, Pro13b.6, Pro13b.9, Pro13b.11, Pro13b.12, Pro13b.14, Pro13b.16, Pro13b.21*	Pro13a.1

186	EPlORYSAT000373619	trnD	tRNA	29	29.2.7	Protein synthesis_transfer RNA	Cluster 4	*Pro13b.1, Pro13b.3, Pro13b.6, Pro13b.9, Pro13b.11, Pro13b.12, Pro13b.13, Pro13b.14, Pro13b.16, Pro13b.18, Pro13b.20, Pro13b.21, Pro13b.22*	Pro13a.1
187	Os02g0688800		Non-protein coding transcript	29	29.5.11.4.2	Protein.degradation.ubiquitin.E3.RING	Cluster 4	*Pro13a.3, Pro13b.1, Pro13b.3, Pro13b.6, Pro13b.9, Pro13b.11, Pro13b.12, Pro13b.13, Pro13b.14, Pro13b.16, Pro13b.18, Pro13b.19, Pro13b.20, Pro13b.21, Pro13b.22*	Pro13a.1
188	Os12g0114100	OsWNK8	Similar to MAP kinase-like protein	29	29.4	Protein.postranslational modification	Cluster 4	*Pro13b.3, Pro13b.6, Pro13b.14*	Pro13a.1
189	Os12g0111500		BTB domain containing protein	29	29.5.11.4.5.2	Protein.degradation.ubiquitin.E3.BTB/POZ Cullin3.BTB/POZ	Cluster 4	*Pro13b.1, Pro13b.3, Pro13b.6, Pro13b.9, Pro13b.11, Pro13b.12, Pro13b.13, Pro13b.14, Pro13b.16, Pro13b.21*	Pro13a.1
190	Os08g0104900		Protein of unknown function DUF6%2C transmembrane domain containing protein	34	34.8	Transport.metabolite transporters at the envelope membrane	Cluster 4	*Pro13a.3, Pro13b.3, Pro13b.6, Pro13b.14*	Pro13a.1
191	Os02g0191300	OsATL6	Similar to Amino acid transporter-like protein	34	34.3	Transport.amino acids	Cluster 4	*Pro13b.1, Pro13b.3, Pro13b.6, Pro13b.9, Pro13b.11, Pro13b.12, Pro13b.13, Pro13b.14, Pro13b.16, Pro13b.18, Pro13b.20, Pro13b.21, Pro13b.22*	Pro13a.1
192	ENSRNA049468027	SNORD36	Small nucleolar RNA SNORD36	27	27.1	RNA.processing	Cluster 5	*Pro13a.2*	Pro13b.1, Pro13b.9, Pro13b.11, Pro13b.12, Pro13b.13, Pro13b.16, Pro13b.18, Pro13b.20, Pro13b.21, Pro13b.22
193	ENSRNA049465332	U54	Small nucleolar RNA U54	27	27.1	RNA.processing	Cluster 5	*Pro13a.2*	Pro13b.20
194	ENSRNA049472543	MIR1428	microRNA MIR1428	27	27.1	RNA.processing	Cluster 5	*Pro13a.2*	Pro13b.1, Pro13b.3, Pro13b.9, Pro13b.11, Pro13b.12, Pro13b.13, Pro13b.14, Pro13b.16, Pro13b.18, Pro13b.20, Pro13b.21, Pro13b.22
195	ENSRNA049473610	SNORD25	Small nucleolar RNA SNORD25	27	27.1	RNA.processing	Cluster 5	*Pro13a.2*	
196	ENSRNA049471325	snoZ102_R77	Small nucleolar RNA Z102/R77	27	27.1	RNA.processing	Cluster 5	*Pro13a.2*	Pro13b.20
197	ENSRNA049473842	snoZ266	Small nucleolar RNA Z266	27	27.1	RNA.processing	Cluster 5	*Pro13a.2*	
198	Os09g0444500	OsBRXL5	Similar to Protein Brevis radix-like 1	20	20	Stress	Cluster 6	*Pro13b.1, Pro13b.9, Pro13b.11, Pro13b.12, Pro13b.13, Pro13b.14, Pro13b.16, Pro13b.18, Pro13b.20, Pro13b.21, Pro13b.22*	Pro13a.2
199	Os01g0606900	OsDjC10	Heat shock protein DnaJ%2C N-terminal domain containing protein	20	20.2.1	Stress.abiotic.heat	Cluster 6	*Pro13b.18, Pro13b.22*	Pro13a.2
200	ENSRNA049470973	U12	U12 minor spliceosomal RNA	27	27.1	RNA.processing	Cluster 6	*Pro13b.1, Pro13b.3, Pro13b.9, Pro13b.11, Pro13b.12, Pro13b.16, Pro13b.18, Pro13b.20, Pro13b.21, Pro13b.22*	Pro13a.2
201	ENSRNA049467734	snoZ278	Small nucleolar RNA Z278	27	27.1	RNA.processing	Cluster 6	*Pro13b.1, Pro13b.9, Pro13b.11, Pro13b.12, Pro13b.13, Pro13b.14, Pro13b.16, Pro13b.18, Pro13b.20, Pro13b.21, Pro13b.22*	Pro13a.2

202	ENSRNA049471602	Plant_SRP	Plant signal recognition particle RNA	27	27.1	RNA.processing	Cluster 6	*Pro13b.1*, *Pro13b.3*, *Pro13b.9*, *Pro13b.11*, *Pro13b.12*, *Pro13b.13*, *Pro13b.14*, *Pro13b.16*, *Pro13b.18*, *Pro13b.20*, *Pro13b.21*, *Pro13b.22*	Pro13a.2
203	ENSRNA049464972	snoR86	Small nucleolar RNA snoR86	27	27.1	RNA.processing	Cluster 6	*Pro13b.1*, *Pro13b.3*, *Pro13b.9*, *Pro13b.11*, *Pro13b.12*, *Pro13b.14*, *Pro13b.16*, *Pro13b.18*, *Pro13b.20*, *Pro13b.21*, *Pro13b.22*	Pro13a.2
204	ENSRNA050013664	Intron_gpII	Group II catalytic intron	27	27.1	RNA.processing	Cluster 6	*Pro13b.1*, *Pro13b.3*, *Pro13b.11*, *Pro13b.12*, *Pro13b.16*, *Pro13b.18*, *Pro13b.20*, *Pro13b.21*, *Pro13b.22*	Pro13a.2
205	ENSRNA049473627	SNORD25	Small nucleolar RNA SNORD25	27	27.1	RNA.processing	Cluster 6	*Pro13b.1*, *Pro13b.3*, *Pro13b.9*, *Pro13b.11*, *Pro13b.12*, *Pro13b.14*, *Pro13b.16*, *Pro13b.18*, *Pro13b.21*, *Pro13b.22*	Pro13a.2
206	ENSRNA049473918	U1	U1 spliceosomal RNA	27	27.1	RNA.processing	Cluster 6	*Pro13b.18*, *Pro13b.22*	Pro13a.2
207	ENSRNA049471032	SNORD14	Small nucleolar RNA SNORD14	27	27.1	RNA.processing	Cluster 6	*Pro13b.1*, *Pro13b.3*, *Pro13b.11*, *Pro13b.12*, *Pro13b.16*, *Pro13b.18*, *Pro13b.21*, *Pro13b.22*	Pro13a.2
208	ENSRNA049469217	U1	U1 spliceosomal RNA	27	27.1	RNA.processing	Cluster 6	*Pro13b.1*, *Pro13b.9*, *Pro13b.11*, *Pro13b.12*, *Pro13b.16*, *Pro13b.18*, *Pro13b.20*, *Pro13b.21*, *Pro13b.22*	Pro13a.2
209	ENSRNA049467928	U1	U1 spliceosomal RNA	27	27.1	RNA.processing	Cluster 6	*Pro13b.1*, *Pro13b.3*, *Pro13b.9*, *Pro13b.11*, *Pro13b.12*, *Pro13b.16*, *Pro13b.18*, *Pro13b.20*, *Pro13b.21*, *Pro13b.22*	Pro13a.2
210	ENSRNA049473260	U1	U1 spliceosomal RNA	27	27.1	RNA.processing	Cluster 6	*Pro13b.18*, *Pro13b.22*	Pro13a.2
211	ENSRNA049475930	snoZ159	Small nucleolar RNA Z159/U59	27	27.1	RNA.processing	Cluster 6	*Pro13b.1*, *Pro13b.9*, *Pro13b.11*, *Pro13b.12*, *Pro13b.16*, *Pro13b.18*, *Pro13b.20*, *Pro13b.21*, *Pro13b.22*	Pro13a.2
212	ENSRNA049466137	snoZ163	Small nucleolar RNA Z163/Z177 family	27	27.1	RNA.processing	Cluster 6	*Pro13b.1*, *Pro13b.3*, *Pro13b.9*, *Pro13b.11*, *Pro13b.12*, *Pro13b.14*, *Pro13b.16*, *Pro13b.18*, *Pro13b.21*, *Pro13b.22*	Pro13a.2
213	ENSRNA050013646	Intron_gpII	Group II catalytic intron	27	27.1	RNA.processing	Cluster 6	*Pro13b.1*, *Pro13b.3*, *Pro13b.11*, *Pro13b.12*, *Pro13b.16*, *Pro13b.18*, *Pro13b.21*, *Pro13b.22*	Pro13a.2
214	ENSRNA049471985	snoZ118	Small nucleolar RNA Z118/Z121/Z120	27	27.1	RNA.processing	Cluster 6	*Pro13b.1*, *Pro13b.3*, *Pro13b.11*, *Pro13b.12*, *Pro13b.16*, *Pro13b.18*, *Pro13b.21*, *Pro13b.22*	Pro13a.2
215	ENSRNA049467441	U2	U2 spliceosomal RNA	27	27.1	RNA.processing	Cluster 6	*Pro13b.1*, *Pro13b.3*, *Pro13b.9*, *Pro13b.11*, *Pro13b.12*, *Pro13b.13*, *Pro13b.14*, *Pro13b.16*, *Pro13b.18*, *Pro13b.21*, *Pro13b.22*	Pro13a.2
216	ENSRNA049468702	U6	U6 spliceosomal RNA	27	27.1	RNA.processing	Cluster 6	*Pro13b.1*, *Pro13b.3*, *Pro13b.9*, *Pro13b.11*, *Pro13b.12*, *Pro13b.14*, *Pro13b.16*, *Pro13b.18*, *Pro13b.21*, *Pro13b.22*	Pro13a.2

217	ENSRNA049464985	snoR86	Small nucleolar RNA snoR86	27	27.1	RNA.processing	Cluster 6	*Pro13b.1, Pro13b.3, Pro13b.9, Pro13b.11, Pro13b.12, Pro13b.14, Pro13b.16, Pro13b.18, Pro13b.21, Pro13b.22*	Pro13a.2
218	ENSRNA049475592	U5	U5 spliceosomal RNA	27	27.1	RNA.processing	Cluster 6	*Pro13b.1, Pro13b.3, Pro13b.6, Pro13b.9, Pro13b.11, Pro13b.12, Pro13b.13, Pro13b.14, Pro13b.16, Pro13b.18, Pro13b.20, Pro13b.21, Pro13b.22*	Pro13a.2
219	ENSRNA049468154	U4	U4 spliceosomal RNA	27	27.1	RNA.processing	Cluster 6	*Pro13b.1, Pro13b.3, Pro13b.9, Pro13b.11, Pro13b.12, Pro13b.14, Pro13b.16, Pro13b.18, Pro13b.20, Pro13b.21, Pro13b.22*	Pro13a.2
220	ENSRNA050013665	Intron_gpII	Group II catalytic intron	27	27.1	RNA.processing	Cluster 6	*Pro13b.1, Pro13b.3, Pro13b.11, Pro13b.12, Pro13b.16, Pro13b.18, Pro13b.20, Pro13b.21, Pro13b.22*	Pro13a.2
221	ENSRNA049476340	MIR171_1	microRNA MIR171_1	27	27.1	RNA.processing	Cluster 6	*Pro13b.1, Pro13b.3, Pro13b.9, Pro13b.11, Pro13b.12, Pro13b.14, Pro13b.16, Pro13b.18, Pro13b.20, Pro13b.21, Pro13b.22*	Pro13a.2
222	ENSRNA049466017	snoZ168	Small nucleolar RNA Z168/Z174	27	27.1	RNA.processing	Cluster 6	*Pro13b.1, Pro13b.16, Pro13b.18, Pro13b.19, Pro13b.21, Pro13b.22*	Pro13a.2
223	ENSRNA049475575	snoZ43	Small nucleolar RNA Z43	27	27.1	RNA.processing	Cluster 6	*Pro13b.1, Pro13b.3, Pro13b.9, Pro13b.11, Pro13b.12, Pro13b.14, Pro13b.16, Pro13b.18, Pro13b.20, Pro13b.21, Pro13b.22*	Pro13a.2
224	ENSRNA049470180	U5	U5 spliceosomal RNA	27	27.1	RNA.processing	Cluster 6	*Pro13b.1, Pro13b.3, Pro13b.9, Pro13b.11, Pro13b.12, Pro13b.16, Pro13b.18, Pro13b.20, Pro13b.21, Pro13b.22*	Pro13a.2
225	ENSRNA049465003	snoZ279_R105_R108	Small nucleolar RNA Z279/snoR105/snoR108	27	27.1	RNA.processing	Cluster 6	*Pro13b.1, Pro13b.3, Pro13b.9, Pro13b.11, Pro13b.12, Pro13b.13, Pro13b.14, Pro13b.16, Pro13b.18, Pro13b.20, Pro13b.21, Pro13b.22*	Pro13a.2
226	ENSRNA050013647	Intron_gpII	Group II catalytic intron	27	27.1	RNA.processing	Cluster 6	*Pro13b.1, Pro13b.3, Pro13b.11, Pro13b.12, Pro13b.16, Pro13b.18, Pro13b.20, Pro13b.21, Pro13b.22*	Pro13a.2
227	ENSRNA049475834	U6	U6 spliceosomal RNA	27	27.1	RNA.processing	Cluster 6	*Pro13b.1, Pro13b.9, Pro13b.11, Pro13b.12, Pro13b.13, Pro13b.14, Pro13b.16, Pro13b.18, Pro13b.20, Pro13b.21, Pro13b.22*	Pro13a.2
228	ENSRNA049465347	U54	Small nucleolar RNA U54	27	27.1	RNA.processing	Cluster 6	*Pro13b.1, Pro13b.9, Pro13b.11, Pro13b.12, Pro13b.13, Pro13b.14, Pro13b.16, Pro13b.18, Pro13b.20, Pro13b.21, Pro13b.22*	Pro13a.2

229	ENSRNA049473437	Plant_U3	Plant small nucleolar RNA U3	27	27.1	RNA.processing	Cluster 6	*Pro13b.1, Pro13b.9, Pro13b.11, Pro13b.12, Pro13b.14, Pro13b.16, Pro13b.18, Pro13b.20, Pro13b.21, Pro13b.22*	Pro13a.2
230	ENSRNA049473899	snoZ223	Small nucleolar RNA Z223	27	27.1	RNA.processing	Cluster 6	*Pro13b.18, Pro13b.20, Pro13b.22*	Pro13a.2
231	ENSRNA049472089	snoZ152	Small nucleolar RNA Z152/R70/R12/	27	27.1	RNA.processing	Cluster 6	*Pro13b.1, Pro13b.3, Pro13b.9, Pro13b.11, Pro13b.12, Pro13b.14, Pro13b.16, Pro13b.18, Pro13b.20, Pro13b.21, Pro13b.22*	Pro13a.2
232	ENSRNA050013662	Intron_gpII	Group II catalytic intron	27	27.1	RNA.processing	Cluster 6	*Pro13b.9, Pro13b.11, Pro13b.12, Pro13b.13, Pro13b.14, Pro13b.16, Pro13b.17, Pro13b.18, Pro13b.19, Pro13b.20, Pro13b.21, Pro13b.22*	Pro13a.2
233	ENSRNA049471546	U4	U4 spliceosomal RNA	27	27.1	RNA.processing	Cluster 6	*Pro13b.1, Pro13b.3, Pro13b.9, Pro13b.11, Pro13b.12, Pro13b.13, Pro13b.14, Pro13b.16, Pro13b.18, Pro13b.20, Pro13b.21, Pro13b.22*	Pro13a.2
234	ENSRNA049476209	Plant_U3	Plant small nucleolar RNA U3	27	27.1	RNA.processing	Cluster 6	*Pro13b.1, Pro13b.9, Pro13b.11, Pro13b.12, Pro13b.14, Pro13b.16, Pro13b.18, Pro13b.20, Pro13b.21, Pro13b.22*	Pro13a.2
235	EPlORYSAT000373867	tRNA-Pro (UGG)	tRNA	29	29.2.7	Protein synthesis_transfer RNA	Cluster 6	*Pro13b.18, Pro13b.22*	Pro13a.2
236	EPlORYSAT000373872	tRNA-Ile (GAU)	tRNA	29	29.2.7	Protein synthesis_transfer RNA	Cluster 6	*Pro13b.9, Pro13b.11, Pro13b.12, Pro13b.13, Pro13b.17, Pro13b.20, Pro13b.21*	Pro13a.2
237	EPlORYSAT000373862	tRNA-Ile (GAU)	tRNA	29	29.2.7	Protein synthesis_transfer RNA	Cluster 6	*Pro13b.9, Pro13b.11, Pro13b.12, Pro13b.13, Pro13b.17, Pro13b.20, Pro13b.21*	Pro13a.2
238	gene-rps1		Ribosomal protein S1	29	29.2.1	Protein.synthesis_ribosomal protein	Cluster 6	*Pro13b.1, Pro13b.3, Pro13b.9, Pro13b.11, Pro13b.12, Pro13b.13, Pro13b.14, Pro13b.16, Pro13b.18, Pro13b.20, Pro13b.21, Pro13b.22*	Pro13a.2
239	EPlORYSAT000373829	rRNA	rRNA	29	29.2.6	Protein synthesis-ribosomal RNA	Cluster 6	*Pro13b.1, Pro13b.3, Pro13b.9, Pro13b.11, Pro13b.12, Pro13b.16, Pro13b.18, Pro13b.20, Pro13b.21, Pro13b.22*	Pro13a.2
240	ENSRNA049476508	5_8S_rRNA	5.8S ribosomal RNA	29	29.2.6	Protein synthesis-ribosomal RNA	Cluster 6	*Pro13b.1, Pro13b.3, Pro13b.9, Pro13b.11, Pro13b.12, Pro13b.13, Pro13b.14, Pro13b.16, Pro13b.18, Pro13b.20, Pro13b.21, Pro13b.22*	Pro13a.2
241	ENSRNA049475677	SSU_rRNA_eukarya	Eukaryotic small subunit ribosomal RNA	29	29.2.6	Protein synthesis-ribosomal RNA	Cluster 6	*Pro13b.1, Pro13b.3, Pro13b.9, Pro13b.11, Pro13b.12, Pro13b.14, Pro13b.16, Pro13b.18, Pro13b.20, Pro13b.21, Pro13b.22*	Pro13a.2
242	ENSRNA049475944	LSU_rRNA_eukarya	Eukaryotic large subunit ribosomal RNA	29	29.2.6	Protein synthesis-ribosomal RNA	Cluster 6	*Pro13b.1, Pro13b.3, Pro13b.9, Pro13b.11, Pro13b.12, Pro13b.13, Pro13b.14, Pro13b.16, Pro13b.18, Pro13b.20, Pro13b.21, Pro13b.22*	Pro13a.2

243	ENSRNA049471090	5_8S_rRNA	5.8S ribosomal RNA	29	29.2.6	Protein synthesis-ribosomal RNA	Cluster 6	*Pro13b.1, Pro13b.3, Pro13b.9, Pro13b.11, Pro13b.12, Pro13b.13, Pro13b.14, Pro13b.16, Pro13b.18, Pro13b.20, Pro13b.21, Pro13b.22*	Pro13a.2
244	ENSRNA049476452	SSU_rRNA_eukarya	Eukaryotic small subunit ribosomal RNA	29	29.2.6	Protein synthesis-ribosomal RNA	Cluster 6	*Pro13b.1, Pro13b.3, Pro13b.9, Pro13b.11, Pro13b.12, Pro13b.13, Pro13b.14, Pro13b.16, Pro13b.18, Pro13b.20, Pro13b.21, Pro13b.22*	Pro13a.2
245	EPlORYSAT000373785	tRNA-Ser (UGA)	tRNA	29	29.2.7	Protein synthesis_transfer RNA	Cluster 6	*Pro13b.1, Pro13b.3, Pro13b.9, Pro13b.11, Pro13b.12, Pro13b.14, Pro13b.16, Pro13b.18, Pro13b.20, Pro13b.21, Pro13b.22*	Pro13a.2
246	ENSRNA049473335	SSU_rRNA_eukarya	Eukaryotic small subunit ribosomal RNA	29	29.2.6	Protein synthesis-ribosomal RNA	Cluster 6	*Pro13b.1, Pro13b.3, Pro13b.9, Pro13b.11, Pro13b.12, Pro13b.13, Pro13b.14, Pro13b.16, Pro13b.18, Pro13b.20, Pro13b.21, Pro13b.22*	Pro13a.2
247	ENSRNA049476493	5_8S_rRNA	5.8S ribosomal RNA	29	29.2.6	Protein synthesis-ribosomal RNA	Cluster 6	*Pro13b.1, Pro13b.3, Pro13b.9, Pro13b.11, Pro13b.12, Pro13b.13, Pro13b.14, Pro13b.16, Pro13b.18, Pro13b.20, Pro13b.21, Pro13b.22*	Pro13a.2
248	EPlORYSAT000373849	rRNA	rRNA	29	29.2.6	Protein synthesis-ribosomal RNA	Cluster 6	*Pro13b.1, Pro13b.3, Pro13b.9, Pro13b.11, Pro13b.12, Pro13b.13, Pro13b.14, Pro13b.16, Pro13b.18, Pro13b.20, Pro13b.21, Pro13b.22*	Pro13a.2
249	ENSRNA049466338	LSU_rRNA_eukarya	Eukaryotic large subunit ribosomal RNA	29	29.2.6	Protein synthesis-ribosomal RNA	Cluster 6	*Pro13b.1, Pro13b.3, Pro13b.9, Pro13b.11, Pro13b.12, Pro13b.13, Pro13b.14, Pro13b.16, Pro13b.18, Pro13b.20, Pro13b.21, Pro13b.22*	Pro13a.2
250	ENSRNA050013645	5S_rRNA	5S ribosomal RNA	29	29.2.6	Protein synthesis-ribosomal RNA	Cluster 6	*Pro13b.5, Pro13b.13, Pro13b.17, Pro13b.19, Pro13b.20*	Pro13a.2
251	ENSRNA049476498	5_8S_rRNA	5.8S ribosomal RNA	29	29.2.6	Protein synthesis-ribosomal RNA	Cluster 6	*Pro13b.1, Pro13b.3, Pro13b.9, Pro13b.11, Pro13b.12, Pro13b.13, Pro13b.14, Pro13b.16, Pro13b.18, Pro13b.20, Pro13b.21, Pro13b.22*	Pro13a.2
252	ENSRNA049471043	LSU_rRNA_eukarya	Eukaryotic large subunit ribosomal RNA	29	29.2.6	Protein synthesis-ribosomal RNA	Cluster 6	*Pro13b.1, Pro13b.3, Pro13b.9, Pro13b.11, Pro13b.12, Pro13b.13, Pro13b.14, Pro13b.16, Pro13b.18, Pro13b.20, Pro13b.21, Pro13b.22*	Pro13a.2
253	EPlORYSAT000373811	rRNA	tRNA	29	29.2.7	Protein synthesis_transfer RNA	Cluster 6	*Pro13b.1, Pro13b.3, Pro13b.9, Pro13b.11, Pro13b.12, Pro13b.13, Pro13b.14, Pro13b.16, Pro13b.18, Pro13b.20, Pro13b.21, Pro13b.22*	Pro13a.2
254	ENSRNA049471102	SSU_rRNA_eukarya	Eukaryotic small subunit ribosomal RNA	29	29.2.6	Protein synthesis-ribosomal RNA	Cluster 6	*Pro13b.1, Pro13b.3, Pro13b.9, Pro13b.11, Pro13b.12, Pro13b.13, Pro13b.14, Pro13b.16, Pro13b.18, Pro13b.20, Pro13b.21, Pro13b.22*	Pro13a.2

255	EPIORYSAT000373608	rrn26	rRNA	29	29.2.6	Protein synthesis-ribosomal RNA	Cluster 6	*Pro13b.1, Pro13b.3, Pro13b.9, Pro13b.11, Pro13b.12, Pro13b.13, Pro13b.14, Pro13b.16, Pro13b.18, Pro13b.20, Pro13b.21, Pro13b.22*	Pro13a.2
256	ENSRNA049465309	LSU_rRNA_eukarya	Eukaryotic large subunit ribosomal RNA	29	29.2.6	Protein synthesis-ribosomal RNA	Cluster 6	*Pro13b.1, Pro13b.3, Pro13b.9, Pro13b.11, Pro13b.12, Pro13b.13, Pro13b.14, Pro13b.16, Pro13b.18, Pro13b.20, Pro13b.21, Pro13b.22*	Pro13a.2
257	ENSRNA049468241	LSU_rRNA_eukarya	Eukaryotic large subunit ribosomal RNA	29	29.2.6	Protein synthesis-ribosomal RNA	Cluster 6	*Pro13b.1, Pro13b.9, Pro13b.11, Pro13b.12, Pro13b.16, Pro13b.18, Pro13b.20, Pro13b.21, Pro13b.22*	Pro13a.2
258	EPIORYSAT000373810	tRNA-Tyr (GUA)	tRNA	29	29.2.7	Protein synthesis_transfer RNA	Cluster 6	*Pro13b.1, Pro13b.3, Pro13b.9, Pro13b.11, Pro13b.12, Pro13b.14, Pro13b.16, Pro13b.18, Pro13b.20, Pro13b.21, Pro13b.22*	Pro13a.2
259	ENSRNA049476502	5_8S_rRNA	5.8S ribosomal RNA	29	29.2.6	Protein synthesis-ribosomal RNA	Cluster 6	*Pro13b.1, Pro13b.3, Pro13b.9, Pro13b.11, Pro13b.12, Pro13b.13, Pro13b.14, Pro13b.16, Pro13b.18, Pro13b.20, Pro13b.21, Pro13b.22*	Pro13a.2
260	EPIORYSAT000373839	rRNA	rRNA	29	29.2.6	Protein synthesis-ribosomal RNA	Cluster 6	*Pro13b.1, Pro13b.3, Pro13b.9, Pro13b.11, Pro13b.12, Pro13b.13, Pro13b.14, Pro13b.16, Pro13b.18, Pro13b.20, Pro13b.21, Pro13b.22*	Pro13a.2
261	gene-rpl36		Large ribosomal subunit protein bL36c	29	29.2.1	Protein.synthesis_ribosomal protein	Cluster 6	*Pro13b.9, Pro13b.11, Pro13b.12, Pro13b.13, Pro13b.16, Pro13b.18, Pro13b.20, Pro13b.21, Pro13b.22*	Pro13a.2
262	ENSRNA049474711	5S_rRNA	5S ribosomal RNA	29	29.2.6	Protein synthesis-ribosomal RNA	Cluster 6	*Pro13b.9, Pro13b.11, Pro13b.12, Pro13b.13, Pro13b.16, Pro13b.18, Pro13b.20, Pro13b.21, Pro13b.22*	Pro13a.2
263	ENSRNA049476430	SSU_rRNA_eukarya	Eukaryotic small subunit ribosomal RNA	29	29.2.6	Protein synthesis-ribosomal RNA	Cluster 6	*Pro13b.1, Pro13b.3, Pro13b.9, Pro13b.11, Pro13b.12, Pro13b.13, Pro13b.14, Pro13b.16, Pro13b.18, Pro13b.20, Pro13b.21, Pro13b.22*	Pro13a.2
264	ENSRNA050013644	5S_rRNA	5S ribosomal RNA	29	29.2.6	Protein synthesis-ribosomal RNA	Cluster 6	*Pro13b.5, Pro13b.13, Pro13b.17, Pro13b.19, Pro13b.20*	Pro13a.2
265	EPIORYSAT000373814	tRNA-Ser (GCU)	tRNA	29	29.2.7	Protein synthesis_transfer RNA	Cluster 6	*Pro13b.1, Pro13b.9, Pro13b.11, Pro13b.12, Pro13b.13, Pro13b.14, Pro13b.16, Pro13b.18, Pro13b.20, Pro13b.21, Pro13b.22*	Pro13a.2
266	EPIORYSAT000373852	rRNA	rRNA	29	29.2.6	Protein synthesis-ribosomal RNA	Cluster 6	*Pro13b.1, Pro13b.3, Pro13b.9, Pro13b.11, Pro13b.12, Pro13b.13, Pro13b.14, Pro13b.16, Pro13b.18, Pro13b.20, Pro13b.21, Pro13b.22*	Pro13a.2

267	ENSRNA049468277	LSU_rRNA_eukarya	Eukaryotic large subunit ribosomal RNA	29	29.2.6	Protein synthesis-ribosomal RNA	Cluster 6	*Pro13b.1, Pro13b.3, Pro13b.9, Pro13b.11, Pro13b.12, Pro13b.13, Pro13b.14, Pro13b.16, Pro13b.18, Pro13b.20, Pro13b.21, Pro13b.22*	Pro13a.2
268	EPIORYSAT000373647	rrn18	rRNA	29	29.2.6	Protein synthesis-ribosomal RNA	Cluster 6	*Pro13b.1, Pro13b.3, Pro13b.9, Pro13b.11, Pro13b.12, Pro13b.13, Pro13b.14, Pro13b.16, Pro13b.18, Pro13b.20, Pro13b.21, Pro13b.22*	Pro13a.2
269	Os01g0937100		Similar to Xylanase inhibitor precursor (Xylanase inhibitor TAXI-I)	29	29.5.4	Protein.degradation.aspartate protease	Cluster 6	*Pro13b.1, Pro13b.3, Pro13b.9, Pro13b.11, Pro13b.12, Pro13b.14, Pro13b.16, Pro13b.18, Pro13b.20, Pro13b.21, Pro13b.22*	Pro13a.2
270	ENSRNA049468231	LSU_rRNA_eukarya	Eukaryotic large subunit ribosomal RNA	29	29.2.6	Protein synthesis-ribosomal RNA	Cluster 6	*Pro13b.1, Pro13b.3, Pro13b.9, Pro13b.11, Pro13b.12, Pro13b.13, Pro13b.14, Pro13b.16, Pro13b.18, Pro13b.20, Pro13b.21, Pro13b.22*	Pro13a.2
271	EPIORYSAT000373610	pseudo-trnV	tRNA pseudogene	29	29.2.7	Protein synthesis_transfer RNA	Cluster 6	*Pro13b.5*	Pro13a.2
272	ENSRNA049476447	SSU_rRNA_eukarya	Eukaryotic small subunit ribosomal RNA	29	29.2.6	Protein synthesis-ribosomal RNA	Cluster 6	*Pro13b.1, Pro13b.3, Pro13b.9, Pro13b.11, Pro13b.12, Pro13b.13, Pro13b.14, Pro13b.16, Pro13b.18, Pro13b.20, Pro13b.21, Pro13b.22*	Pro13a.2
273	ENSRNA049476514	5_8S_rRNA	5.8S ribosomal RNA	29	29.2.6	Protein synthesis-ribosomal RNA	Cluster 6	*Pro13b.1, Pro13b.3, Pro13b.9, Pro13b.11, Pro13b.12, Pro13b.13, Pro13b.14, Pro13b.16, Pro13b.18, Pro13b.20, Pro13b.21, Pro13b.22*	Pro13a.2
274	ENSRNA049476444	SSU_rRNA_eukarya	Eukaryotic small subunit ribosomal RNA	29	29.2.6	Protein synthesis-ribosomal RNA	Cluster 6	*Pro13b.1, Pro13b.3, Pro13b.9, Pro13b.11, Pro13b.12, Pro13b.13, Pro13b.14, Pro13b.16, Pro13b.18, Pro13b.20, Pro13b.21, Pro13b.22*	Pro13a.2
275	EPIORYSAT000373834	rRNA	rRNA	29	29.2.6	Protein synthesis-ribosomal RNA	Cluster 6	*Pro13b.1, Pro13b.3, Pro13b.9, Pro13b.11, Pro13b.12, Pro13b.16, Pro13b.18, Pro13b.20, Pro13b.21, Pro13b.22*	Pro13a.2
276	ENSRNA049475757	LSU_rRNA_eukarya	Eukaryotic large subunit ribosomal RNA	29	29.2.6	Protein synthesis-ribosomal RNA	Cluster 6	*Pro13b.1, Pro13b.3, Pro13b.9, Pro13b.11, Pro13b.12, Pro13b.13, Pro13b.14, Pro13b.16, Pro13b.18, Pro13b.20, Pro13b.21, Pro13b.22*	Pro13a.2
277	EPIORYSAT000373657	trnS	tRNA	29	29.2.7	Protein synthesis_transfer RNA	Cluster 6	*Pro13b.1, Pro13b.3, Pro13b.9, Pro13b.11, Pro13b.12, Pro13b.14, Pro13b.16, Pro13b.18, Pro13b.20, Pro13b.21, Pro13b.22*	Pro13a.2
278	ENSRNA049476437	SSU_rRNA_eukarya	Eukaryotic small subunit ribosomal RNA	29	29.2.6	Protein synthesis-ribosomal RNA	Cluster 6	*Pro13b.1, Pro13b.3, Pro13b.9, Pro13b.11, Pro13b.12, Pro13b.13, Pro13b.14, Pro13b.16, Pro13b.18, Pro13b.20, Pro13b.21, Pro13b.22*	Pro13a.2
279	Os02g0563000	OsKMD3	Hypothetical conserved gene	29	29.5.11.4.3.2	Protein.degradation.ubiquitin.E3.SCF.FBOX	Cluster 6	*Pro13b.5*	Pro13a.2

280	ENSRNA049474050	LSU_rRNA_eukarya	Eukaryotic large subunit ribosomal RNA	29	29.2.6	Protein synthesis-ribosomal RNA	Cluster 6	*Pro13b.1*, *Pro13b.3*, *Pro13b.9*, *Pro13b.11*, *Pro13b.12*, *Pro13b.13*, *Pro13b.14*, *Pro13b.16*, *Pro13b.18*, *Pro13b.20*, *Pro13b.21*, *Pro13b.22*	Pro13a.2
281	ENSRNA049475361	5S_rRNA	5S ribosomal RNA	29	29.2.6	Protein synthesis-ribosomal RNA	Cluster 6	*Pro13b.1*, *Pro13b.3*, *Pro13b.9*, *Pro13b.11*, *Pro13b.12*, *Pro13b.14*, *Pro13b.16*, *Pro13b.18*, *Pro13b.20*, *Pro13b.21*, *Pro13b.22*	Pro13a.2
282	EPlORYSAT000373611	rrn5	rRNA	29	29.2.6	Protein synthesis-ribosomal RNA	Cluster 6	*Pro13b.1*, *Pro13b.3*, *Pro13b.9*, *Pro13b.11*, *Pro13b.12*, *Pro13b.13*, *Pro13b.14*, *Pro13b.16*, *Pro13b.18*, *Pro13b.20*, *Pro13b.21*, *Pro13b.22*	Pro13a.2
283	Os12g0630600		Carbohydrate/puine kinase%2C PfkB%2C conserved site containing protein	29	29.2.1	Protein.synthesis_ribosomal protein	Cluster 6	*Pro13b.1*, *Pro13b.3*, *Pro13b.9*, *Pro13b.11*, *Pro13b.12*, *Pro13b.13*, *Pro13b.14*, *Pro13b.16*, *Pro13b.18*, *Pro13b.20*, *Pro13b.21*, *Pro13b.22*	Pro13a.2
284	ENSRNA049465125	LSU_rRNA_eukarya	Eukaryotic large subunit ribosomal RNA	29	29.2.6	Protein synthesis-ribosomal RNA	Cluster 6	*Pro13b.1*, *Pro13b.3*, *Pro13b.9*, *Pro13b.11*, *Pro13b.12*, *Pro13b.13*, *Pro13b.14*, *Pro13b.16*, *Pro13b.18*, *Pro13b.20*, *Pro13b.21*, *Pro13b.22*	Pro13a.2
285	Os07g0100800	OsProT3	Similar to Amino acid permease	34	34.3	Transport.amino acids	Cluster 6	*Pro13b.1*, *Pro13b.3*, *Pro13b.9*, *Pro13b.11*, *Pro13b.12*, *Pro13b.14*, *Pro13b.16*, *Pro13b.18*, *Pro13b.20*, *Pro13b.21*, *Pro13b.22*	Pro13a.2
