# Supplementary material for: Compensatory Modulation of Seed Storage Protein Synthesis and Alteration of Starch Accumulation by Selective Editing of 13 kDa Prolamin Genes by CRISPR-Cas9 in Rice
Source: Int J Mol Sci. 2024 Jun 14;25(12):6579. doi: 10.3390/ijms25126579 (PMC11204006; doi:10.3390/ijms25126579)
Supplement: Supplementary file 1 [file ijms-25-06579-s001.zip › Supplementary Tables.zip!/Table S8.pdf]

Table S8. Primer list

Group	Primer name	Target gene	RAP-DB ID	Product size (bp)	Forward primer (5'→3')	Reverse primer (5'→3')
Design pRGE31-sgRNA vector						
	Vec-Pro13a	Pro13a-I			AGAGTTGTGCAGATGATCCGTGGCAAACGTAGCTGGTTGCCAGAAGTTTTAGAGCTAGAAATAGCAAGTT	
	Vec-Pro13b	Pro13b-I/II			AGAGTTGTGCAGATGATCCGTGGCAAAACGCAGCTGATTGCAAGAGTTTTAGAGCTAGAAATAGCAAGTT	
Screening positive sgRNA clonning						
	Screen-Pro13a	Pro13a-I		226	AAACCACGTGATGTGAAGAA	AAACTTCTGGCAACCAGCTA
	Screen-Pro13b	Pro13b-I/II		222	AAACCACGTGATGTGAAGAA	TAAAACTCTTGCAATCAGCT
in vitro cleavage assay						
	InVitro-pro13a	Pro13a-I		346	TCGTATTTGCTCTCCTTGCT	GATACCACATATGGATGGCAA
	InVitro-pro13b	Pro13b-I/II		433	TCGTCTTTGCTCTCCTTGCT	ACACCGCCAAGGGTGGTAA
Seed storage protein genes.						
	qRT-Pro13a-I	*Pro13a.1*	Os07g0206400	88	CAACTACAGTCGCATCTCCTAC	GGGTTGCCACTATGCTATACTG
		Pro13a.2	Os07g0206500			
	qRT-Pro13a-II	*Pro13a.3*	Os12g0269100	117	TCACCCGTGTTTCAACTGAG	CACAATAGCCTGAACACTGC
		Pro13a.4	Os12g0269200			
	qRT-Pro13b-I	*Pro13b.1*	Os07g0219250	101	ATATTAGGCAATATCAGGTGCAG	GCTTGCCGCAATGCTATACT
		Pro13b.2	Os07g0219300			
		Pro13b.3	Os07g0219400			
		Pro13b.4	Os07g0220000			
	qRT-Pro13b-II	*Pro13b.5*	Os05g0328333	110	TTCACACAGTTCAAGCATTA	AGAGGCGCTGCATGCAGCAA
		Pro13b.6	Os05g0328466			
		Pro13b.7	Os05g0328632			
		Pro13b.8	Os05g0328800			
		Pro13b.9	Os05g0328901			
		Pro13b.10	Os05g0329001			
		Pro13b.11	Os05g0329100			
		Pro13b.12	Os05g0329300			
		Pro13b.13	Os05g0329350			
		Pro13b.14	Os05g0329400			
		Pro13b.15	Os05g0329700			
		Pro13b.16	Os05g0329200			
		Pro13b.17	Os05g0330150			

	Pro13b.18	Os05g0330600			
	Pro13b.19	Os05g0331366			
	Pro13b.20	Os05g0331532			
	Pro13b.21	Os05g0331800			
	Pro13b.22	Os05g0332000			
qRT-Pro16	*Pro16.2*	Os06g0507200	81	CTCAATTTGCCCTCCATGTG	AGAACCGCAATGACCAGTAG
qRT-Pro10	*Pro10.4*	Os11g0535525	97	TTATTTGTGCTGGACTCGGG	GAGAGTTGGAAGTTGACAGGG
qRT-GluA	*GluA-1*	Os01g0762500	234	AATGATGGTGAAGTGCCGGT	TCACGCCTGTATGCTTGAGG
	GluA-2	Os10g0400200			
	GluB-1a	Os02g0249800			
	GluB-1b	Os02g0249900			
qRT-GluB	*GluB-2*	Os02g0249600	104	ATTGAGCAACACTCTGGGCA	TGGCTCTGTAGCCTCTTTGC
	GluB-4	Os02g0268300			
	GluB-5	Os02g0268100			
qRT-GluC	*GluC-1*	Os02g0453600	231	CACAAGGGCCAATAGCCAGA	GGTCACGTACATCACCGTGT
qRT-GluD	*GluD-1*	Os02g0249000	100	AAGACAGAGCGACCAAGCTC	ATGTGCAACACTAGCCGGAA
qRT-Glb	*Globulin*	Os05g0499100	122	AGTCGGAGATGAGGTTCAGG	GAACATCGGCTGGAACCTC
Sarch biosynthesis genes					
qRT-AGPS2	*AGPS2b*	Os08g0345800	211	GCACGAGTGTGCTTGGAATC	GCTCTTGACAGGTGACGGTT
qRT-AGPL2	*AGPL2*	Os01g0633100	204	ATGCTAACTTGGCCCTCACC	CGATGCTAACGCGTGAAGAG
qRT-GBSSI	*GBSSI*	Os06g0133000	197	ACTACCAGCCCAATGGCATC	GATTCCGGCCTTCATCCAGT
qRT-SSI	*SSI*	Os06g0160700	203	TGGCTAGTGAGCAGGAGTCT	CCCGTTCATGTATCTCGGCA
qRT-SSIIa	*SSIIa*	Os06g0229800	199	GCGGTAGAAGAGGAGACGTG	AGGGAGAACATTCAGCAGCC
qRT-SSIIIa	*SSIIIa*	Os08g0191433	202	AGCAGCGAATGGGAGAACAA	AGCGGCCTTGAGTTTCTGTT
qRT-BEI	*BEI*	Os06g0726400	208	CCGAGAGCCATGATCAGTCC	GGATGGCCAAACTCATTGCC
qRT-BEIIa	*BEIIa*	Os04g0409200	210	TGCTGATGGATCCCCTGCTA	CGCAGCGAATTTGGTCGTTT
qRT-BEIIb	*BEIIb*	Os02g0528200	196	GGAATCCAAATGCAGACCGC	TATGGGATTTCTCCTGCGGC
qRT-ISA1	*ISA1*	Os08g0520900	198	GTAGAGTTGATGCCCTGCCA	AATTCCCCGTTTGTGAGCCT
Chaperones and SSP trafficking proteins.					
qRT-BIP-1	*BIP-1*	Os02g0115900	198	ACGATGCTGTCGTGACTGTT	CATCGAATGTCCCACCACCA
qRT-PDIL1-1	*PDIL1-1*	Os11g0199200	188	CCAGTACTTTGGGCTGAGGG	TAACAGGCTCGTCGTTGACC
qRT-PDIL2-3	*PDIL2-3*	Os09g0451500	190	GGCACTGCAAGAAATTGGCA	ACTCTAGCCCCCTGGTAAGG
qRT-CNX	*CNX*	Os04g0402100	207	CGGAGGCTAACAAGCCTGAA	GGATCTCTTGGGGCTTCCAG
qRT-HSP70	*HSP70*	Os01g0840100	106	TGGCATCCTCAACGTCTCT	CATCTTCTCGATCTCCTCCT
qRT-HSP20	*HSP20*	Os11g0244200	144	TGGAGGTGGACGAGGCCA	TGAACCGCCTCCAGAACCT

qRT-HSP90	*HSP90*	Os04g0107900	134	ATCTCCAACTCTTCTGATGC	TCGATGATGGAGAGAGTCTT
Transcription factor					
qRT-HSFB2b	*HSFB2b*	Os08g0546800	149	GAAAGATCTTCTTCGACGGT	CGCAAGTACGCAACTGAAC
qRT-NAC110	*OsNAC110*	Os09g0552900	147	CCAGAACAGGTCAATTGGTA	GAGTCCTGTAACTGAATCCC
qRT-HSFC1b	*HSFC1b*	Os01g0733200	127	AAGACGTTCCACATGGTGAG	GCTTGAAGTAGGAGGGGAGG
qRT-PCL1	*OsPCL1*	Os01g0971800	137	GCACAAGAGGTTCGTGGAG	CGTAGAGGCGATACTTCTGG
qRT-hox6	*OsHox6*	Os09g0528200	122	CAGATCAAGTCGCTGGAGT	TTGTTCTGGAACCAGATGGC
qRT-WRKY76	*WRKY76*	Os09g0417600	117	GTCAAGGACGGGTACCAATG	CACCTTCTTCTTCACCGGG
qRT-RR6	*RR6*	Os04g0673300	122	GTCAACATGATCATCACCGA	ACGTTCTCCGACGACATGAT
qRT-RR10	*RR10*	Os12g0139400	117	AGTAACCACTGTTGATTCGG	ATGATCAGGTTCACTCCAAC
