# Supplementary material for: Compensatory Modulation of Seed Storage Protein Synthesis and Alteration of Starch Accumulation by Selective Editing of 13 kDa Prolamin Genes by CRISPR-Cas9 in Rice
Source: Int J Mol Sci. 2024 Jun 14;25(12):6579. doi: 10.3390/ijms25126579 (PMC11204006; doi:10.3390/ijms25126579)
Supplement: Supplementary file 1 [file ijms-25-06579-s001.zip › Supplementary Tables.zip!/Table S9.pdf]

Table S9. Primer list used in deep-sequencing

| Primer name | Target gene | Product length (bp) | Forward primer (5'→3') | Reverse primer (5'→3') |
|---|---|---|---|---|
| Seq-13a-I | Genes of Pro13a-I subgroup | 398 | TCGTATTTGCTCTCCTTGCT | GATACCACATATGGATGGCAA |
| Seq-13b-I/II | Genes of Pro13b-I/II subgroups | 433 | TCGTCTTTGCTCTCCTTGCT | ACACCGCCAAGGGTGGTAA |
| Seq-13a.1 | *Pro13a.1* | 920 | TTTTCTTGCTACCCATCATG | AGTAAAATTGTAAAGTCCCT |
| Seq-13a.2 | *Pro13a.2* | 849 | TGTATATATGATGGGCATAA | GATAATGCAACCTCATACAT |
| Seq-13a.3 | *Pro13a.3* | 1241 | TGCACATTCAGCTTAGAAAT | TTGAATAGTGGATTTATAAC |
| Seq-13a.4 | *Pro13a.4* | 1243 | AAAACAATAGCTTACAATAC | GTATCCATAATCTCCACACA |
| Seq-13b.1 | *Pro13b.1* | 1432 | AAATCAATTTTCTAGTAGTG | AATAGCTTAGTAAACGTGA |
| Seq-13b.2 | *Pro13b.2* | 1432 | TTTTTCAAGAATGATGGACA | AATAGCTTAGTAAACGTGA |
| Seq-13b.3 | *Pro13b.3* | 1636 | ACTACCCGGTGCACTATAGT | GGGAATAAAAGAATTTAGTC |
| Seq-13b.8 | *Pro13b.8* | 1646 | CCCGTTGGGTTAATTCCGGT | TGAATTACCTATGTCTAGGT |
| Seq-13b.13 | *Pro13b.13* | 854 | CTCACTTAGATCCTAATAGG | AAGTATCTTCGGATCGAATG |
| Seq-13b.A | *Pro13b.5, 6, 7, 9, 10, 11, 12* | 479 | CAGCAAAATAGAAAGATCTA | ACACCGCCAAGGGTGGTAAT |
| Seq-13b.B | *Pro13b.14, 15, 16* | 398 | GCCTCTGCACAGTTTCATGT | ACACCGCCAAGGGTGGTAAT |
| Seq-13b.C | *Pro13b.17, 18, 19* | 1239 | ATCATTTTCGTCTTTGCTCT | ACCATCACGTACACTTTACC |
| Seq-13b.20 | *Pro13b.20* | 1590 | CTTTTACCTCAATCTCACGT | ATGACGTGGAATCACCAGGA |
| Seq-13b.21 | *Pro13b.21* | 1620 | TAGTGTGGAAGATGTGTACA | ACCATCACGTACACTTTCCG |
| Seq-13b.22 | *Pro13b.22* | 1298 | TGCACGTGCATGATTACTAG | GCATCTATAAATAGACAATG |
| Deep-Seq13a | Genes of Pro13a-I subgroup | 217 | **ACACTCTTTCCCTACACGACGCTCTTCCGATCT**TCAGCCCATGCAGTGAGTTC | **GTGACTGGAGTTCAGACGTGTGCTCTTCCGATCT**TGGTAGTGAGATTGTTGCGCT |
| Deep-Seq13b | Genes of Pro13b-I/II subgroups | 216 | **ACACTCTTTCCCTACACGACGCTCTTCCGATCT**CTGCTACAGCAACAGGTGCT | **GTGACTGGAGTTCAGACGTGTGCTCTTCCGATCT**GTGAGATTGTTGCGCCACCA |
| PCR-Cas9 | *SpCas9* | 1839 | GGCAGGATAGACTCTTTGCTGA | TAAGAACCTGCCCAACGAGA |
| PCR-HygR | *HygR* | 615 | GTCTCCGACCTGATGCAGCT | TTGGGAATCCCCGAACATCG |

Bold sequence is linkers for deep sequencing
